# Synthesis and Biological Studies of Novel Aminophosphonates and Their Metal Carbonyl Complexes (Fe, Ru)

**DOI:** 10.3390/ijms23158091

**Published:** 2022-07-22

**Authors:** Aneta Kosińska, David Virieux, Jean-Luc Pirat, Kamila Czarnecka, Małgorzata Girek, Paweł Szymański, Sławomir Wojtulewski, Saranya Vasudevan, Arkadiusz Chworos, Bogna Rudolf

**Affiliations:** 1 Department of Organic Chemistry, Faculty of Chemistry, University of Lodz, Tamka 12, 91-403 Lodz, Poland; 2 ICGM, Univ. Montpellier, CNRS, ENSCM, 34090 Montpellier, France; david.virieux@enscm.fr (D.V.); jean-luc.pirat@enscm.fr (J.-L.P.); 3 Department of Pharmaceutical Chemistry, Drug Analyses and Radiopharmacy, Faculty of Pharmacy, Medical University of Lodz, Muszynskiego 1, 90-151 Lodz, Poland; kamila.czarnecka@umed.lodz.pl (K.C.); pawel.szymanski@umed.lodz.pl (P.S.); 4 Animal House, Faculty of Pharmacy, Medical University of Lodz, Muszynskiego 1, 90-151 Lodz, Poland; malgorzata.girek@umed.lodz.pl; 5 Department of Radiobiology and Radiation Protection, Military Institute of Hygiene and Epidemiology, Kozielska 4, 01-163 Warsaw, Poland; 6 Department of Structural Chemistry, Faculty of Chemistry, University of Bialystok, Ciołkowskiego 1K, 15-245 Bialystok, Poland; slawoj@uwb.edu.pl; 7 Centre of Molecular and Macromolecular Studies, Polish Academy of Sciences, Sienkiewicza 112, 90-363 Lodz, Poland; saranya@cbmm.lodz.pl (S.V.); achworos@cbmm.lodz.pl (A.C.)

**Keywords:** aminophosphonates, metallocarbonyl complexes, serine esterase inhibitors

## Abstract

The quest to find new inhibitors of biologically relevant targets is considered an important strategy to introduce new drug candidates for the treatment of neurodegenerative diseases. A series of (aminomethyl)benzylphosphonates **8a**–**c** and their metallocarbonyl iron **9a**–**c** and ruthenium **10a**–**c** complexes were designed, synthesized, and evaluated for their inhibitory potentials against acetylcholinesterase (AChE) and butyrylcholinesterase (BuChE) by determination of IC_50_. Metallocarbonyl derivatives, in general, did not show significant inhibition activity against these enzymes, the most potent inhibitor was the (aminomethyl)benzylphosphonate **8a** (IC_50_ = 1.215 µM against AChE). Molecular docking analysis of AChE and (aminomethyl)benzylphosphonates **8a**–**c** showed the strongest interactions of **8a** and AChE compared to isomers **8b** and **8c**. Cytotoxicity studies of synthesized compounds towards the V79 cell line were also performed and discussed.

## 1. Introduction

In recent years, considerable efforts have been devoted to finding an effective treatment for Alzheimer’s disease, which is the leading cause of dementia. Acetylcholinesterase (AChE) and butyrylcholinesterase (BuChE), two clinically significant serine esterases, are involved in neurodegenerative processes related to this pathology. Interestingly, inhibition of these enzymes helps to increase cognitive functions, and for this reason, designing and synthesizing potent inhibitors of AChE and BuChE are of great interest [1,2,3,4,5,6].

**Figure 1:**
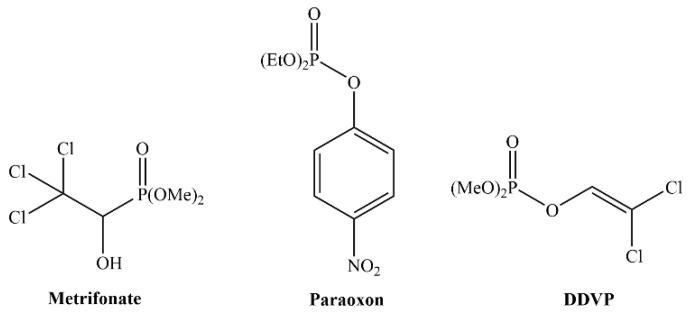
Structures of some organophosphorus-based serine esterase inhibitors.

Several organophosphorus compounds (OP) such as esters, thiol esters, acid halides, or anhydrides of pentavalent phosphorus-containing acids (phosphates and phosphonates) are known to inhibit serine esterases [1,7,8]. The general structure of such inhibitors is R^1^R^2^P(O)X, where R^1^ and R^2^ substituents are usually simple alkyl or aryl groups that can be connected to phosphorus directly or through an intervening -O-, -S- or -NH- linkage [1]. Among thoroughly studied OP serine esterase inhibitors we can lineup DFP, diisopropyl fluorophosphate; paraoxon, diethyl *p*-nitrophenyl phosphate; DDVP, *O*,*O*-dimethyl *O*-(2,2-dichlorovinyl) phosphate; metrifonate, (2,2,2-trichloro-1-hydroxyethyl)phosphonic acid dimethyl ester (Figure 1) [9].

Inhibition of serine esterases by OP generally involves the phosphorylation of the serine residue in the active site. This inhibition is based on the nucleophilic attack of the hydroxyl group of the serine residue on the phosphorus atom of phosphonate forming a covalent bond between the serine and the phosphonate compound. Phosphorylation of the serine results in immediate loss of the leaving group at the phosphorus atom [10].

Both enzymes AChE and BuChE include a catalytic triad in their active site, like that used by serine proteases such as α-chymotrypsin. The essential features of the catalytic site (i.e., a catalytic triad of Ser-His-Glu, an oxyanion hole, a π cation binding site, and an acyl-binding pocket) are the same in both targeted proteins, AChE, and BuChE. According to theoretical and experimental studies inhibitors can interact with either or both of AChE’s binding sites, peripheral anionic site (PAS), and catalytic triad [11,12]. A PAS is a binding site for allosteric modulators and PAS inhibitors at the protein surface where it is situated at the edge of the active-site gorge [13]. PAS contains Tyr72, Tyr124, Trp286, Tyr341, and Asp74 aromatic residues [14]. The PAS-recognized AChE inhibitors or those that interact with both the PAS and the catalytic site may have a dual pharmacological impact. This combination is thought to improve cholinergic neurotransmission while decreasing AChE’s pro-aggregating activity, making it a potentially effective novel treatment for AD [15,16]. The active triad is the major focus for the regulated reduction of AChE in patients with acetylcholine deficiency, such as those with Alzheimer’s disease or myasthenia gravis, which is described by varying muscle weakness and exhaustion as a result of a loss of receptors. On the other hand, organophosphorus (OP) substances such as soman, tabun, sarin, and VX which are known as nerve agents, are also directly bound at the active triad of the AChE enzyme. In addition, some organophosphorus compounds may also have interactions with the peripheral anionic site [17,18,19]. Organophosphorus compounds block the activity of AChE by attaching a phosphorus conjugate covalently to the catalytic serine residue.

The acyl-binding pocket, responsible for the difference in substrate specificity between the two enzymes, is larger in BuChE [20,21,22,23,24]. For example, tetraisopropyl pyrophosphoramide (iso-OMPA) is a selective BuChE inhibitor, where increased steric constraints in the acyl-binding pocket prevent interactions with AChE. The various inhibitors of these two enzymes also bind to different amino acids of the cholinesterase active site and are well characterized. The tricyclic acridine compound, tacrine, and the phenothiazine, ethopropazine, bind to the anionic site [25].

**Scheme 1:**
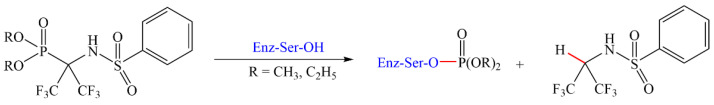
Proposed mode of inhibition of a serine esterase (Enz-Ser-OH) by fluorinated aminophosphonates. Adopted from [1].

Makhaeva et al. reported new fluorinated aminophosphonates that irreversibly inhibit four serine esterases: AChE, BuChE, carboxylesterase, and neuropathy target esterase, a phospholipase that deacetylates intracellular phosphatidylcholine to produce glycerophosphocholine (Scheme 1) [1]. The presence of the two trifluoromethyl groups increased the electrophilic character of phosphonate which has a higher rate of reaction with nucleophiles.

Moreover, α-aminophosphonate derivatives containing a pyrazole moiety showed a significant inhibitory effect on AChE [26]. Finally, another example of a potent inhibitor is the 2,3,4,5-tetrahydrobenzothiazepine-appended α-aminophosphonate derivatives which exhibited similar (BuChE) or better (AChE) inhibitory activity when compared to the standard drug that is galantamine [27].

Metallodrugs are also an efficient and important class of compounds. They are extensively used as active pharmaceutical ingredients (APIs) in the treatment of several pathologies including cancer [28]. The combination of metallic center and bioactive phosphonates could result in compounds with unprecedented structures, and they may potentially combine unique and synergistic properties. Few examples of AChE and BuChE metal-based inhibitors can be found in the literature. Interestingly, they can have better inhibition and pharmacokinetic profiles compared to their parent compounds [29,30]. Jawaria et al. reported ferrocene-based thiosemicarbazones and their transition metal complexes as cholinesterase inhibitors [29]. Another example is the organoruthenium–nitrophen complex which was evaluated for its inhibitory activity against various cholinoesterases [30]. Ristovski et al. found that the [(ⴄ^6^-*p*-cymene)Ru(pyrithionato)Cl] complex reversibly inhibited both AChE and BuChE [31].

**Figure 2:**
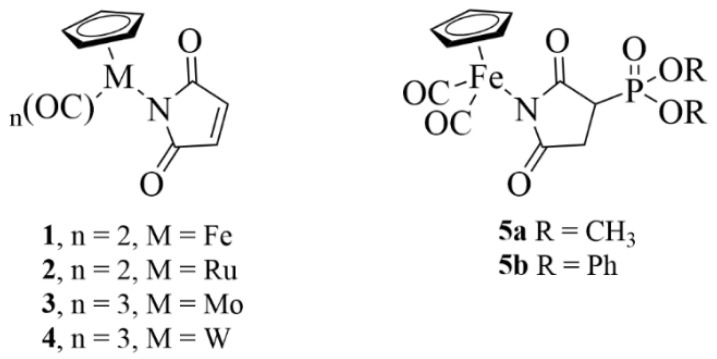
Structure of the metallocarbonyl complexes CpM(CO)_n_(η^1^-*N*-maleimidato) (M = Fe, *n* = 2 (**1**), M = Ru, *n* = 2 (**2**), M = Mo, *n* = 3 (**3**), M = W, *n* = 3 (**4**)) and half-sandwich iron phosphonate complexes (**5a**–**b**).

In one of our works, we studied the metallocarbonyl phosphonate half-sandwich tungsten, iron, and molybdenum complexes obtained by phospha-Michael addition of diethyl- and diphenyl-phosphite to the maleimide ligand of complexes **1**–**4** (Figure 2). Iron complexes **5a**–**b** were screened against AChE and BuChE. The experiments carried out suggested that the diphenyl phosphonate **5b** reversibly inhibits BuChE according to a competitive mechanism and inactivates this enzyme in a time-dependent fashion by phosphonylation of its active site [32].

**Scheme 2:**
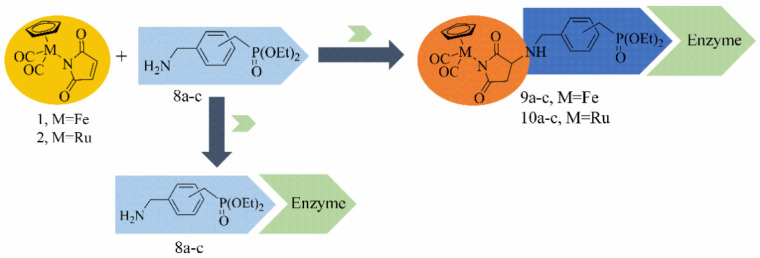
General approach to targeted metallocarbonyl aminophosphonates **9a**–**c** and **10a**–**c**.

To increase the potency of these inhibitors, we designed a hybrid structure based on a diethyl phosphonate motif linked through a spacer (methylenebenzylamine) with metallocarbonyl iron and ruthenium complexes. In this paper, we report an efficient three-step synthetic route to a series of (aminomethyl)benzylphosphonates starting from α,α‘-dibromo or dichloro-*I*-xylene (I = *o*, *m*, *p*). Subsequently, the (aminomethyl)benzylphosphonate **8a**–**c** intermediates were reacted with the maleimide ligand of the CpM(CO)_2_(η^1^-*N*-maleimidato) (M = Fe, Ru) complex to give metallocarbonyl aminophosphonate derivatives **9a**–**c** and **10a**–**c**. The basic idea is the combination of phosphonate properties with the effectiveness of metallocarbonyl complexes as drugs. The overall approach to targeted metallocarbonyl aminophosphonates is depicted in Scheme 2.

The inhibitory activity against AChE and BuChE of the obtained (aminomethyl)benzylphosphonates **8a**–**c** and metallocarbonyl analogs (**9a**–**c**, **10a**–**c**) was evaluated. Interestingly, no data have been reported so far on the biochemical activity of metallocarbonyl complexes containing an aminophosphonate moiety, particularly on their ability to inhibit serine hydrolases. Cytotoxicity studies of compounds **8a**–**c**, **9a**–**c**, and **10a**–**c** toward the V79 cell line were also carried out. Additionally, we have analyzed the interaction of selected phosphonate derivatives (**8a**–**c**) with the AChE protein by theoretical prediction and modeling.

## 2. Results and Discussion

### 2.1. Synthesis of Complexes ***1***–***2***

Half-sandwich complexes (η^5^-cyclopentadienyl)M(CO)_2_(η^1^-*N*-maleimidato) (**1**, M = Fe; **2**, M = Ru), bearing the maleimide moiety, were obtained according to the previously reported photochemical reaction of (η^5^-cyclopentadienyl)M(CO)_2_I (M = Fe, Ru) with maleimide in the presence of diisopropylamine [33,34].

### 2.2. Synthesis of (Aminomethyl)benzylphosphonates ***8a***–***c***

**Scheme 3:**
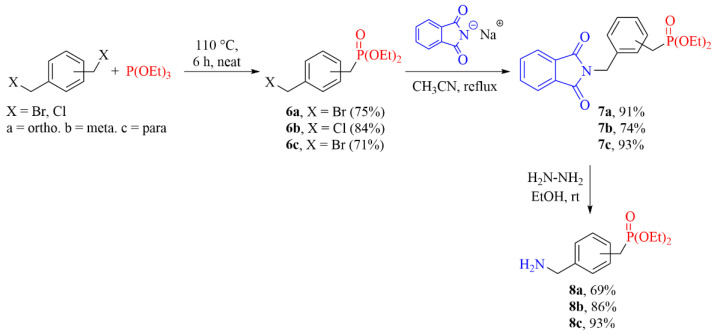
Synthesis of (aminomethyl)benzylphosphonates **8a**–**c**.

Looking for new organometallic scaffolds bearing the aminophosphonate moiety, we designed (aminomethyl)benzylphosphonates **8a**–**c** that are able to react with the maleimide double bond of metallocarbonyl complexes **1** and **2**. The (aminomethyl)benzylphosphonates **8a**–**c** were obtained in a three-step method depicted in Scheme 3.

**Figure 3:**
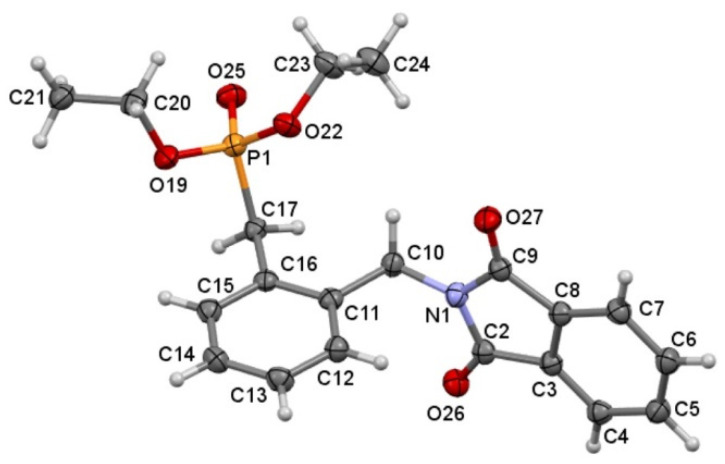
Molecular structure of **7a** with atom-labeling scheme.

**Figure 4:**
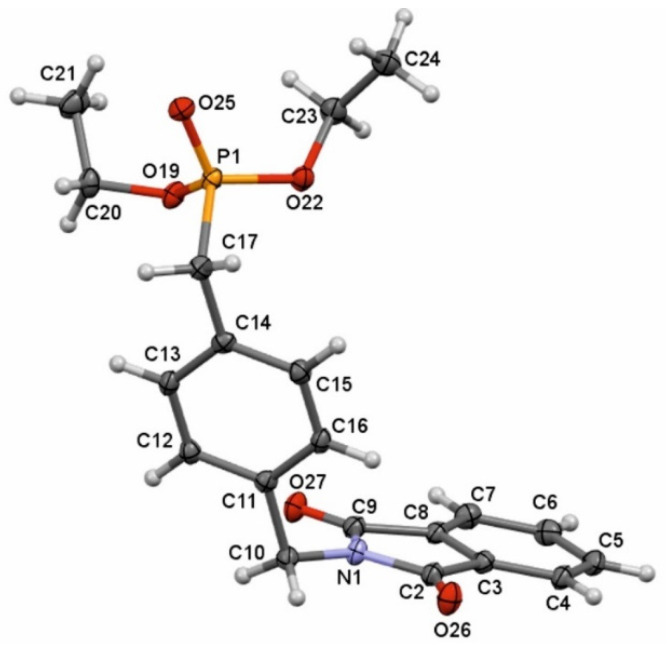
Molecular structure of **7c** with atom-labeling scheme.

In the first step, α,α’-dibromo or dichloro-*I*-xylene (*I* = *o*, *m*, *p*) undergoes the Arbuzov reaction with triethyl phosphite to give diethyl (halogenomethyl)benzylphosphonates **6a**–**c** following the procedure developed by Bessieres et al. [35]. Compounds **6a**–**c** were obtained in high yields (71–84%) and were consecutively characterized by spectroscopic methods (^1^H, ^13^C NMR, IR) and mass spectrometry. For the second step, the diethyl(dioxoisoindolin-2-yl)methyl)benzylphosphonates **7a**–**c** were obtained by reaction of **6a**–**c** with the sodium salt of phthalimide in dry acetonitrile according to the procedure reported by Škopić et al. with modifications [36]. The resulting products **7a**–**c** were obtained in high yields ranging from 74% to 93%. The molecular composition of compounds **7a**–**c** was established by ^1^H, ^13^C NMR, IR, and mass spectrometry, and in the case of compounds **7a** and **7c,** structures were determined by single-crystal X-ray diffraction (Figure 3 and Figure 4).

In the last step, the deprotection by hydrazine of **7a**–**c** was carried out to afford the (aminomethyl)benzylphosphonates **8a**–**c** (Scheme 3) [37]. Compounds **8b** and **8c** were already obtained in a similar way as reported in the patent by Charmant et al., where the α,α‘-dibromo or dichloro-*I*-xylene (*I* = *o*, *m*, *p*) was reacted first with the sodium salt of phthalimide and then with triethylphosphite [38]. ^1^H NMR and ^31^P NMR data of compounds **8b** and **8c** agreed with those previously reported.

#### Crystal Structure Description of Compounds **7a** and **7c**

The molecular structures of both *N*-(phenylmethyl)phthalimide derivatives **7a** and **7c** are presented in Figure 3 and Figure 4, respectively. The asymmetric units in both crystal structures contain a single molecule of each compound. Three fragments can be distinguished in the molecules: a phthalimide part, a phenyl ring, and a diethyl phosphonate group. They are connected to each other by methylene groups. In both crystal structures, the molecules interact with each other through weak C-H^…^O type hydrogen bonds (HBs), where most C-H donors are aliphatic.

The hydrogen bonds arrangement in the crystal structure of **7a**, presented in Figure S57, is based on four C-H^…^O bonds. The first two form an intermolecular cyclic bifurcated acceptor type, where the C10-H10A and C17-H17B bonds of two methylene groups of one molecule play the role of the hydrogen donor, while one oxygen atom (O26) of a neighboring molecule phthalimide fragment acts as an acceptor for both. The second C-H bond of the mentioned methylene group creates the third HB C17-H17A^…^O27, and the oxygen atom O27 of a phthalimide fragment of another molecule acts as an acceptor. The fourth hydrogen bond is formed between the C20-H20B bond of the ethyl group and the O22 oxygen atom of the phosphonate group.

An intermolecular bifurcated acceptor type of HBs can also be observed in the crystal structure of **7c**. The first intermolecular bifurcated acceptor HB connects three molecules, and it is formed by the C10-H10B bond of the methyl group of one molecule, the C4-H4 bond of the phenyl ring of the other, with the oxygen atom O27 of the third molecule acting as the acceptor for both donor bonds. The second intermolecular bifurcated acceptor HB is cyclic and consists of C17-H17B and C20-H20A bonds, with one of the methyl groups, and one of the ethyl groups of one molecule acting as the hydrogen donor and the oxygen atom O25 of the other molecule playing the role of an acceptor. Additionally, C12-H12…O26 HB can be observed, where the C-H bond is aromatic and the oxygen atom O of a phthalimide fragment of another molecule acts as an acceptor. The extended network of hydrogen bonds in the crystal structure of **7c** is shown in Figure S58.

### 2.3. Synthesis of Aminophosphonate Metal Complexes ***9a***–***c*** and ***10a***–***c***

**Scheme 4:**
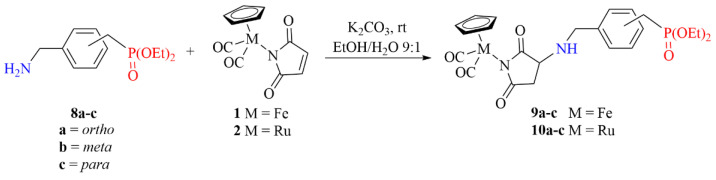
Synthesis of new organometallic phosphonates **9a**–**c** and **10a**–**c**.

The reaction of metallocarbonyl complexes **1**–**2** with aminophosphonates **8a**–**c** was carried out under mild conditions using a mixture of ethanol and distilled water (9:1) as a solvent at room temperature. Half-sandwich iron **9a**–**c** and ruthenium **10a**–**c** aminophosphonate complexes were obtained through the aza-Michael addition of (aminomethyl)benzylphosphonates **8a**–**c** to the electron-deficient double bond of the maleimidato ligand of iron **1** and ruthenium **2** complexes using potassium carbonate as a base (Scheme 4). The aza-Michael reaction of primary amines to maleimide ligands was previously described by us [39] and found applications in the conjugation of the metallocarbonyl moiety to biomolecules and dendrimers [40].

The addition of aminophosphonate to the carbon–carbon double bond was confirmed by the disappearance of ethylenic protons at 6.7 ppm characteristic for the maleimide ligand of half-sandwich complexes **1**–**2** along with the appearance of complex and appropriate signals in the ^1^H NMR spectra attributed to the 3 protons of the succinimide CH-CH_2_ motif (Figure S55). The splitting of the signal was compatible with an ABX spin system which was consistent with the formation of a stereogenic center. This observation is in line with our previously published works on aza-Michael additions [39,41].

The ^31^P NMR spectra were also recorded for all the compounds. The (aminomethyl)benzylphosphonate compounds exhibited a singlet at 26.6 ppm (**8a**-*ortho*), 26.6 ppm (**8b**-*meta*), and 26.5 ppm (**8c**-*para*), respectively, which was compatible with a phosphonate group connected to a *C_SP3_* carbon atom. ^31^P NMR signals for iron complexes **9a**–**c** were unchanged and stayed at 27.0 ppm, 26.3 ppm, and 26.4 ppm, respectively, and for ruthenium complexes **10a**–**c** at 26.8 ppm, 26.3 ppm, and **10c** 26.3 ppm, respectively. In both types of metal complexes for *ortho* derivatives, the ^31^P signal has been shifted downfield compared to the starting (aminomethyl)benzylphosphonate **8a**. This was possibly due to the deshielding of phosphoryl group (P=O), occurring as a result of an intramolecular H-bond between NH and O=P, forming a stable 7-membered ring, whereas this type of H-bonding was missing in the other complexes.

The IR spectrum of these complexes displayed two intense *ν*(C≡O) bands at 2045 and 1994 cm^−1^ for the CpFe(CO)_2_ moiety in iron complexes or at 2045 and 1990 cm^−1^ for the CpRu(CO)_2_ moiety for ruthenium complexes and characteristic bands for phosphonate absorption around 1250, 1025, and 965 cm^−1^ (Figure S56) [32]. The complexes were soluble in organic solvents and in water. Electrospray ionization mass spectra (ESI-MS) and elemental analysis confirmed the structures of iron (**9a**–**c**) and ruthenium (**10a**–**c**) complexes.

### 2.4. Biological Studies

#### 2.4.1. Cholinesterase Inhibitory Activities

Obtained aminophosphonates were evaluated in vitro for their inhibitory activity for the electric eel AChE (eeAChE) using a modified Ellman assay [42]. Furthermore, the activities of **8a**–**c** to **10a**–**c** towards BuChE (from equine serum) were also determined. Most of the compounds tested showed IC_50_ greater than 1 mM. Compared to tacrine (positive control), the new compounds showed lower inhibitory activity for AChE and BuChE. With respect to BuChE inhibitory activities, the ruthenium complexes **10a** and **10c** were the most active inhibitors (respectively, IC_50_ = 186 and 292 µM). Concerning iron complexes, only the *ortho* derivative **9a** showed some activity (IC_50_ = 331 µM) towards BuChE (Table 1).

These results are consistent with previously published work in which the metallocarbonyl iron complex **5a** showed a similar inhibitory activity toward BuChE (IC_50_ = 220 μM).

The inhibitory tests against eeAChE showed that the ruthenium complex **10a** was the only one of the metal complexes with some inhibitory potential (IC_50_ = 164 μM).

Interestingly, the new *ortho*-substituted aminophosphonate **8a** was found to be the most active of all tested compounds with an IC_50_ value of 1.215 µM towards AChE. This result corroborates with our theoretical prediction, presented later, and is in good agreement with some recently reported activities of different aminophosphonates on the cholinergic system [43,44,45]. For example, a chromone-derived aminophosphonate acts as an acetylcholinesterase inhibitor with IC_50_ = 0.103 µM. The docking studies of *N*-substituted α-aminophosphonate bearing a chromone moiety reveal that it binds to the peripheral anionic site and catalytic anionic site of AChE. The protonated amino group present in this compound is responsible for a cation–π interaction with TYR334 and H-bonding interaction with ASP72 [43].

Further studies to get inside into the mechanism of binding of *ortho*-substituted aminophosphonate **8a** to AChE should be performed.

#### 2.4.2. Cell Culture and MTT Cytotoxicity Test

**Figure 5:**
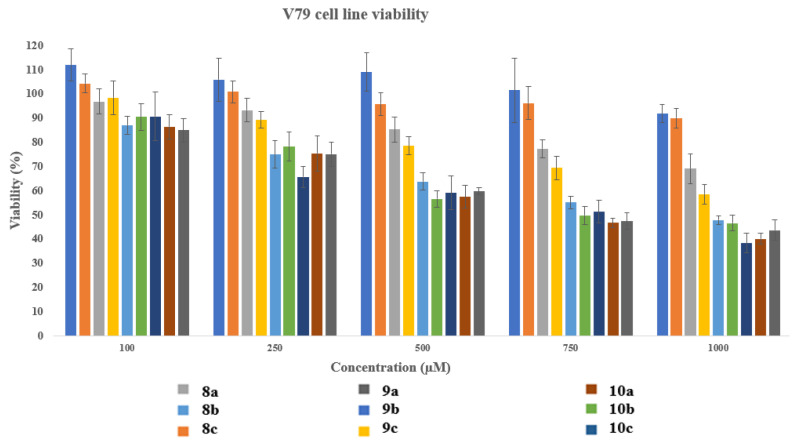
Percentage of cell viability after 24 h incubation with compounds as function of the concentration with phosphonates **8a**–**c**, **9a**–**c**, **10a**–**c**.

The benzylphosphonates derivatives **8a**–**c**, the corresponding iron **9a**–**c**, and ruthenium **10a**–**c** complexes were tested for their potential cytotoxic effect on the V79 cell line, which has been widely used to study the toxicity, mutagenicity, and repair of a wide variety of DNA damaging agents. Due to the solubility issues and toxicity of DMSO (used to prepare the stock solutions of compounds), at a concentration higher than >0.2%, compounds were tested in the concentration range of 100–1000 µM. Cell viability below 70% indicates cytotoxicity. Compounds **8a**–**c** did not exhibit cytotoxic activity in the tested range of concentrations (cell viability > 70%). Relative positions of phosphonomethyl and aminomethyl groups did not affect the cytotoxicity. Iron complex **9c** was slightly cytotoxic at concentrations of 1000 µM and 750 µM, with viabilities of 58% and 69%, respectively (cell viability below 70%). Compounds **9a**–**b** and **10a**–**c** were slightly cytotoxic with IC_50_ values of 690 µM, 909 µM, 725 µM, 787 µM, and 763 µM (Table 2, Figure 5). Compounds **9a** and **10a** had phosphonate groups in the *ortho* position, which turned out to induce the strongest toxicity.

#### 2.4.3. Theoretical Prediction Analysis of Ligands **8a**–**c** with AChE

To confirm the experimental results obtained from the cholinesterase assay, we analyzed the results of the theoretical prediction of ligand–protein interactions. Although the most interesting would be to see the effect of these new synthetic compounds on the human AChE (hAChE), the most relevant here is to compare it to the same enzyme, electric eel AChE (eeAChE). The crystal structure of the electric eel acetylcholinesterase (eeAChE) (PDB ID: 1C2O) was completed using modeling and aligned with the structure of human hAChE (PDB ID: 1B41) in order to compare the structural similarity (Figure S59) and the observed RMSD is less than 1.0A. Furthermore, after optimization using Gaussian-163, the structure of eeAChE was used for molecular docking by AutoDock 4 (ADT) with further refinement using the AutoDock tool. During the simulation, the ligands were used flexibly, and root-mean-square deviation (RMSD) values less than 1.0A were considered optimal for clustering. In the results, the ligand with the highest binding energy (most negative) was observed to have the highest binding affinity (Table 3).

**Figure 6:**
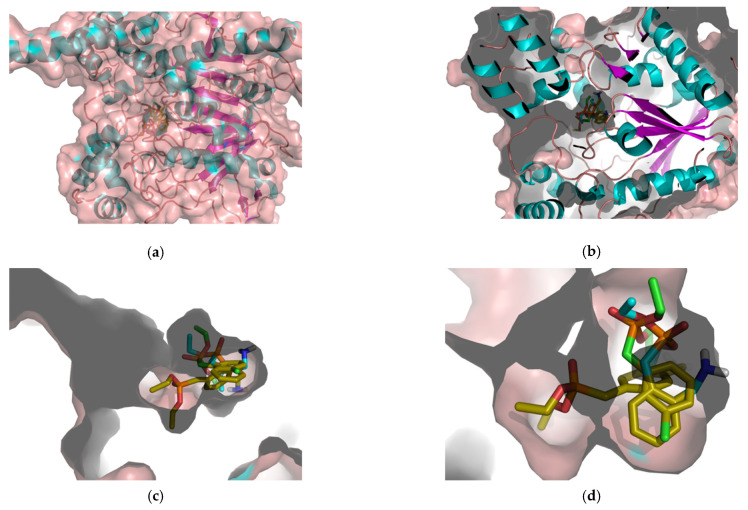
(**a**–**d**) The structure of electric eel AChE with the three ligands **8a**–**c**. (**a**) The overall structure of the protein with secondary structure projection and protein surface; (**b**) the protein projection with all three compounds, **8a**–**c**; (**c**,**d**) the active side of the eeAChE with three ligands.

The overall structure of the protein with all three ligands is presented in Figure 6a; it is a relatively compact protein with two extended α-helixes (Figure S62). Each of the **8a**–**c** compounds was positioned and fitted nicely in the active center (Figure 6b and Figure S60), which is a small pocket led by the tight canal (Figure 6c). All three compounds are in the active center; however, the conformation of each is different (Figure 6c,d and Figures S64–S66). When comparing the docking energy of the eeAChE-interacted ligand complexes, the **8a**-interacted eeAChE complex has the highest docking energy of −7.80 kcal/mol. The maximum and minimum occupancy of hydrogen bonds was found to be 5 and 2, respectively. The **8a**–eeAChE complex has the highest number of hydrogen bond formations of 5 compared to the other ligands as shown in Figure S63. These results corroborate well with the experimental data, which states that ligand **8a** has a strong interaction with the active site residues of eeAChE.

## 3. Materials and Methods

### 3.1. Materials

All the reactions were carried out under a nitrogen or argon atmosphere using standard Schlenk techniques. All solvents were distilled over standard drying agents directly before use. The starting materials were purchased from commercial suppliers (Sigma-Aldrich (Darmstadt, Germany), Merck (Darmstadt, Germany), Alfa Aesar (Kandel, Germany), Trichemicals (Newark, U.S)). All reagents were used as received unless otherwise stated. Acetonitrile was freshly distilled and stored over molecular sieves. Chromatographic purifications were performed on Silica gel Merck 60 (230–400 mesh). Complexes (η^5^-C_5_H_5_)M(CO)*_2_*(η^1^-*N*-maleimidato) (M = Fe, Ru) **1**–**2** were synthesized as previously described [23,24].

### 3.2. General Methods

IR spectra were recorded using a NICOLET iS50 instrument equipped with a liquid N_2_-cooled mercury-cadmium-telluride (MCT-A) detector. A flat crystal plate Zn-Se ATR unit (crystal thickness 4 mm) was used. The incidence angle was 45°. The ATR accessory was obtained from Pike Technologies.

^1^H NMR, ^13^C NMR, and ^31^P{H}NMR spectra were recorded on BRUKER Ultra Shield 400 Plus (400 MHz), BRUKER Avance III (600 MHz), and Varian Gemini (200 MHz) spectrometers, unless otherwise noted. Measurements were made in CDCl_3_ or DMSO-d_6_ solutions. Coupling constants were expressed in Hertz (Hz). ^31^P NMR spectra were obtained with the use of broadband ^1^H decoupling. Copies of IR and NMR spectra are presented at Supplementary Materials Figures S1–S54. Electrospray ionization mass spectra were performed on a Varian 500-MS LC ion trap (University of Lodz). Elemental analysis was performed on a Micro Vario Cube elemental analyzer (Elementar).

### 3.3. Inhibition of AChE and BuChE

The method of Ellman et al. [42] with modifications was followed. Nine different concentrations of each compound were used in order to measure inhibition of AChE or BuChE activity. The assay solution consisted of a 0.1 M phosphate buffer pH 8.0, with the addition of 0.4 mg/mL 5,5′-dithio-bis(2-nitrobenzoic acid), 2 unit/mL of EeAChE or 4 unit/mL of EqBuChE (Sigma Aldrich, Darmstadt, Germany), and 1 mM or 2 mM AChE or BuChE substrate (acetylthiocholine iodide or butyrylthiocholine iodide), respectively. Assays were performed with a blank containing all components except AChE or BuChE. The reaction rates were compared and the percentage of inhibition due to the presence of the test compound was calculated. Each concentration was analyzed in triplicate and repeated three times, and IC_50_ values were determined graphically from log concentration–inhibition curves.

### 3.4. Cell Culture and MTT Cytotoxicity Test

V79 cells (*Cricetulus griseus* lung fibroblast cell line) (European Collection of Cell Culture) were grown in MEM (PAN-Biotech, Aidenbach, Germany) containing 2 mM glutamine (Sigma Aldrich, Darmstadt, Germany)), 1% non-essential amino acids (Biological Industries, Cromwell, U.S), 10% FBS (Biowest, Nuaillé, France), and 100 units/mL penicillin and 100 mg/mL streptomycin (Biological Industries, Cromwell, U.S). Cells were plated and grown in an incubator at 37 °C with 5% CO_2_ to 80% confluence before the initiation of the assay.

V79 cells were seeded at a density of 1 × 10^4^ cells per well and were incubated for 24 h. After 24 h, the medium was removed from the wells, and cells were exposed to the compound solutions over a range of concentrations or nothing but culture medium (blank control). Cells were further incubated for an additional 24 h. At the end, the medium was removed and MTT solution (0.75 mg/mL) was added. Next, plates were incubated in the dark for 2 h at 37 °C. Then, the MTT solution was removed and DMSO was added to each well. Plates were incubated for 10 min at room temperature. The absorbance at 570 nm was measured on a microplate reader. Cell viability was expressed as a percentage of the control values (blank).

### 3.5. General Procedures for the Synthesis of ***6a***–***c***

Triethylphosphite (1 equiv) was dropwise added to α,α’-dibromo or dichloro-*I*-xylene (*I* = *o*, *m*, *p*) to a two-necked flask fitted with a Dean–Stark separator to remove bromoethane or chloroethane. The reaction mixture was heated to 110 °C for 6 h and then cooled to rt. The product was purified by column chromatography on silica gel (ethyl acetate to ethyl acetate/MeOH 80:20) to give diethyl (bromo or chloromethyl)benzylphosphonates (**6a**–**c**) in yields ranging from 71% to 84%. Details-Supplementary Data (Table S1).

### 3.6. General Procedures for the Synthesis of ***7a***–***c***

A mixture of bromomethyl or chloromethylbenzylphosphonate **6a**–**c** (1 equiv), phthalimide (1.2 equiv), and NaH (60% in mineral oil, 1.2 equiv) in dry acetonitrile was heated to 110 °C for 8 h. When the solution was cooled to rt, the solvent was removed under reduced pressure, and the crude product was purified by column chromatography on silica gel (ethyl acetate to ethyl acetate/MeOH 40:60). The diethyl (dioxoisoidolin-2-yl)methyl)benzyl-phosphonates were obtained (**7a**–**c**) in yields ranging from 74% to 93%. Details-Supplementary Data (Table S2).

### 3.7. General Procedures for the Synthesis of Diethyl(aminomethyl)benzylphosphonates ***8a***–***c***

A mixture of diethyl(1,3-dioxoisoidolin-2-yl)methylbenzylphosphonate (**7a**–**c**) and hydrazine monohydrate in EtOH was stirred at reflux for 3 h. After the mixture was cooled to 25 °C, the resulting precipitate was filtered and then washed 3 times with toluene. The filtrate was concentrated under reduced pressure, and the crude product was purified by column chromatography on silica gel (ethyl acetate to ethyl acetate/MeOH 90:10) to give pure diethyl (aminomethyl) benzylphosphonates (**8a**–**c**) in yields ranging from 69% to 93%. Details-Supplementary Data (Table S3).

### 3.8. Reactions of Complexes ***1***–***2*** with (Aminomethyl)benzylphosphonates ***8a***–***c***

To an argon-saturated solution of metallocarbonyl complex CpM(CO)_2_(η^1^-*N*-maleimidato) (M = Fe **1**, Ru **2**) (1 equiv) and diethyl(aminomethyl)benzylphosphonate **8a**–**c** (1.5 equiv) in a mixture of EtOH and distilled water (9:1), K_2_CO_3_ (5 equiv) was added. The reaction mixture was stirred for 24 h at rt. The mixture was extracted with CHCl_3_. The CHCl_3_ extracts were concentrated in vacuo. The residue was purified by column chromatography on silica gel (CHCl_3_ to CHCl_3_/MeOH 90:10) to give **9a**–**c** and **10a**–**c** metallocarbonyl complexes in moderate to good yields (40–77%).

### 3.9. Crystallographic Data

Single crystals of **7a** and **7c** were obtained after slow evaporation of dichloromethane solution at 5 °C. Suitable crystals were sequentially mounted on a fiber loop and used for X-ray diffraction measurement. X-ray data were collected on an Oxford Diffraction SuperNova DualSource diffractometer with the use of the monochromated CuKα X-ray source (λ = 1.54184). The crystals were kept at 100 K during data collection. Data reduction and analytical absorption correction were performed with CrysAlis PRO [46]. Using Olex2 [47], the structures were solved with the ShelXS [48] structure solution program using direct methods and refined with the ShelXL [48] refinement package using least squares minimization.

The non-hydrogen atoms were refined anisotropically. Hydrogen atoms were introduced in calculated positions with idealized geometry and constrained using a rigid body model with isotropic displacement parameters equal to 1.2 of the equivalent displacement parameter of the parent atoms. A summary of relevant crystallographic data is given in Table S4 in Supplementary Data. The geometry of hydrogen bonds was calculated by Mercury 4.10 [49] and gathered in Table S5 in Supplementary Data along with the symmetry codes of the described intermolecular non-covalent interactions.

### 3.10. Molecular Docking

The crystal structure of the electric eel acetylcholinesterase (eeAChE) was downloaded from the RCSB protein data bank (PDB ID: 1C2O) [50]. To complete the whole protein structure, the sequence was supplemented and modeled using the blastp suite [51]. The geometry of the complete modeled eeAChE protein was compared to the X-ray structure of eeAChE (PDB ID: 1C2O) in order to confirm the methods utilized in this study, and the observed RMSD were less than 1A. Furthermore, optimization was carried out using Gaussian-16 [52] software with the WB97XD [53] level of DFT (density functional theory)/LANL2DZ [54] level of theory to minimize steric clashes in ligand structures. To find the stability of the structure, harmonic vibrational frequency analyses were carried out at the same computational level of theory. The chemical shift calculations for NMR were performed using the gauge-independent atomic orbital (GIAO) [55] method at the WB97XD LANL2DZ. All of the stable structures were found to be at energy minima with the lowest possible positive frequencies. Initially, the presence of solvent molecules and ligands was cleared by AutoDock 4 (ADT), which is mainly used to assign charge to the protein and ligand complexes prior to analysis or docking. The molecular docking of the protein molecule was further refined using the AutoDock tool. The ligands were considered to be flexible throughout the docking process, while the protein was considered to be rigid. Positional root-mean-square deviation (RMSD) values less than 1.0A were considered optimal and clustered together to identify the best binding. The ligand with the highest binding energy (most negative) was observed to have the highest binding affinity. The data of the top 5 complexes are listed in Supplementary Table S6. The docking predicted binding conformations for the ligands are shown in Figure 6a–d and their corresponding results are presented in Table 3. The computer-generated 3D structure of selected ligands **8a–c** are presented at Figure S61.

The complete data set for the crystal structures under study has been deposited at the Cambridge Crystallographic Data Centre (CCDC 2154277 and CCDC 2154278). 

## 4. Conclusions

In conclusion, we report the design, synthesis, and characterization of (aminomethyl)benzylphosphonates **8a**–**c** and their metallocarbonyl complexes **9a**–**c** (Fe) and **10a**–**c** (Ru) obtained from phosphonates **6a**–**c** and **7a**–**c**. All obtained compounds were characterized by spectroscopic methods (^1^H-, ^13^C-, ^31^P{^1^H}-NMR, IR) and mass spectrometry. The structures of compounds **7a** and **7c** were also confirmed by single-crystal X-ray diffraction analysis.

The (aminomethyl)benzylphosphonates **8a**–**c** and their metallocarbonyl complexes **9a**–**c** (Fe) and **10a**–**c** (Ru) were tested for inhibitory potentials against acetylcholinesterase (AChE) and butyrylcholinesterase (BuChE) by determination of IC_50._ Inhibitory studies showed that the best inhibitor of the studied compounds was the *ortho*-substituted (aminomethyl)benzylphosphonate **8a** with IC_50_ = 1.215 µM against AChE. The docking studies performed for complexes **8a**–**c** reveal that the **8a**–eeAChE complex has the highest number of hydrogen bond formations, which is well correlated with experimental results.

The IC_50_ values of metallocarbonyl complexes **9a**–**c** (Fe) and **10a**–**c** (Ru) are much higher compared to the reference inhibitor (tacrine); however, complexes **9a**, **10a**, and **10c** show inhibitory activities towards BuChE similar to that of the previously reported iron metallocarbonyl complex **5b** [32].

Cytotoxicity studies towards the V79 cell line show no cytotoxicity of the phosphonates **8a**–**c**, and some cytotoxicity for the metallocarbonyl complexes **9a**–**c** and **10a**–**c**. The most cytotoxic were the *ortho*-derivatives **9a** and **10a**.

This study has presented a novel approach to accessing some (aminomethyl)benzylphosphonates and their metallocarbonyl derivatives. Our findings are in good agreement with the results of other research teams on aminophosphonates [43,44,45]. They clearly indicate that the aminophosphonates provide a good basis for the development of an effective substance that inhibits the cholinergic system. The (aminomethyl)benzylphosphonate **8a** should be further carefully examined in terms of the inhibition mechanism of AChE. In our previous work, we found that the half-sandwich metallocarbonylphosphonic acids showed an inhibiting activity against certain serine hydrolases [32]. Therefore, we plan to synthesize the corresponding phosphonic acid of compounds **8a**–**c**, **9a**–**c**, and **10a**–**c** and test their inhibitory activity towards AChE and BuChE.

## Figures and Tables

**Table 1:** Inhibitory effects of compounds **8a**–**c**, **9a**–**c**, **10a**–**c** against AChE and BuChE.

Metal	Compound	IC_50_ AChE ± SD (µM) ^a^	IC_50_ BuChE ± SD (µM) ^b^
-	**8a**	1.215 ± 0.006	>1000
-	**8b**	>1000	>1000
-	**8c**	>1000	363 ± 29
Fe	**9a**	>1000	331 ± 41
Fe	**9b**	>1000	>1000
Fe	**9c**	>1000	>1000
Ru	**10a**	>1000	186 ± 21
Ru	**10b**	>1000	389 ± 26
Ru	**10c**	164 ± 21	292 ± 31
-	Tacrine	0.056 ± 0.005	0.018 ± 0.002

^a^ IC_50_: 50% inhibitory concentration (means ± SD of three independent experiments) AChE. ^b^ IC_50_: 50% inhibitory concentration (means ± SD of three independent experiments) BuChE.

**Table 2:** Viability of the V79 cell line after treatment with compounds.

Compound	9a	9b	9c	10a	10b	10c	8a	8b	8c
Cytotoxicity	IC_50_ = 690 ± 42 µM	IC_50_ = 909 ± 53 µM	Toxic at concentrations of 750 and 1000 µM—viability of 69% and 58%, respectively	IC_50_ = 725 ± 62 µM	IC_50_ = 787 ± 113 µM	IC_50_ = 763 ± 77 µM	Non-toxic		

**Table 3:** Molecular docking analysis of AChE and ligand complexes.

Ligand	Binding Energy Kcal/mol	Intermolecular Energy Kcal/mol	Total Internal Energy Kcal/mol	Torsional Free Energy Kcal/mol	Unbound Systems Energy Kcal/mol
**8a**	−7.80	−10.18	−1.90	2.39	−1.90
**8b**	−6.36	−8.74	−0.64	2.39	−0.64
**8c**	−6.74	−9.13	0	2.39	0

## Data Availability

Not applicable.

## Supplementary Materials

The following supporting information can be downloaded at: https://www.mdpi.com/article/10.3390/ijms23158091/s1.
