# Supplementary material for: Synthesis and Biological Studies of Novel Aminophosphonates and Their Metal Carbonyl Complexes (Fe, Ru)
Source: Int J Mol Sci. 2022 Jul 22;23(15):8091. doi: 10.3390/ijms23158091 (PMC9330042; doi:10.3390/ijms23158091)
Supplement: Supplementary file 1 [file ijms-23-08091-s001.zip › ijms-1787659-supplementary.pdf]

Synthesis and biological studies of novel aminophosphonates and their metal carbonyl complexes (Fe, Ru)

Aneta Kosińska[1,2*], David Virieux[2], Jean-Luc Pirat[2], Kamila Czarnecka[3], Małgorzata Girek[4], Paweł Szymański[3,5], Sławomir Wojtulewski[6], Saranya Vasudevan[7], Arkadiusz Chworos[7], Bogna Rudolf[1*]

[1] *Faculty of Chemistry, Department of Organic Chemistry, University of Lodz, Tamka 12, 91-403 Lodz, Poland*
[2] *ICGM, University of Montpellier, CNRS, ENSCM, 240 Av. du Professeur Émile Jeanbrau, 34090 Montpellier, France*
[3] *Department of Pharmaceutical Chemistry, Drug Analyses and Radiopharmacy, Faculty of Pharmacy, Medical University of Lodz, Muszynskiego 1, 90-151 Lodz, Poland*
[4] *Animal House, Faculty of Pharmacy, Medical University of Lodz, Muszynskiego 1, 90-151 Lodz, Poland*
[5] *Department of Radiobiology and Radiation Protection, Military Institute of Hygiene and Epidemiology, Kozielska 4, 01-163 Warsaw, Poland*
[6] *Department of Structural Chemistry, Faculty of Chemistry, University of Bialystok, Ciołkowskiego 1K, 15-245 Bialystok, Poland*
[7] *Centre of Molecular and Macromolecular Studies, Polish Academy of Sciences, Sienkiewicza 112, 90-363 Lodz, Poland*

Corresponding authors:
Aneta Kosińska aneta.kosinska@chemia.uni.lodz.pl
Bogna Rudolf bogna.rudolf@chemia.uni.lodz.pl

Supplementary Materials

1.1. Experimental data……………………………………………………………………………………..2

1.2. Spectra and spectral data……………………………………………………………………………..8

1.3. Crystallographic data………………………………………………………………………………. .35

1.4. Docking studies……………………………………………………………………………………….38

General procedures for the synthesis of ***6a-c***

Triethylphosphite – 1 equiv was dropwise added to *α,α'*-dibromo or dichloro-*I*-xylene (*I* = *o*, *m*, *p*) to a two-necked flask fitted with a Dean-Stark separator to remove bromoethane or chloroethane. The reaction mixture was heated to 110 °C for 6 h and then cooled to rt. The product was purified by column chromatography on silica gel (ethyl acetate to ethyl acetate/MeOH 80:20) to give diethyl(bromo or chloromethyl)benzylphosphonates (**6a-c**) in yields ranging from 71% to 84%.

Supplementary Table S1. Reagents and quantities used for the synthesis of **6a-c**.

α,α'-dibromo or dichloro-*I*-xylene		$P(OEt)_3$	Product	Yield
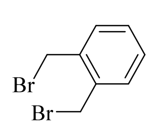	6.24 g, 23.64 mmol	2.00 ml, 11.66 mmol	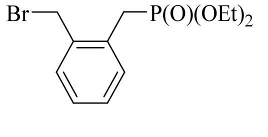 **6a**	**2.81 g, 75%**
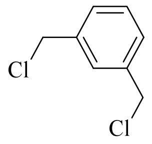	5.25 g, 29.99 mmol	2.60 ml, 15.16 mmol	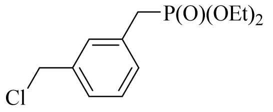 **6b**	**3.48 g, 84%**
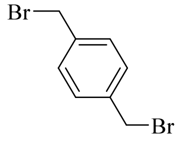	6.08 g, 23.03 mmol	1.97 ml, 11.49 mmol	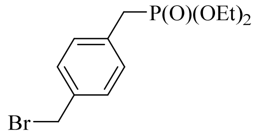 **6c**	**2.61 g, 71%**

6a: *α,α'*-Dibromo-*o*-xylene (6.24 g, 23.64 mmol), triethyl phosphite (2.00 mL, 11.66 mmol). **Yield:** 2.81 g, 75%, yellow oil. **^{31}P{H}NMR** (243MHz, $CDCl_3$): δ 25.4 ppm. **^{1}H NMR** (600MHz, $CDCl_3$): δ 7.34 (d, *J* = 7.5 Hz, 1H, CHar), 7.30–7.21 (m, 3H, CHar) 4.70 (s, 2H, CH_2Br), 4.07–3.96 (m, 4H, OCH_2), 3.34 (d, *J* = 21.8 Hz, 2H, CH_2P), 1.24 (t, *J* = 7.1 Hz, 6H, CH_3) ppm. **^{13}C NMR** (151 MHz, $CDCl_3$): δ 136.63 (d, *J* = 6.3 Hz, Car), 131.74 (d, *J* = 5.7 Hz, Car), 131.05 (d, *J* = 9.4 Hz, Car), 130.77 (d, *J* = 3.3 Hz, Car), 128.99 (d, *J* = 3.6 Hz, Car), 127.66 (d, *J* = 3.8 Hz, Car), 62.29 (d, *J* = 6.8 Hz, OCH_2), 32.35 (s, CH_2-Br), 30.86 (d, *J* = 137.9 Hz,, CH_2-P), 16.35 (d, *J* = 6.0 Hz, CH_3) ppm. **ESI-MS** m/z calcd for $C_{12}H_{18}BrO_3P$ $(M+H)^+$: 321.02; found: 321.10.

6b: *α,α'*-Dichloro-*m*-xylene (5.25 g, 29.99 mmol), triethyl phosphite (2.60 ml, 15.16 mmol). **Yield:** 3.48 g, 84%, pale yellow oil. **^{31}P{H}NMR** (162 MHz, $CDCl_3$): δ 26.0 ppm. **^{1}H NMR** (400 MHz, $CDCl_3$): δ 7.32–7.22 (m, 4H, CHar), 4.56 (s, 2H, CH_2Cl), 4.04–3.95 (m, 4H, OCH_2), 3.14 (d, *J* = 21.7 Hz, 2H, CH_2P), 1.24 (dt, *J* = 0.4, 7.1 Hz, 6H, CH_3) ppm. **^{13}C NMR** (151 MHz, $CDCl_3$): δ 137.74 (d, *J* = 3.1 Hz, Car), 132.31 (d, *J* = 9.0 Hz, Car), 129.97 (d, *J* = 6.6 Hz, Car), 129.81 (d, *J* = 6.5 Hz, Car), 128.89 (d, *J* = 3.0 Hz, Car), 127.08 (d, *J* = 3.5 Hz, Car), 62.17 (d, *J* = 6.8 Hz, OCH_2), 46.00 (s, Cl-CH_2-ar), 33.64 (d, *J* = 138.3 Hz, CH_2-P), 16.34 (d, *J* = 6.0 Hz, CH_3) ppm. **ESI-MS** m/z calcd for $C_{12}H_{18}ClO_3P$ $(M+H)^+$: 277.07; found: 277.10.

6c: *α,α'*-Dibromo-*p*-xylene (6.08 g, 23.03 mmol), triethyl phosphite (1.97 ml, 11.49 mmol). **Yield:** 2.61 g, 71%, pale yellow oil. **^{31}P{H}NMR** (162 MHz, $CDCl_3$): δ 25.9 ppm. **^{1}H NMR** (400 MHz, $CDCl_3$) δ 7.33 (d, *J* = 8.1 Hz, 2H, CHar), 7.27 (d, *J* = 2.4 Hz, 1H, CHar), 7.25 (d, *J* = 2.5 Hz, 1H, CHar), 4.47 (d, *J* = 0.9 Hz, 2H, CH_2Br), 4.07–3.96 (m, 4H, OCH_2), 3.13 (d, *J* = 21.8 Hz, 2H, CH_2-P), 1.26–1.20 (m, 6H, CH_3) ppm. **^{13}C NMR** (151 MHz, $CDCl_3$): δ 136.44 (d, *J* = 3.8 Hz, Car), 132.06 (d, *J* = 9.1 Hz, Car), 130.17 (d, *J* = 6.6 Hz, 2Car), 129.23 (d, *J* = 3.0 Hz, 2Car), 62.19 (d, *J* = 6.7 Hz, OCH_2), 34.02 (s, CH_2Br), 33.56 (d, *J* = 138.2 Hz,

CH_2P), 16.36 (d, *J* = 5.9 Hz, CH_3) ppm. **ESI-MS** m/z calcd for $C_{12}H_{18}BrO_3P$ $(M+H)^+$: 321.02; found: 321.10.

General procedures for the synthesis of ***7a-c***

The mixture of bromomethyl or chloromethylbenzylphosphonate **6a-c** (1 equiv), phthalimide (1.2 equiv) and NaH (60% in mineral oil, 1.2 equiv) in dry acetonitrile was heated to 110 °C for 8h. When the solution has been cooled to rt, the solvent was removed under reduced pressure, and the crude product was purified by column chromatography on silica gel (ethyl acetate to ethyl acetate/MeOH 40:60). The diethyl(dioxoisoidolin-2-yl)methyl)benzyl-phosphonates were obtained (**7a-c**) in yields ranging from 74% to 93%.

Supplementary Table S2. Reagents and quantities used for the synthesis of **7a-c**.

(Bromo/chloromethyl)benzylphosphonate		Phthalimide/NaH/CH_3CN	Product	Yield
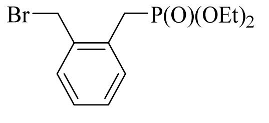 **6a**	1.70 g, 5.29 mmol	0.93 g, 6.32 mmol/ 0.25 g, 6.32 mmol/ 25 ml	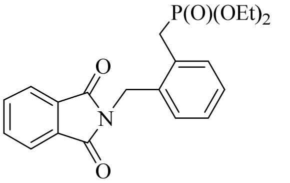 **7a**	1.87 g, 91%
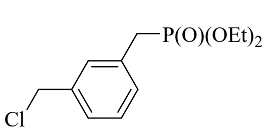 **6b**	1.84 g, 6.65 mmol	1.18 g, 8.00 mmol/ 0.32 g, 8.00 mmol/ 30 ml	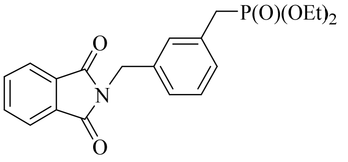 **7b**	1.92 g, 74%
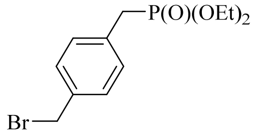 **6c**	1.70 g, 5.29 mmol	0.93 g, 6.32 mmol/ 0.25 g, 6.32 mmol/ 25 ml	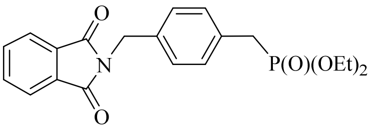 **7c**	1.91 g, 93%

7a: Phthalimide (0.93 g, 6.32 mmol), NaH (0.25 g, 6.32 mmol), diethyl 2-(bromomethyl)benzylphosphonate **6a** (1.70 g, 5.29 mmol), 25 mL dry acetonitrile. **Yield:** 1.87 g, 91%, pale yellow oil, crystallize to pale yellow solid. ^{31}**P{H}NMR** (162 MHz, $CDCl_3$): δ 26.7 ppm. ^{1}**H NMR** (600 MHz, $CDCl_3$) δ 7.83 (dd, *J* = 5.4, 3.1 Hz, 2H, Char), 7.70 (dd, *J* = 5.4, 3.0 Hz, 2H, Char), 7.44 (d, *J* = 7.1 Hz, 1H, Char), 7.28 (d, *J* = 7.2 Hz, 1H, Char), 7.24–7.17 (m, 2H, Char), 4.99 (s, 2H, CH_2N), 4.06–3.98 (m, 4H, OCH_2), 3.57 (d, *J* = 21.8 Hz, 2H, CH_2P), 1.24 (t, *J* = 7.1 Hz, 6H, CH_3) ppm. ^{13}**C NMR** (151 MHz, $CDCl_3$) δ 168.23 (s, 2CO-N), 135.23 (d, *J* = 6.4 Hz, Car), 134.00 (s, 2Car), 132.14 (s, 2Car), 131.41 (d, *J* = 5.7 Hz, Car), 130.45 (d, *J* = 9.5 Hz, Car), 130.21 (d, *J* = 3.3 Hz, Car), 128.01 (d, *J* = 3.4 Hz, Car), 127.42 (d, *J* = 3.8 Hz, Car), 123.30 (s, 2Car), 62.19 (d, *J* = 6.8 Hz, OCH_2), 39.09 (s, CH_2N), 30.96 (d, *J* = 137.3 Hz,.CH_2P), 16.37 (d, *J* = 5.9 Hz, CH_3) ppm. **ESI-MS** m/z calcd for $C_{20}H_{22}NO_5P$ $(M+H)^+$: 388.12; found: 388.13.

7b: Phthalimide (1.18 g, 8.00 mmol), NaH (0.32 g, 8.00 mmol), diethyl 3-(chloromethyl)benzylphosphonate **6b** (1.84 g, 6.65 mmol), 30 mL dry acetonitrile. **Yield:** 1.92 g, 74%, pale yellow solid. ^{31}**P{H}NMR** (162 MHz, $CDCl_3$): δ 26.0 ppm. ^{1}**H NMR** (400 MHz, $CDCl_3$): δ 7.84 (dd, *J* = 5.5, 3.0 Hz, 2H, Char), 7.71 (*J* = 5.5, 3.0 Hz, 2H, Char), 7.34-7.27 (m, 3H, Char), 7.25-7.19 (m, 1H, Char), 4.83 (s, 2H, CH_2N), 4.07-3.92 (m, 4H, OCH_2), 3.12 (d, *J* = 21.7 Hz, 2H, CH_2P), 1.21 (dt, *J* = 0.4, 7.1 Hz, 6H,

CH_3) ppm. **^{13}C NMR** (151 MHz, $CDCl_3$): δ 167.93 (s, 2CO-N), 136.63 (d, *J* = 3.0 Hz, Car), 133.99 (s, Car), 132.25 (s, Car), 132.19 (s, Car), 132.12 (s, Car), 129.85 (d, *J* = 6.8 Hz, Car), 129.28 (d, *J* = 6.3 Hz, Car), 128.89 (d, *J* = 3.1 Hz, Car), 127.07 (d, *J* = 3.6 Hz, Car), 123.33 (s, Car), 62.12 (d, *J* = 6.8 Hz, OCH_2), 41.45 (s, CH_2-N), 33.68 (d, *J* = 138.0 Hz,CH_2P), 16.30 (d, *J* = 6.0 Hz, CH_3) ppm. **ESI-MS** m/z calcd for $C_{20}H_{22}NO_5P$ $(M+H)^+$: 388.12; found: 388.13.

7c: Phthalimide (0.93 g, 6.32 mmol), NaH (0.25 g, 6.32 mmol), diethyl 4-(bromomethyl)benzylphosphonate **6c** (1.70 g, 5.29 mmol), 25 mL dry acetonitrile. **Yield:** 1.91 g, 93%, white solid. **^{31}P{H}NMR** (162 MHz, $CDCl_3$): δ 26.1 ppm. **^{1}H NMR** (600 MHz, $CDCl_3$) δ 7.83 (dd, *J* = 5.4, 3.1 Hz, 2H, Char), 7.69 (dd, *J* = 5.5, 3.0 Hz, 2H, Char), 7.36 (d, *J* = 7.9 Hz, 2H, Char), 7.23 (dd, *J* = 8.2, 2.4 Hz, 2H, Char), 4.81 (s, 2H, CH_2N), 4.06–3.88 (m, 4H, OCH_2), 3.09 (d, *J* = 21.7 Hz, 2H, CH_2P), 1.22 (t, *J* = 7.1 Hz, 6H, CH_3) ppm. **^{13}C NMR** (151 MHz, $CDCl_3$) δ 167.99 (s, 2CO-N), 135.00 (d, *J* = 3.8 Hz, Car), 133.98 (s, 2Car), 132.14 (s, 2Car), 131.30 (d, *J* = 9.2 Hz, Car), 130.03 (d, *J* = 6.5 Hz, 2Car), 128.80 (d, *J* = 3.0 Hz, 2Car), 123.33 (s, 2Car), 62.12 (d, *J* = 6.8 Hz, OCH_2), 41.25 (s, CH_2N), 33.44 (d, *J* = 138.2 Hz, CH_2-P), 16.34 (d, *J* = 6.0 Hz, CH_3) ppm. **ESI-MS** m/z calcd for $C_{20}H_{22}NO_5P$ $(M+H)^+$: 388.12; found: 388.11.

General procedures for the synthesis of diethyl(aminomethyl)benzylphosphonates ***8a-c***

A mixture of diethyl(1,3-dioxoisoidolin-2-yl)methylbenzylphosphonate (**7a-c**) and hydrazine monohydrate in EtOH was stirred at reflux for 3 h. After the mixture was cooled to 25 °C, the resulting precipitate was filtered and then washed 3 times with toluene. The filtrate was concentrated under reduced pressure, and the crude product was purified by column chromatography on silica gel (ethyl acetate to ethyl acetate/MeOH 90:10) to give pure diethyl(aminomethyl)benzylphosphonates (**8a-c**) in yields ranging from 69% to 93%.

Supplementary Table S3. Reagents and quantities used for the synthesis of **8a-c**.

**Diethyl(1,3-dioxoiso-idolin-2-yl) methylbenzylphosphonate**		**Monohydrate hydrazine/ Ethanol**	**Product**	**Yield**
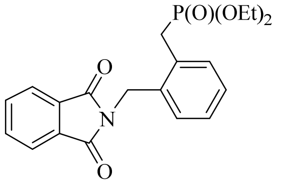 **7a**	1.57 g, 4.05 mmol	0.30 ml, 6.18 mmol/ 40 ml	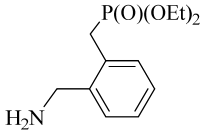 **8a**	0.72 g, 69%
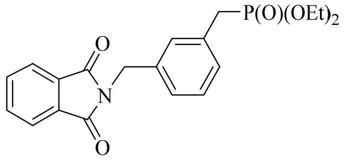 **7b**	1.60 g, 4.13 mmol	0.30 ml, 6.18 mmol/ 40 ml	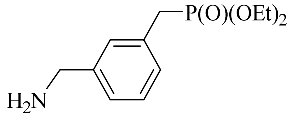 **8b**	0.91 g, 86%
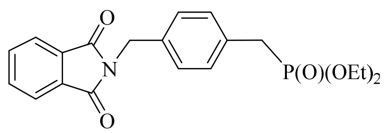 **7c**	1.60 g, 4.13 mmol	0.30 ml, 6.18 mmol/ 40 ml	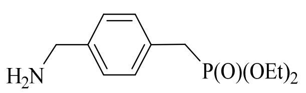 **8c**	0.99 g, 93%

8a: diethyl(2-((1,3-dioxoisoindolin-2-yl)methyl)benzyl)phosphonate; **7a** (1.57 g, 4.05 mmol), monohydrate hydrazine (0.30 ml, 6.18 mmol), 40 mL ethanol. **Yield:** 0.72 g, 69%, yellow oil. **^{31}P{H}NMR** (243 MHz, $CDCl_3$): δ 26.6 ppm. **^{1}H NMR** (600 MHz, $CDCl_3$): δ 7.36 (d, *J* = 7.4 Hz, 1H, CHar), 7.27 (d, *J* = 7.4 Hz, 1H), 7.25-7.19 (m, 2H, CHar), 4.07-3.96 (m, Hz, 4H, OCH_2), 3.95 (s, 2H, C$\underline{H_2}$N), 3.28 (d, *J* = 21.9 Hz, 2H, CH_2P), 2.03 (s, 2H, N$\underline{H_2}$), 1.24 (dt, *J* = 0.4, 7.1 Hz, 6H, CH_3) ppm. **^{13}C NMR** (151 MHz, $CDCl_3$): δ 141.31 (d, *J* = 6.4 Hz, Car), 131.22 (d, *J* = 5.5 Hz, Car), 129.46 (d, *J* = 9.2 Hz, Car), 129.00 (d, *J* = 3.4 Hz, Car), 127.53 (d, *J* = 3.9 Hz, Car), 127.11 (d, *J* = 3.5 Hz, Car), 62.23 (d, *J* = 6.8 Hz, OCH_2), 43.87 (s, CH_2N), 30.47 (d, *J* = 138.3 Hz, CH_2P), 16.34 (d, *J* = 6.0 Hz, CH_3) ppm. **IR** (ATR, cm^{-1}): 3363, 3285 (-NH_2), 1227 (P=O), 1049, 1021 (P-O-CH_2CH_3). **ESI-MS** m/z calcd for $C_{12}H_{20}NO_3P$ $(M+H)^+$: 258.12; found: 258.10.

8b: diethyl(3-((1,3-dioxoisoindolin-2-yl)methyl)benzyl)phosphonate; **7b** (1.60 g, 4.13 mmol), monohydrate hydrazine (0.30 ml, 6.18 mmol), 40 mL ethanol. **Yield:** 0.91 g, 86%, yellow oil. **^{31}P{H}NMR** (162 MHz, $CDCl_3$): δ 26.6 ppm. **^{1}H NMR** (400 MHz, $CDCl_3$): δ 7.30-7.23 (m, 2H, CHar), 7.22-7.13 (m, 2H, CHar), 4.07–3.90 (m, 4H, OCH_2), 3.84 (s, 2H, C$\underline{H_2}$-N), 3.14 (d, *J* = 21.7 Hz, 2H, CH_2P), 1.97 (s, 2H, N$\underline{H_2}$), 1.23 (dt, *J* = 0.4, 6.9 Hz, 6H, CH_3) ppm. **^{13}C NMR** (151 MHz, $CDCl_3$): δ 131.75 (d, *J* = 9.1 Hz, Car), 129.51 (dd, *J* = 29.1, 6.7 Hz, Car), 128.69 (d, *J* = 12.4 Hz, Car), 128.44 (d, *J* = 6.4 Hz, Car), 126.34 (s, Car), 62.21 (d, *J* = 6.8 Hz, OCH_2), 45.06 (s, CH_2N), 33.46 (d, *J* = 138.0 Hz, CH_2-P), 16.33 (d, *J* = 6.0 Hz, CH_3)ppm. **IR** (ATR, cm^{-1}): 3365, 3297 (-NH_2), 1247 (P=O), 1053, 1026 (P-O-CH_2CH_3). **ESI-MS** m/z calcd for $C_{12}H_{20}NO_3P$ $(M+H)^+$: 258.12; found: 258.14.

8c: diethyl(4-((1,3-dioxoisoindolin-2-yl)methyl)benzyl)phosphonate; **7c** (1.60 g, 4.13 mmol), monohydrate hydrazine (0.30 ml, 6.18 mmol), 40 mL ethanol. **Yield:** 0.99 g, 93%, yellow oil. **^{31}P{H}NMR** (243 MHz, $CDCl_3$): δ 26.5 ppm. **^{1}H NMR** (400 MHz, $CDCl_3$): δ 7.29 (d, *J* = 8.1 Hz, 2H), 7.26 (s, 1H), 7.25–7.22 (m, 1H), 4.09–3.89 (m, 4H, OCH_2), 3.86 (s, 2H, C$\underline{H_2}$N), 3.13 (d, *J* = 21.5 Hz, 2H, CH_2P), 2.28 (s, 2H, N$\underline{H_2}$), 1.25 (t, *J* = 7.1 Hz, 6H, CH_3) ppm. **^{13}C NMR** (151 MHz, $CDCl_3$): δ 141.42 (d, *J* = 3.8 Hz, Car), 130.14 (d, *J* = 9.2 Hz, Car), 129.91 (d, *J* = 6.6 Hz, 2Car), 127.38 (d, *J* = 3.1 Hz, 2Car), 62.11 (d, *J* = 6.7 Hz, OCH_2), 45.99 (s, CH_2N) 33.38 (d, *J* = 138.3 HzCH_2-P), 16.36 (d, *J* = 6.0 Hz, CH_3) ppm. **IR** (ATR, cm^{-1}): 3372, 3295 (-NH_2), 1227 (P=O), 1053, 1023 (P-O-CH_2CH_3). **ESI-MS** m/z calcd for $C_{12}H_{20}NO_3P$ $(M+H)^+$: 258.12; found: 257.11.

*Reactions of complexes **1**-**2** with (aminomethyl)benzylphosphonates **8a-c***

To argon saturated solution of metallocarbonyl complex $CpM(CO)_2(\eta^1$-*N*-maleimidato) (M = Fe **1**, Ru **2**) (1 equiv) and diethyl(aminomethyl)benzylphosphonate **8a-c** (1.5 equiv) in mixture of EtOH and distilled water (9:1), K_2CO_3 (5 equiv) was added. The reaction mixture was stirred 24 h at rt. The mixture was extracted with $CHCl_3$. The $CHCl_3$ extracts were concentrated *in vacuo*. The residue was purified by column chromatography on silica gel ($CHCl_3$ to $CHCl_3$/MeOH 90:10) to give **9a-c** and **10a-c** metallocarbonyl complex in moderate to good yields (40-77%).

9a: 1 (0.09 g, 0.33 mmol), diethyl-2-(aminomethyl)benzylphosphonate **8a** (0.13 g, 0.51 mmol), K_2CO_3 (0.24 g, 1.74 mmol), 9 mL ethanol, 1 mL H_2O. **Yield:** 109 mg, 60%, orange-brown oil. **^{31}P{H}NMR** (81 MHz, $CDCl_3$): δ 27.0. **^{1}H NMR** (200 MHz, $CDCl_3$) δ 7.36–7.27 (m, 1H, CHar), 7.24–7.17 (m, 3H, CHar), 5.04 (s, 5H, Cp), 4.07–3.94 (m, 4H, OCH_2), 3.89 (d, *J* = 13.2 Hz, 2H, C$\underline{H_2}$N), 3.68 (dd, *J* = 8.1, 5.1 Hz, 1H, $\underline{CH}$-CH_2), 3.33 (dd, *J* = 21.8, 6.8 Hz, 2H, P-$\underline{CH_2}$), 2.80 (dd, *J* = 17.4, 8.1 Hz, 1H, CH-$\underline{CH_2}$), 2.43 (dd, *J* = 17.4, 5.1 Hz, 1H, CH-$\underline{CH_2}$), 1.22 (dt, *J* = 0.4, 7.0 Hz, 6H, CH_3) ppm. **^{13}C NMR** (151 MHz, $CDCl_3$) δ 212.24 (d, *J* = 13.5 Hz, 2C≡O), 188.03 (s, C=O), 181.93 (s, C=O), 131.32 (d, *J* = 5.3 Hz, 2Car), 130.63 (s, Car), 130.57 (s, Car), 127.64 (s, Car), 127.33 (s, Car), 85.02 (s, Cp), 62.22 (dd, *J* = 7.0, 11.3 Hz, OCH_2), 59.28 (s, C-C), 49.87 (s, C-C), 39.11 (s, CH_2N), 30.45 (d, *J* = 137.4 Hz, CH_2P), 16.35 (d, *J* = 5.9 Hz, CH_3) ppm. Anal. Calcd for $C_{23}H_{27}FeN_2O_7P$: C, 52.09; H, 5.13; N, 5.28. Found: C, 52.07; H, 5.06; N, 5.27. **IR** (ATR, cm^{-1}): 2043, 1994 (C≡O), 1637 (CO imide), 1228 (P=O), 1051, 1026 (P-O-CH_2CH_3). **ESI-MS** m/z calcd for $C_{23}H_{27}FeN_2O_7P$ $(M+H)^+$: 531.10; found: 531.03.

9b: 1 (0.10 g, 0.37 mmol), diethyl-3-(aminomethyl)benzylphosphonate **8b** (0.14 g, 0.54 mmol), K_2CO_3 (0.25 g, 1.81 mmol), 9 ml ethanol, 1 ml H_2O. **Yield:** 95 mg, 49%, orange-brown oil. **^{31}P{H}NMR** (243

MHz, $CDCl_3$): δ 26.3 ppm. **^{1}H NMR** (600 MHz, $CDCl_3$) δ 7.28 (s, 1H, CHar), 7.25-7.21 (m, 3H, CHar), 5.04 (s, 5H, Cp), 4.04–3.99 (m, 4H, OCH_2), 3.80 (q, *J* = 13.1 Hz, 2H, N-C<u>H$_2$</u>-Ar), 3.62 (dd, *J* = 7.7, 5.0 Hz, 1H, <u>CH</u>-CH_2), 3.13 (d, *J* = 21.7 Hz, 2H, P-<u>CH$_2$</u>-Ar), 2.77 (dd, *J* = 17.3, 8.1 Hz, 1H, CH-<u>CH$_2$</u>), 2.46 (dd, *J* = 16.9, 4.0 Hz, 1H, CH-<u>CH$_2$</u>), 1.24 (t, *J* = 7.0 Hz, 6H, CH_3) ppm. **^{13}C NMR** (151 MHz, $CDCl_3$) δ 212.17 (d, *J* = 12.1 Hz, 2C≡O), 191.02 (s, C=O), 188.14 (s, C=O), 139.52 (s, Car), 131.88 (d, *J* = 9.1 Hz, Car), 129.79 (d, *J* = 6.6 Hz, Car), 128.72 (s, Car), 128.68 (s, Car), 126.86 (d, *J* = 3.4 Hz, Car), 84.97 (s, Cp), 62.13 (d, *J* = 5.6 Hz, OCH_2), 58.85 (s, C-C), 52.03 (s, C-C), 39.25 (s, NH-CH_2-ar), 33.65 (d, *J* = 138.2 Hz, CH_2-P), 16.35 (d, *J* = 5.9 Hz, CH_3) ppm. Anal. Calcd for $C_{23}H_{27}FeN_2O_7P$: C, 52.09; H, 5.13; N, 5.28. Found: C, 52.03; H, 5.31; N, 5.27. **IR** (ATR, cm^{-1}): 2046, 1994 (C≡O), 1639 (CO imide), 1241, 1219 (P=O), 1053, 1028 (P-O-CH_2CH_3). **ESI-MS** m/z calcd for $C_{23}H_{27}FeN_2O_7P$ $(M+H)^+$: 531.10; found: 531.08.

9c: 1 (0.10 g, 0.37 mmol), diethyl-4-(aminomethyl)benzylphosphonate **8c** (0.14 g, 0.54 mmol), K_2CO_3 (0.25 g, 1.81 mmol), 9 mL ethanol, 1 mL H_2O. **Yield:** 77.7 mg, 40%, orange-brown oil. **^{31}P{H}NMR** (162 MHz, $CDCl_3$): δ 26.4 ppm. **^{1}H NMR** (400 MHz, $CDCl_3$): δ 7.25 (s, 4H, CHar), 5.03 (s, 5H, Cp), 4.04–3.96 (m, 4H, OCH_2), 3.77 (q, *J* = 13.2 Hz, 2H, C<u>H$_2$</u>N), 3.61 (dd, *J* = 8.0, 5.0 Hz, 1H, <u>CH</u>-CH_2), 3.12 (d, *J* = 21.6 Hz, 2H, <u>CH$_2$</u>P), 2.77 (dd, *J* = 17.3, 8.1 Hz, 1H, CH-<u>CH$_2$</u>), 2.45 (dd, *J* = 17.2, 5.0 Hz, 1H, CH-<u>CH$_2$</u>), 1.24 (t, *J* = 7.1 Hz, 6H, CH_3) ppm. **^{13}C NMR** (151 MHz, $CDCl_3$) δ 212.14 (d, *J* = 11.5 Hz, 2 C≡O), 190.97 (s, C=O), 188.07 (s, C=O), 137.71 (s, Car), 130.65 (s, Car), 129.93 (d, *J* = 6.5 Hz, Car), 128.51 (s, 2Car), 84.95 (s, Cp), 62.11 (d, *J* = 6.7 Hz, OCH_2), 58.79 (s, C-C), 51.78 (s, C-C), 39.24 (s, CH_2N), 33.44 (d, *J* = 138.5 Hz, CH_2P), 16.36 (d, *J* = 6.0 Hz, CH_3) ppm. Anal. Calcd for $C_{23}H_{27}FeN_2O_7P$: C, 52.09; H, 5.13; N, 5.28. Found: C, 52.07; H, 5.22; N, 5.29. **IR** (ATR, cm^{-1}): 2045, 1994 (C≡O), 1641 (CO imide), 1223 (P=O), 1055, 1025 (P-O-CH_2CH_3). **ESI-MS** m/z calcd for $C_{23}H_{27}FeN_2O_7P$ $(M+H)^+$: 531.10; found: 531.13.

10a: 2 (0.02 g, 0.06 mmol), diethyl-2-(aminomethyl)benzylphosphonate **8a** (0.02 g, 0.08 mmol), K_2CO_3 (0.04 g, 0.29 mmol), 4 mL ethanol, 0.4 mL H_2O. **Yield:** 29 mg, 77%, orange-brown oil. **^{31}P{H}NMR** (243 MHz, $CDCl_3$): δ 26.8 ppm. **^{1}H NMR** (600 MHz, $CDCl_3$) δ 7.32 (d, *J* = 5.6 Hz, 1H, CHar), 7.29–7.26 (m, 1H, CHar), 7.22–7.18 (m, 2H, CHar), 5.44 (s, 5H, Cp), 4.02–3.96 (m, 4H, OCH_2), 3.97 – 3.86 (m, 2H, N-C<u>H$_2$</u>-Ar), 3.77 (dd, *J* = 8.2, 5.1 Hz, 1H, <u>CH</u>-CH_2), 3.34 (ddd, *J* = 43.6, 21.8, 14.9 Hz, 2H, <u>CH$_2$</u>P), 2.86 (dd, *J* = 17.3, 8.2 Hz, 1H, CH-<u>CH$_2$</u>),), 2.51 (dd, *J* = 17.3, 5.0 Hz, 1H, CH-<u>CH$_2$</u>), 1.22 (dt, *J* = 0.4, 7.0 Hz, 6H, CH_3) ppm. **^{13}C NMR** (151 MHz, $CDCl_3$) δ 196.67 (s, 2 C≡O), 189.72 (s, C=O), 186.78 (s, C=O), 138.00 (s, Car), 131.28 (d, *J* = 5.5 Hz, Car), 130.63 (d, *J* = 9.3 Hz, Car), 130.18 (s, Car), 127.55 (s, Car), 127.24 (d, *J* = 3.8 Hz, Car), 86.85 (s, Cp), 62.13 (t, *J* = 6.4 Hz, OCH_2), 59.17 (s, C-C), 50.00 (s, C-C), 38.80 (s, CH_2N), 30.40 (d, *J* = 137.6 Hz, CH_2-P), 16.36 (d, *J* = 5.9 Hz, CH_3) ppm. Anal. Calcd for $C_{23}H_{27}RuN_2O_7P$: C, 48.00; H, 4.73; N, 4.87. Found: C, 48.13; H, 4.74; N, 4.74. **IR** (ATR, cm^{-1}): 2045, 1990 (C≡O), 1643 (CO imide), 1228 (P=O), 1053, 1025 (P-O-CH_2CH_3). **ESI-MS** m/z calcd for $C_{23}H_{27}RuN_2O_7P$ $(M+H)^+$: 577.06; found: 577.05.

10b: 2 (0.04 g, 0.12 mmol), diethyl-3-(aminomethyl)benzylphosphonate **8b** (0.04 g, 0.16 mmol), K_2CO_3 (0.08 g, 0.58 mmol), 9 mL ethanol, 1 mL H_2O. **Yield:** 40 mg, 60%, orange-brown oil. **^{31}P{H}NMR** (243 MHz, $CDCl_3$): δ 26.3 ppm. **^{1}H NMR** (600 MHz, $CDCl_3$) δ 7.27 (d, *J* = 7.5 Hz, 1H, CHar), 7.26–7.18 (m, 3H, CHar), 5.44 (s, 5H, Cp), 4.07–3.97 (m, 4H, OCH_2), 3.83 (q, *J* = 13.2 Hz, 2H, C<u>H$_2$</u>N), 3.72 (dd, *J* = 8.1, 5.1 Hz, 1H, <u>CH</u>-CH_2), 3.13 (d, *J* = 21.6 Hz, 2H, <u>CH$_2$</u>P), 2.85 (dd, *J* = 17.3, 8.1 Hz, 1H, CH-<u>CH$_2$</u>), 2.54 (dd, *J* = 17.3, 5.1 Hz, 1H, CH-<u>CH$_2$</u>), 1.24 (t, *J* = 7.1 Hz, 6H, CH_3) ppm. **^{13}C NMR** (151 MHz, $CDCl_3$) δ 196.61 (d, *J* = 4.3 Hz, 2 C≡O), 189.26 (s, C=O), 186.59 (s, C=O), 139.05 (s, Car), 131.96 (d, *J* = 9.2 Hz, Car), 129.92 (d, *J* = 6.7 Hz, Car), 128.84 (d, *J* = 6.5 Hz, Car), 128.75 (d, *J* = 3.0 Hz, Car), 127.00 (d, *J* = 3.6 Hz, Car), 86.88 (s, Cp), 62.14 (dd, *J* = 6.8, 2.8 Hz, OCH_2), 58.34 (s, C-C), 51.88 (s, C-C), 38.61 (s, CH_2N), 33.67 (d, *J* = 138.0 Hz, CH_2P), 16.37 (d, *J* = 5.9 Hz, CH_3) ppm. Anal. Calcd for $C_{23}H_{27}RuN_2O_7P$: C, 48.00; H, 4.73; N, 4.87. Found: C, 47.99; H, 4.71; N, 4.67. **IR** (ATR, cm^{-1}): 2046, 1988 (C≡O), 1643 (CO imide), 1224 (P=O), 1055, 1028, (P-O-CH_2CH_3). **ESI-MS** m/z calcd for $C_{23}H_{27}RuN_2O_7P$ $(M+H)^+$: 577.06; found: 577.10.

10c: 2 (0.02 g, 0.06 mmol), diethyl-4-(aminomethyl)benzylphosphonate **8c** (0.02 g, 0.08 mmol), K_2CO_3 (0.04 g, 0.29 mmol), 4 mL ethanol, 0.4 mL H_2O. **Yield:** 19 mg, 54%, orange-brown oil. **^{31}P{H}NMR** ($CDCl_3$, 243MHz): δ 26.3 ppm. **^{1}H NMR** (600 MHz, $CDCl_3$) δ 7.38–7.20 (m, 4H, CHar), 5.43 (s, 5H, Cp), 4.04–3.96 (m, 4H, OCH_2), 3.81 (q, *J* = 13.1 Hz, 2H, C<u>H$_2$</u>N), 3.71 (dd, *J* = 8.1, 5.1 Hz, 1H, <u>CH</u>-CH_2), 3.12 (d, *J* = 21.6 Hz, 2H, <u>CH$_2$</u>P), 2.84 (dd, *J* = 17.2, 8.1 Hz, 1H, CH-<u>CH$_2$</u>), 2.53 (dd, *J* = 17.3, 5.0 Hz, 1H, CH-<u>CH$_2$</u>),

1.24 (t, *J* = 7.1 Hz, 6H, CH_3) ppm. **^{13}C NMR** (151 MHz, $CDCl_3$) δ 196.59 (s, 2 C≡O), 186.68 (s, 2C=O), 130.73 (d, *J* = 9.3 Hz, 2Car), 129.95 (d, *J* = 6.6 Hz, 2Car), 128.55 (d, *J* = 2.9 Hz, 2Car), 86.84 (s, Cp), 62.12 (d, *J* = 6.8 Hz, OCH_2), 58.43 (s, C-C), 51.74 (s, C-C), 38.74 (s, CH_2N), 33.45 (d, *J* = 138.4 Hz, CH_2P), 16.37 (d, *J* = 6.0 Hz, CH_3) ppm. Anal. Calcd for $C_{23}H_{27}RuN_2O_7P$: C, 48.00; H, 4.73; N, 4.87. Found: C, 48.18; H, 4.84; N, 4.75. **IR** (ATR, cm^{-1}): 2045, 1990 (C≡O), 1641 (CO imide), 1223 (P=O), 1051, 1023 (P-O-CH_2CH_3). **ESI-MS** m/z calcd for $C_{23}H_{27}RuN_2O_7P$ $(M+H)^+$: 577.06; found: 576.94.

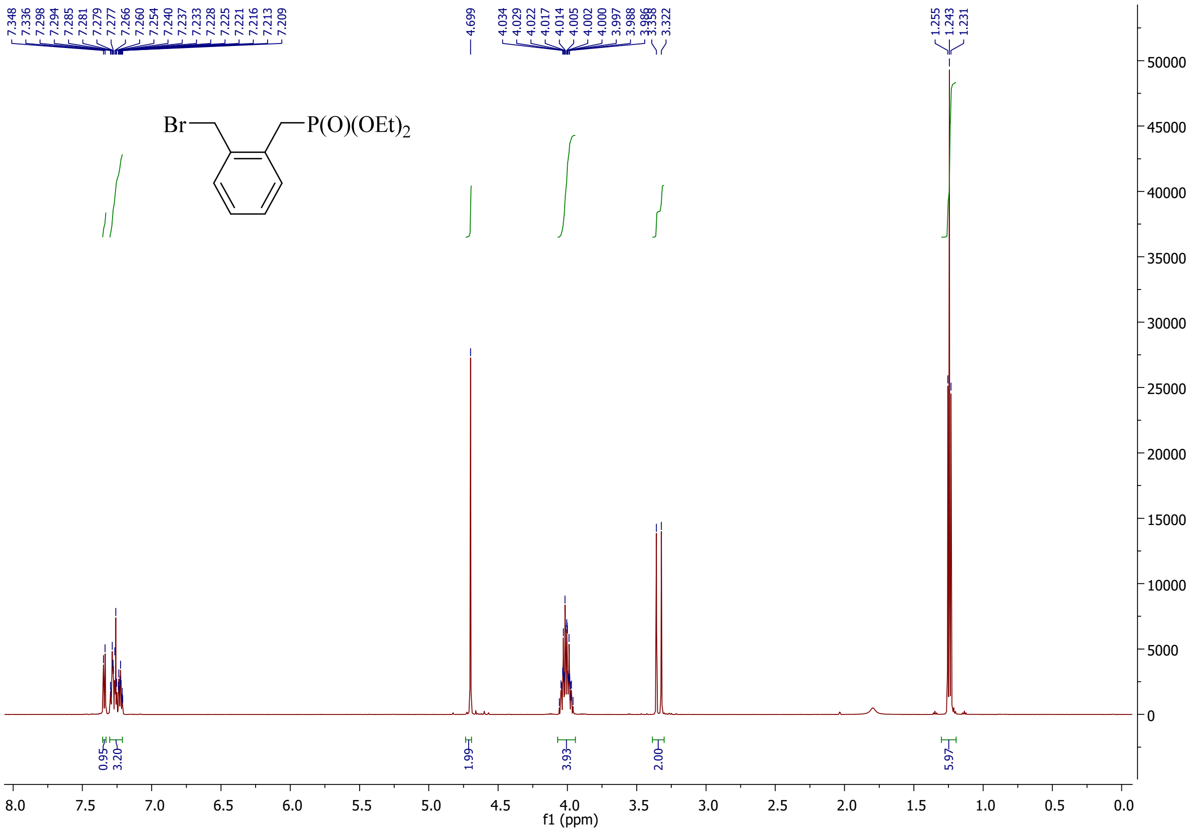

Supplementary Figure S1. 600 MHz ¹H NMR spectrum of **6a** in $CDCl_3$.

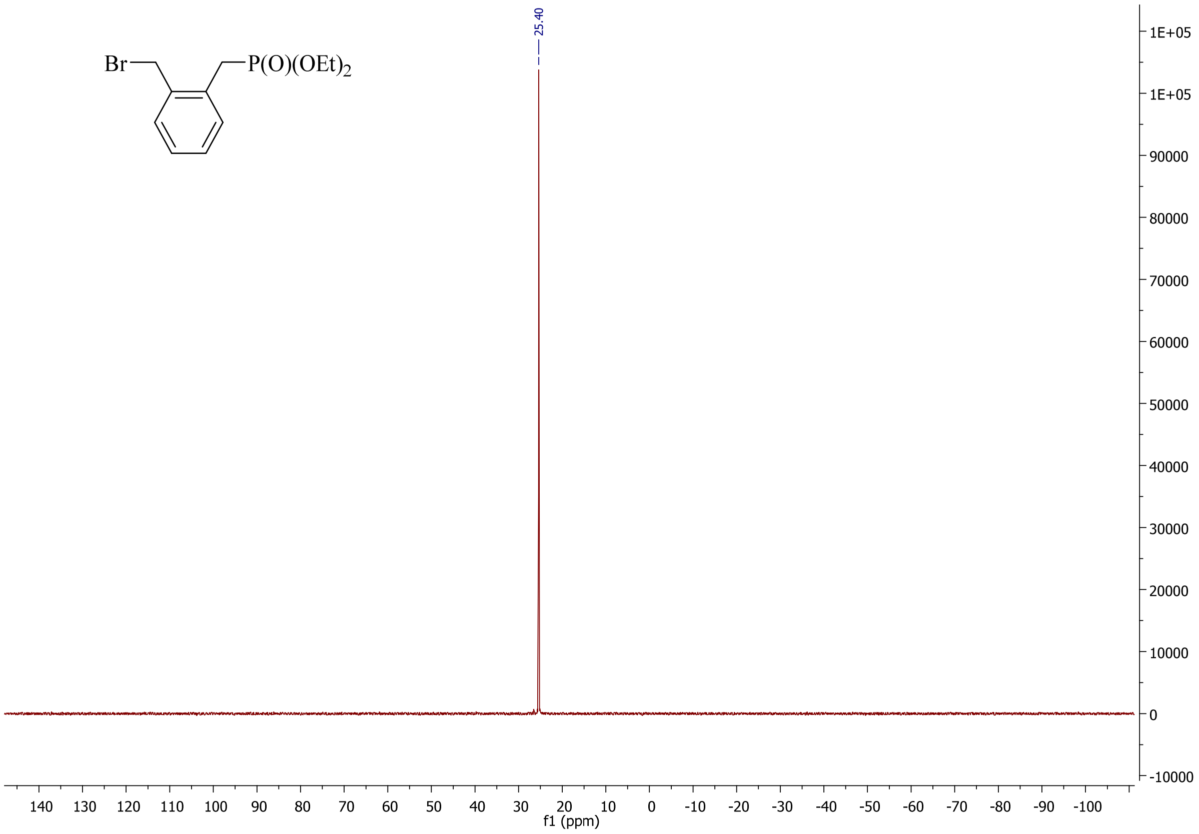

Supplementary Figure S2. 243 MHz $^{31}P\{H\}$NMR spectrum of **6a** in $CDCl_3$.

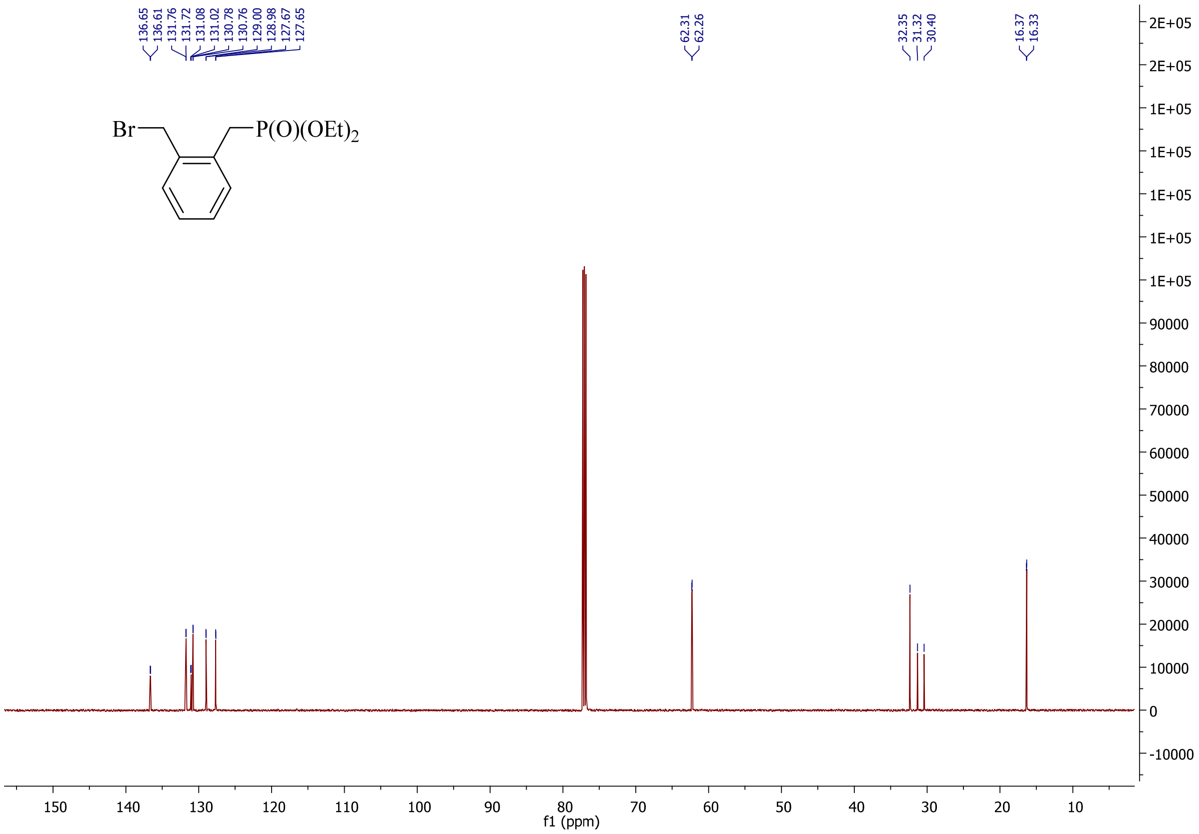

Supplementary Figure S3. 151 MHz ^{13}C NMR spectrum of **6a** in $CDCl_3$.

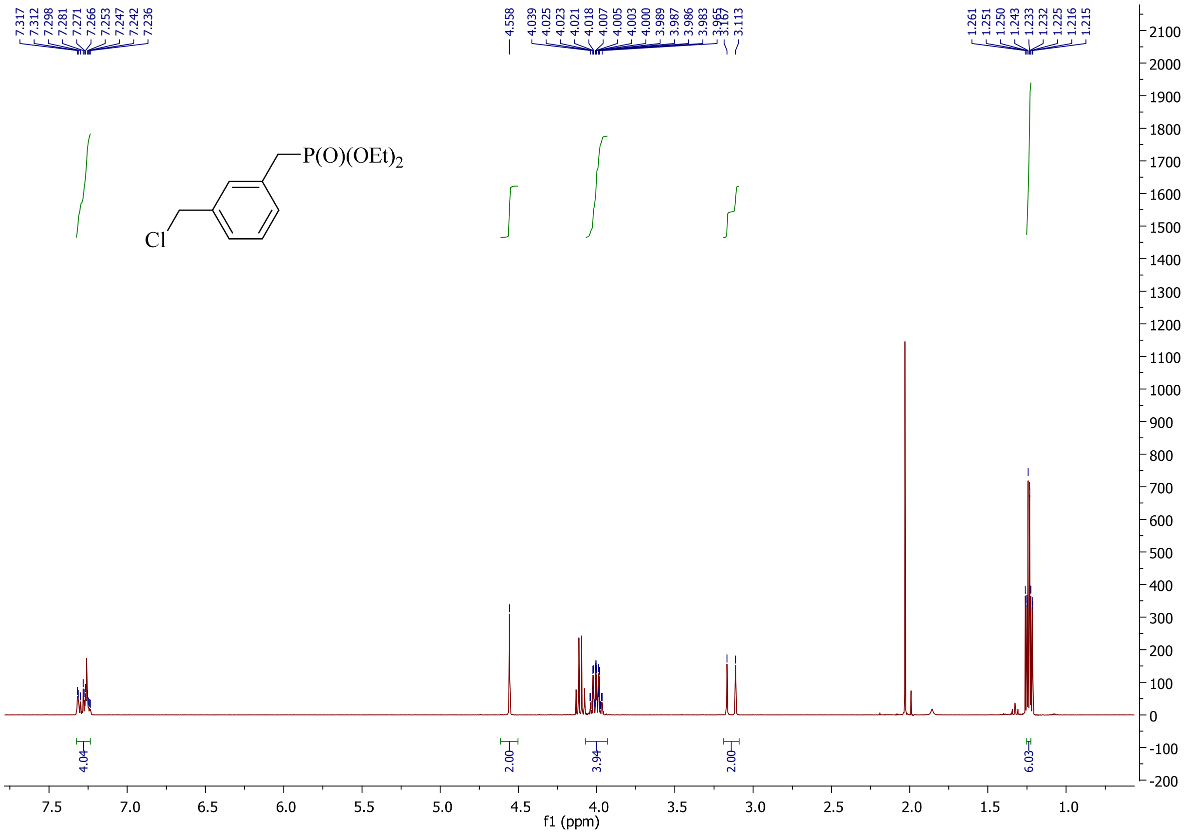

Supplementary Figure S4. 400 MHz ^{1}H NMR spectrum of **6b** in $CDCl_3$.

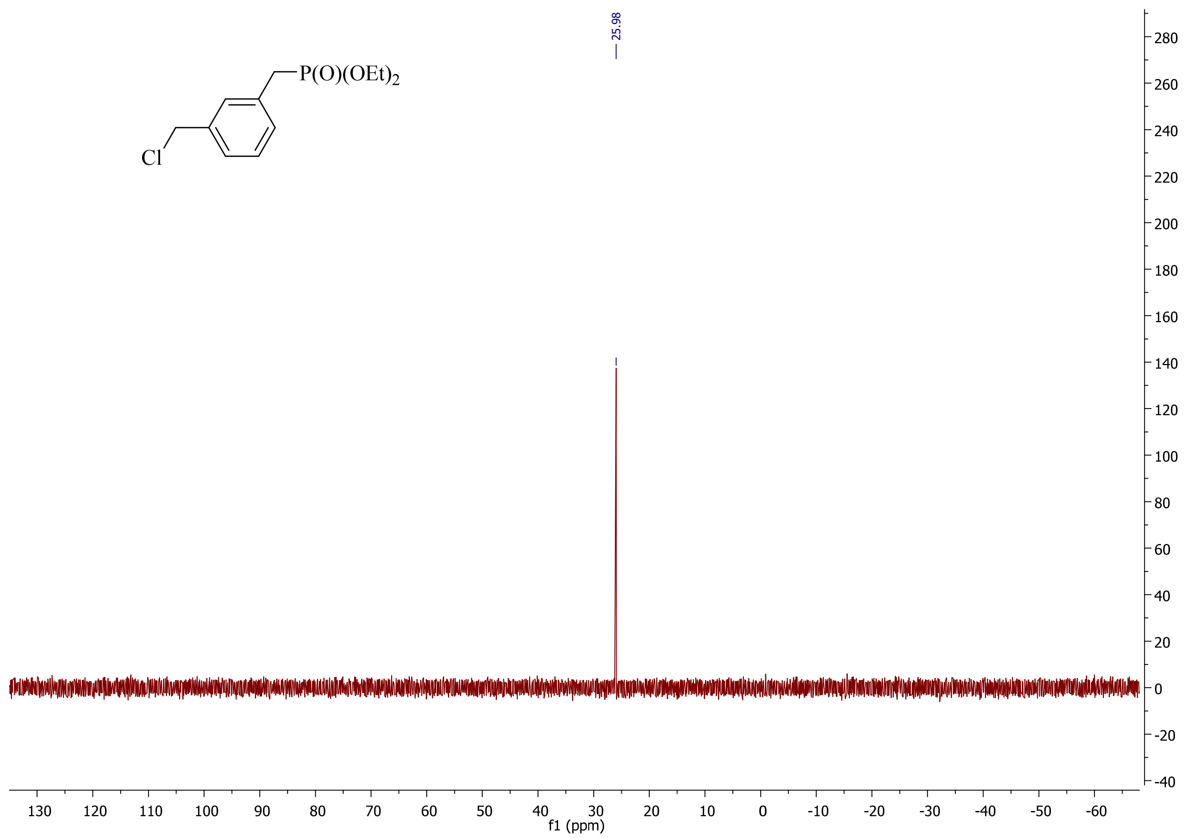

Supplementary Figure S5. 162 MHz $^{31}P\{H\}$NMR spectrum of **6b** in $CDCl_3$.

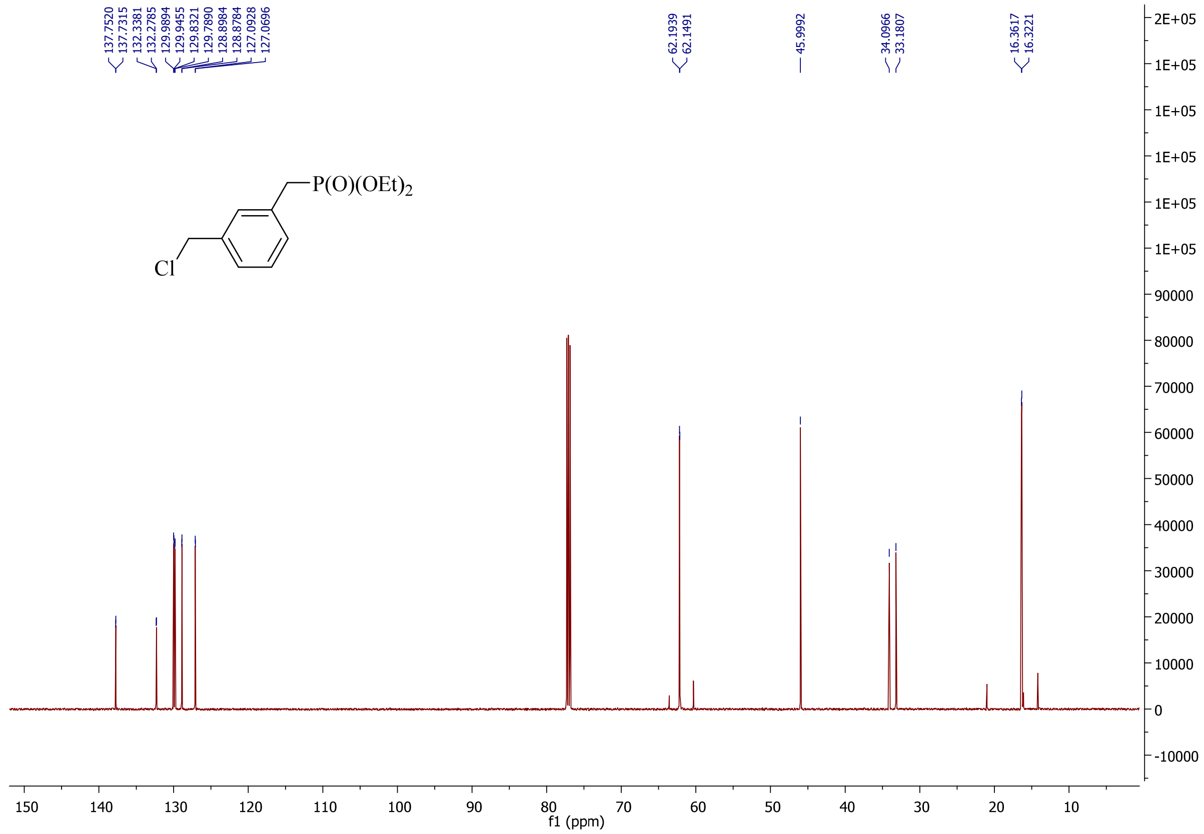

Supplementary Figure S6. 151 MHz ^{13}C NMR spectrum **6b** in $CDCl_3$.

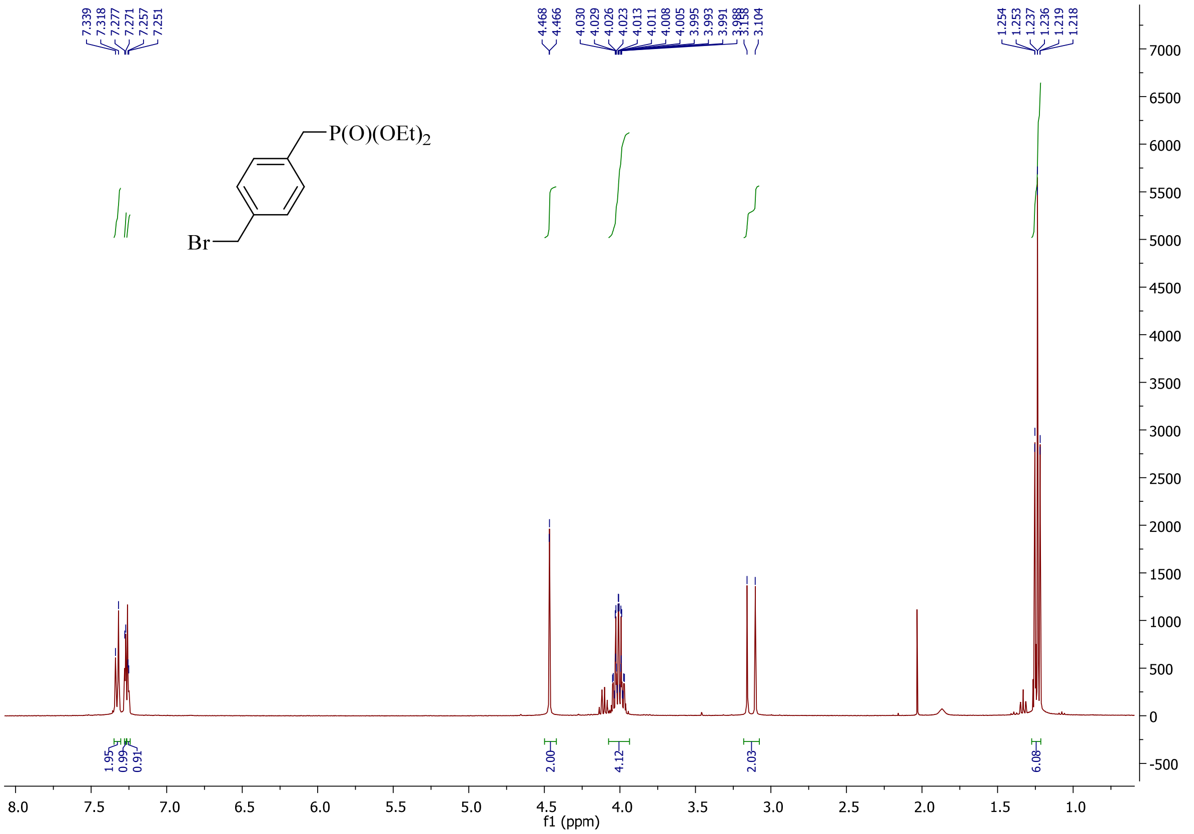

Supplementary Figure S7. 400 MHz ^{1}H NMR spectrum of **6c** in $CDCl_3$.

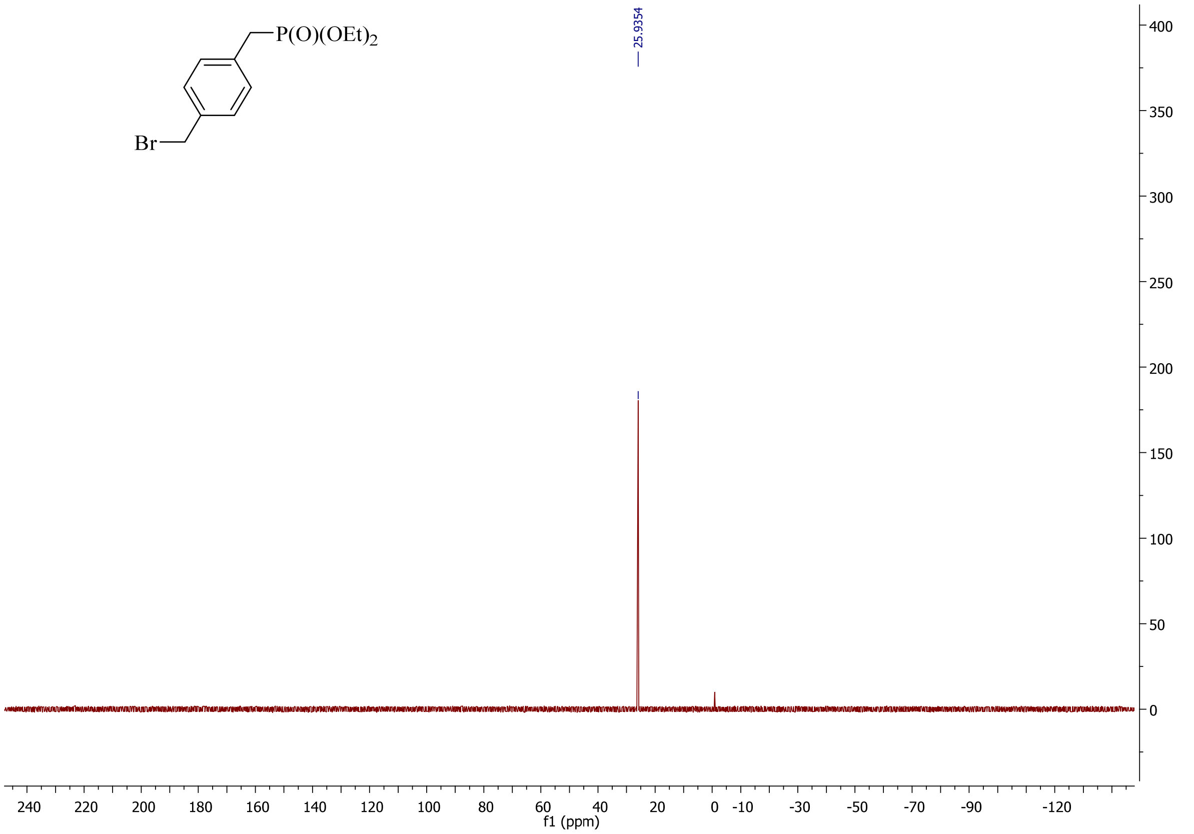

Supplementary Figure S8. 162 MHz $^{31}P\{H\}$NMR spectrum of **6c** in $CDCl_3$.

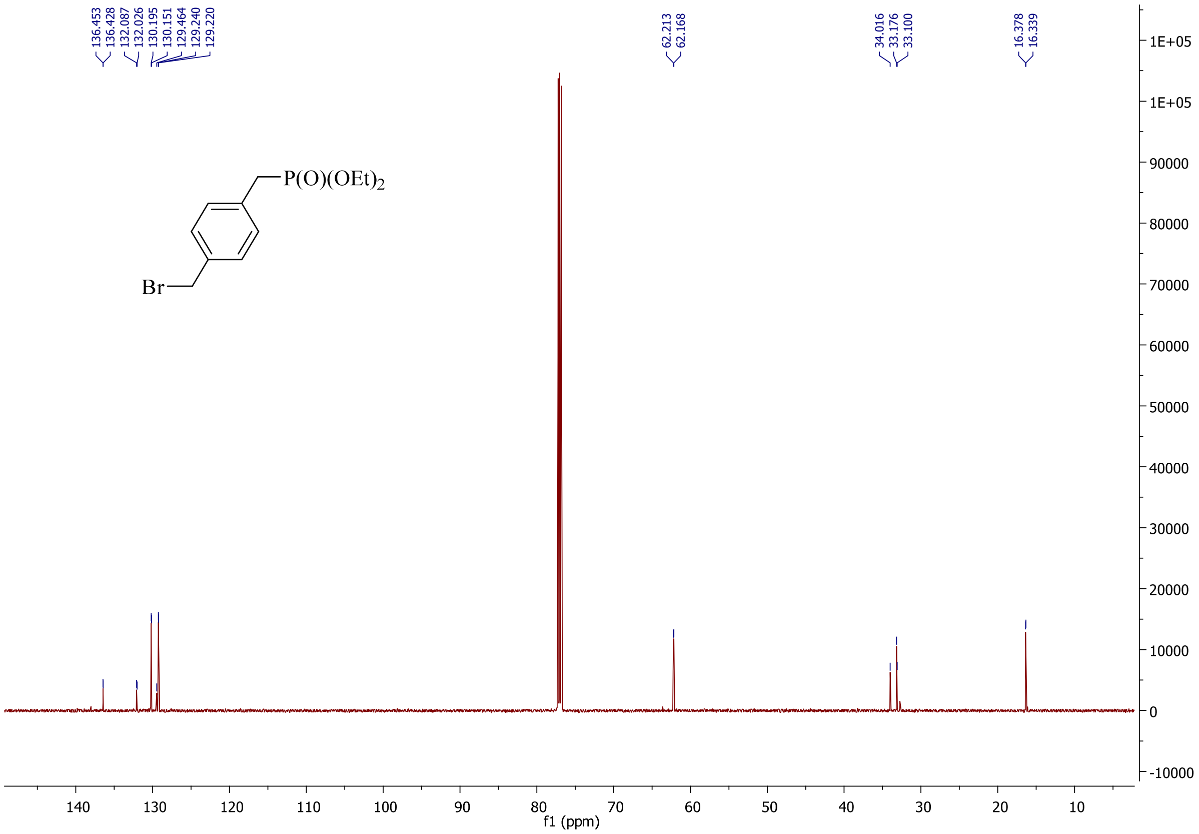

Supplementary Figure S9. 151 MHz ^{13}C NMR spectrum of **6c** in $CDCl_3$.

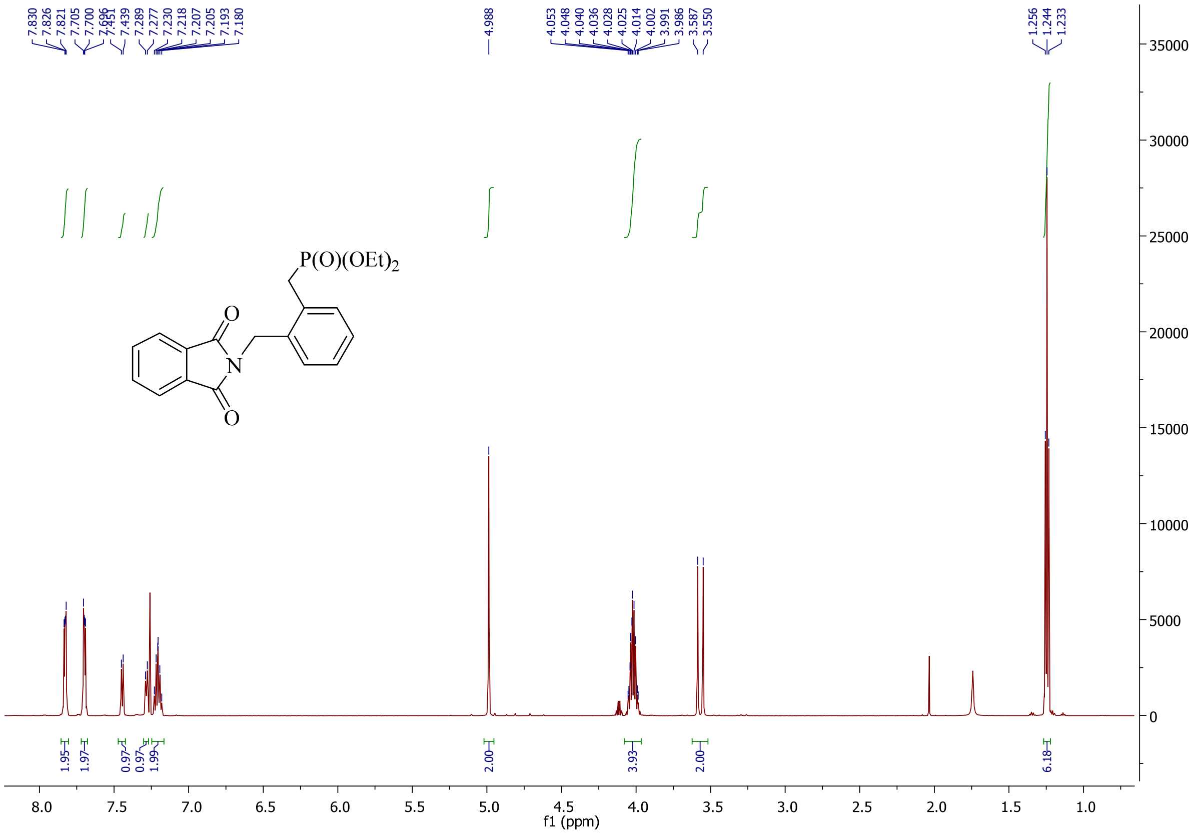

Supplementary Figure S10. 600 MHz ^{1}H NMR spectrum of **7a** in $CDCl_3$.

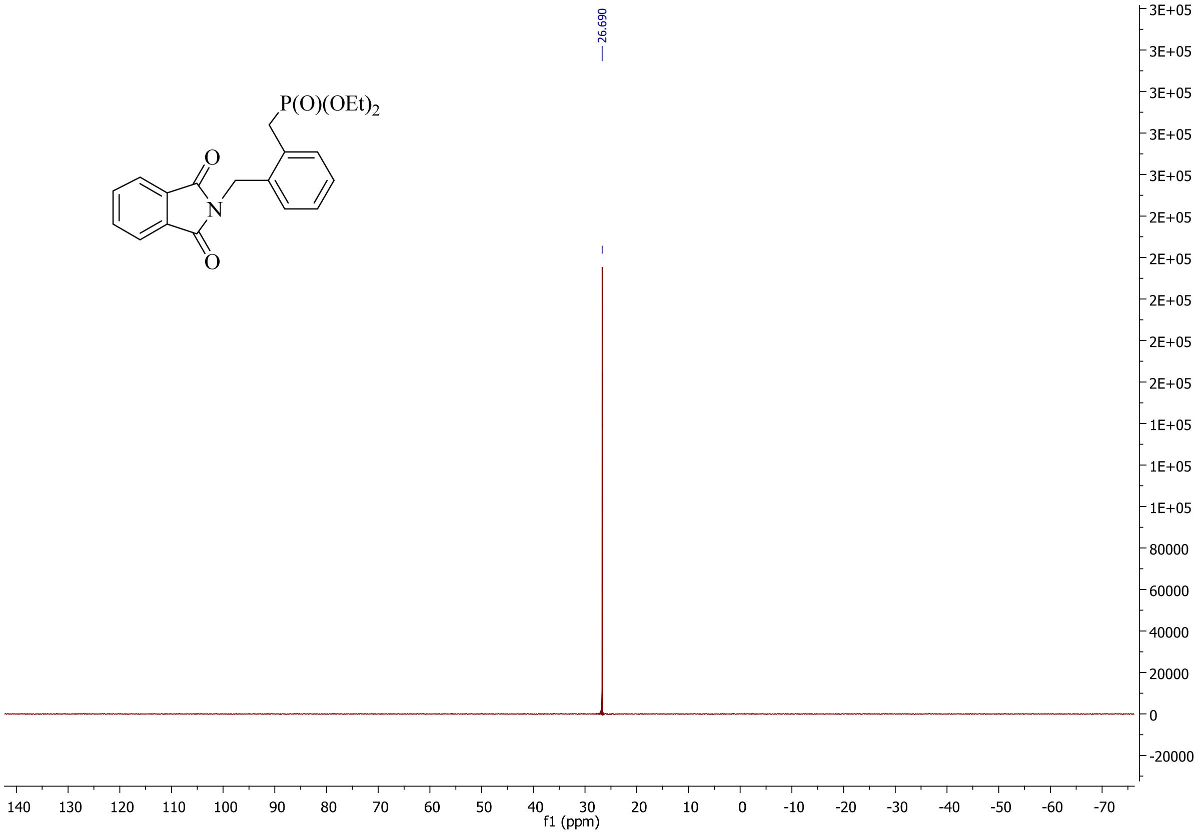

Supplementary Figure S11. 162 MHz $^{31}P\{H\}$NMR spectrum of **7a** in $CDCl_3$.

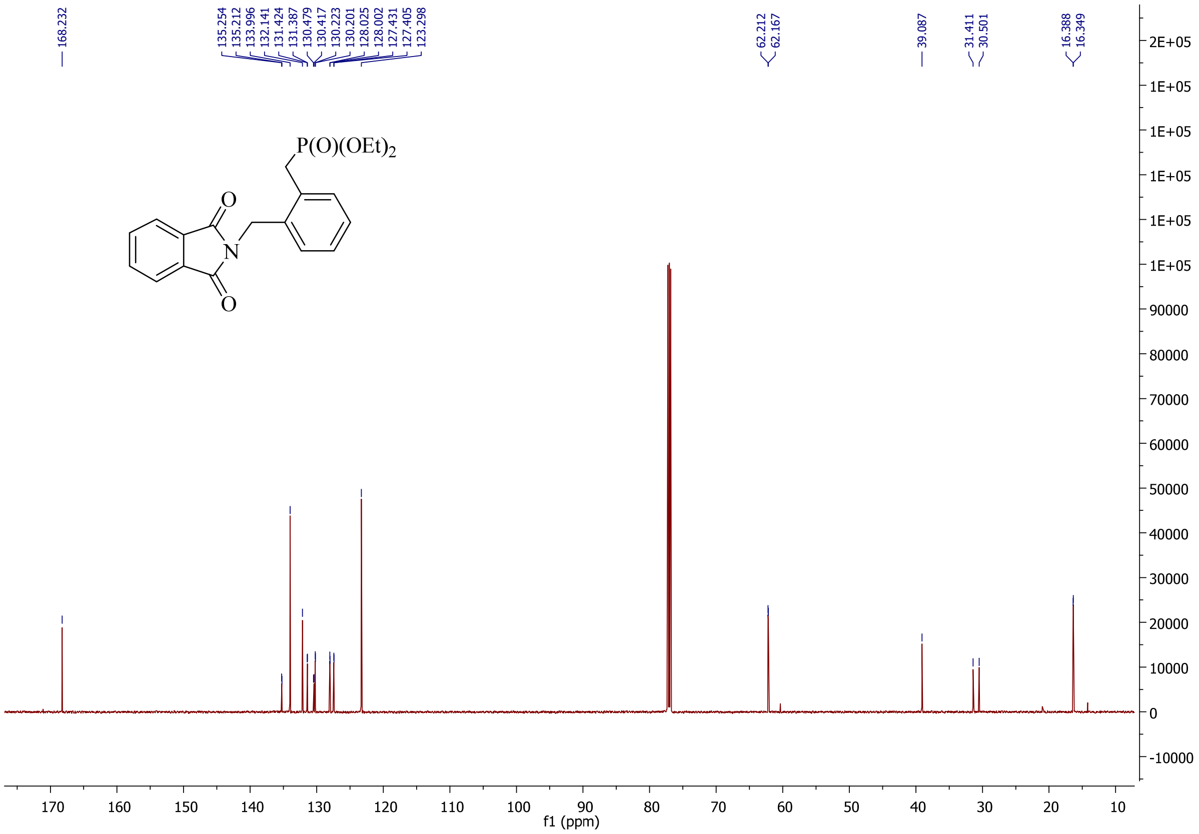

Supplementary Figure S12. 151 MHz ^{13}C NMR spectrum of **7a** in $CDCl_3$.

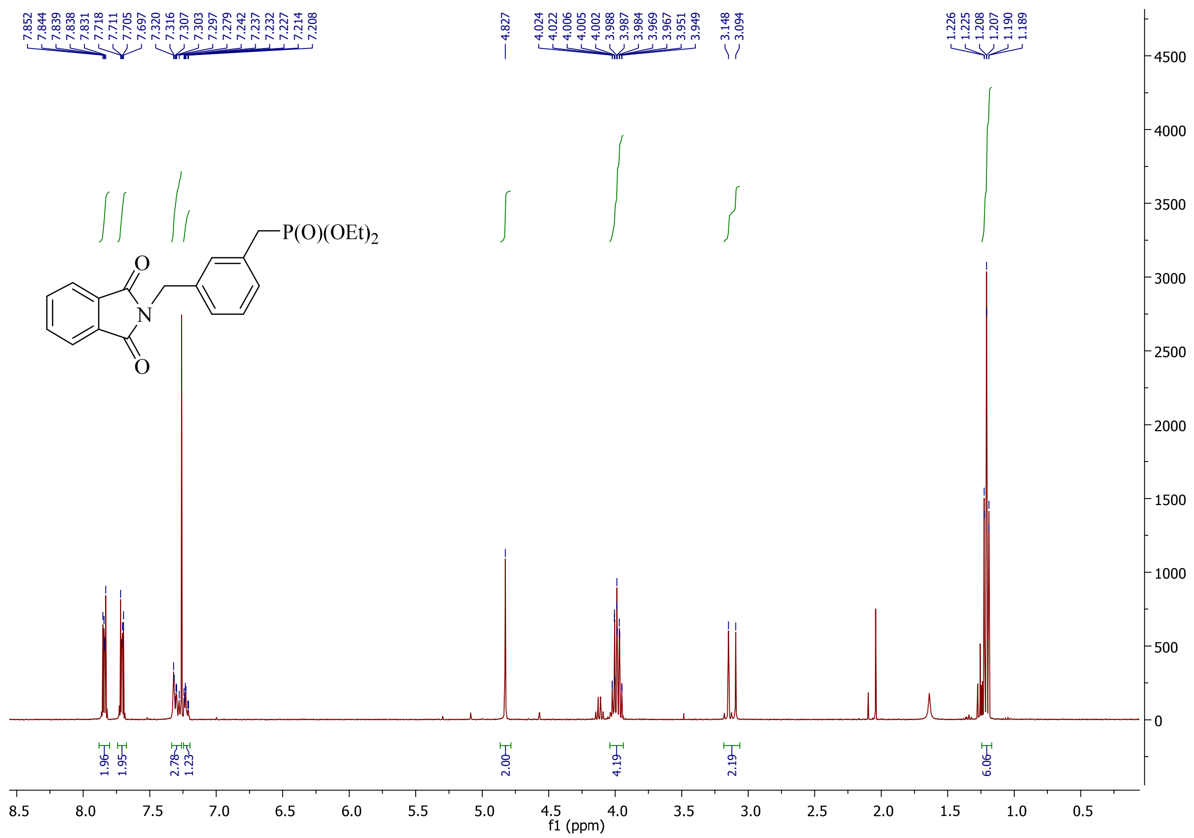

Supplementary Figure S13. 400 MHz ^{1}H NMR spectrum of **7b** in $CDCl_3$.

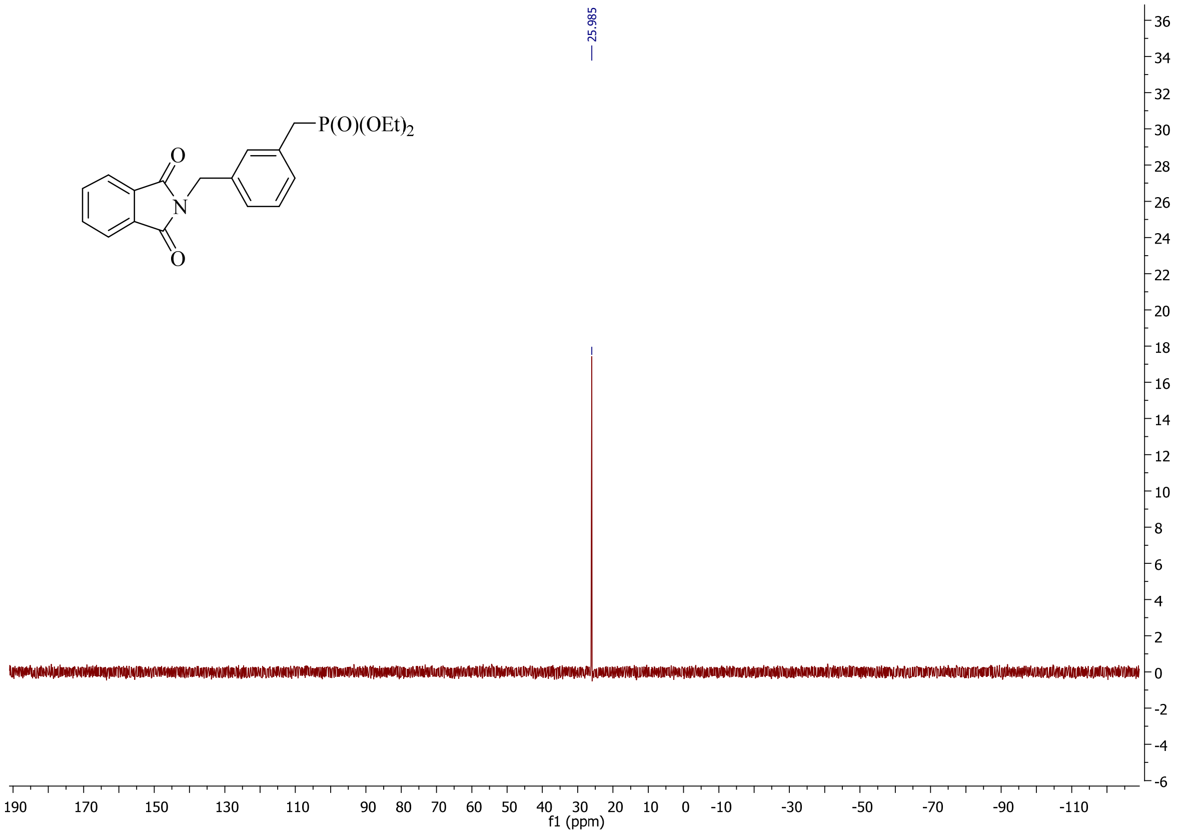

Supplementary Figure S14. 162 MHz ^{31}P{H}NMR spectrum of **7b** in $CDCl_3$.

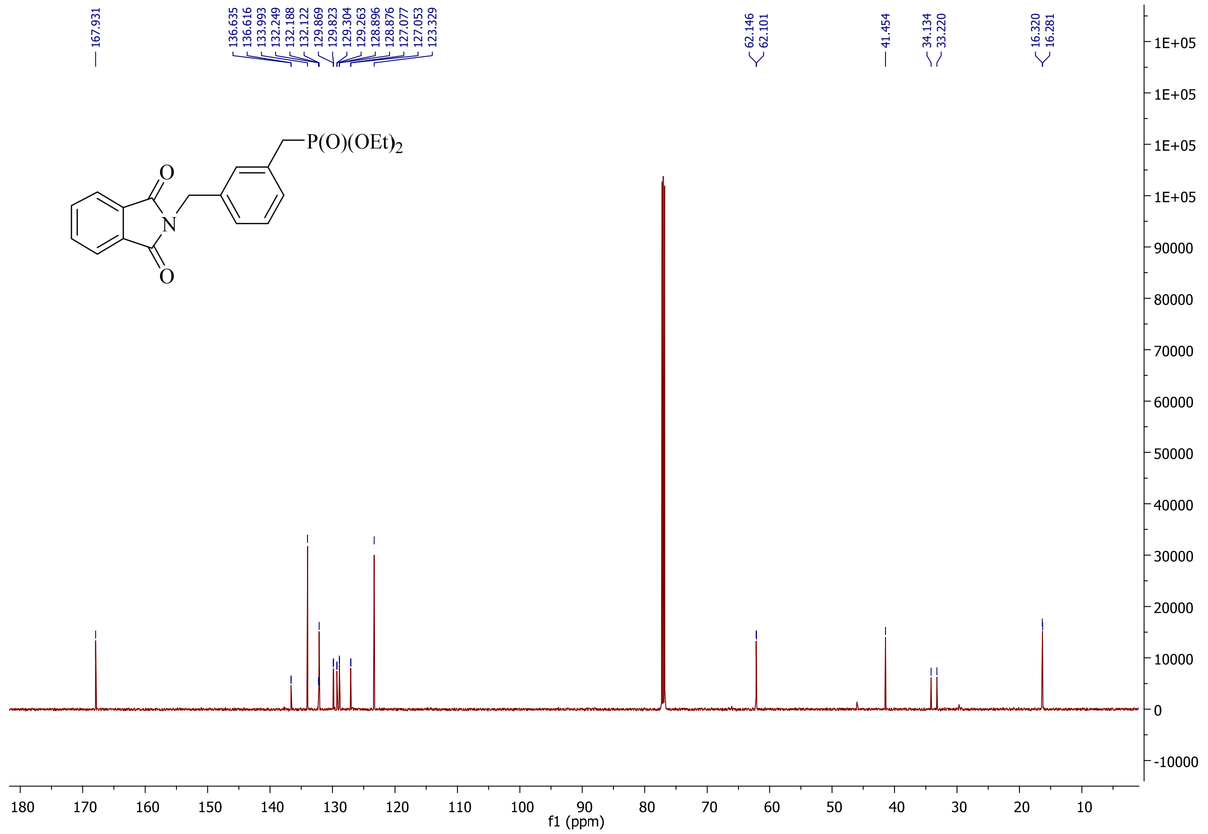

Supplementary Figure S15. 151 MHz ^{13}C NMR spectrum of **7b** in $CDCl_3$.

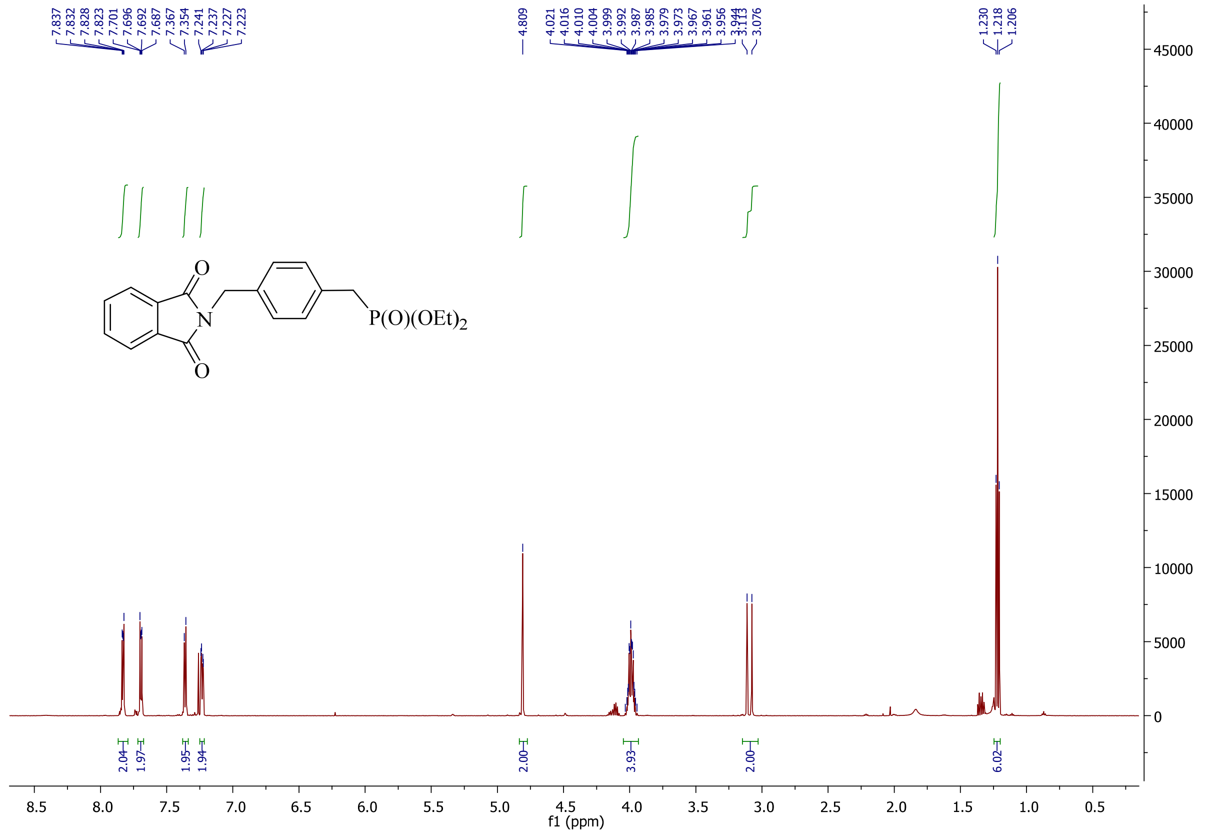

Supplementary Figure S16. 400 MHz ^{1}H NMR spectrum of **7c** in $CDCl_3$.

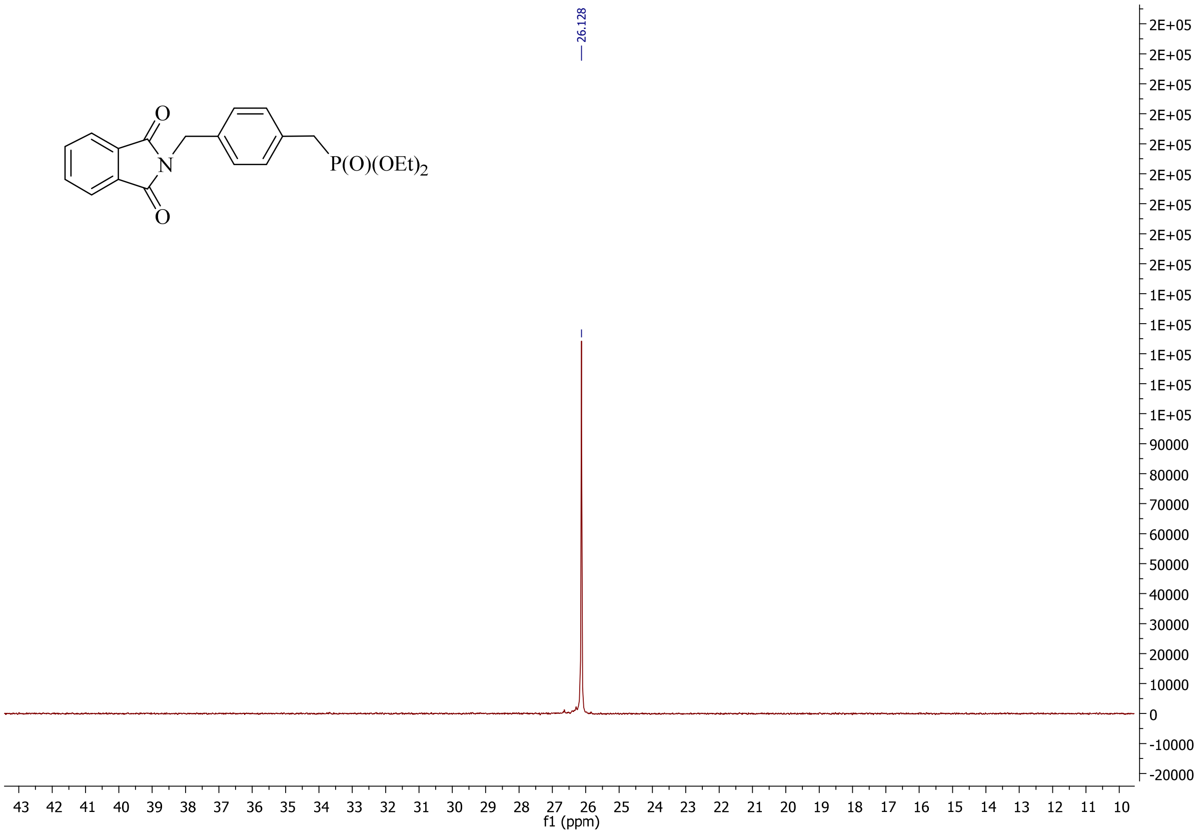

Supplementary Figure S17. 162 MHz $^{31}P\{H\}$NMR spectrum of **7c** in $CDCl_3$.

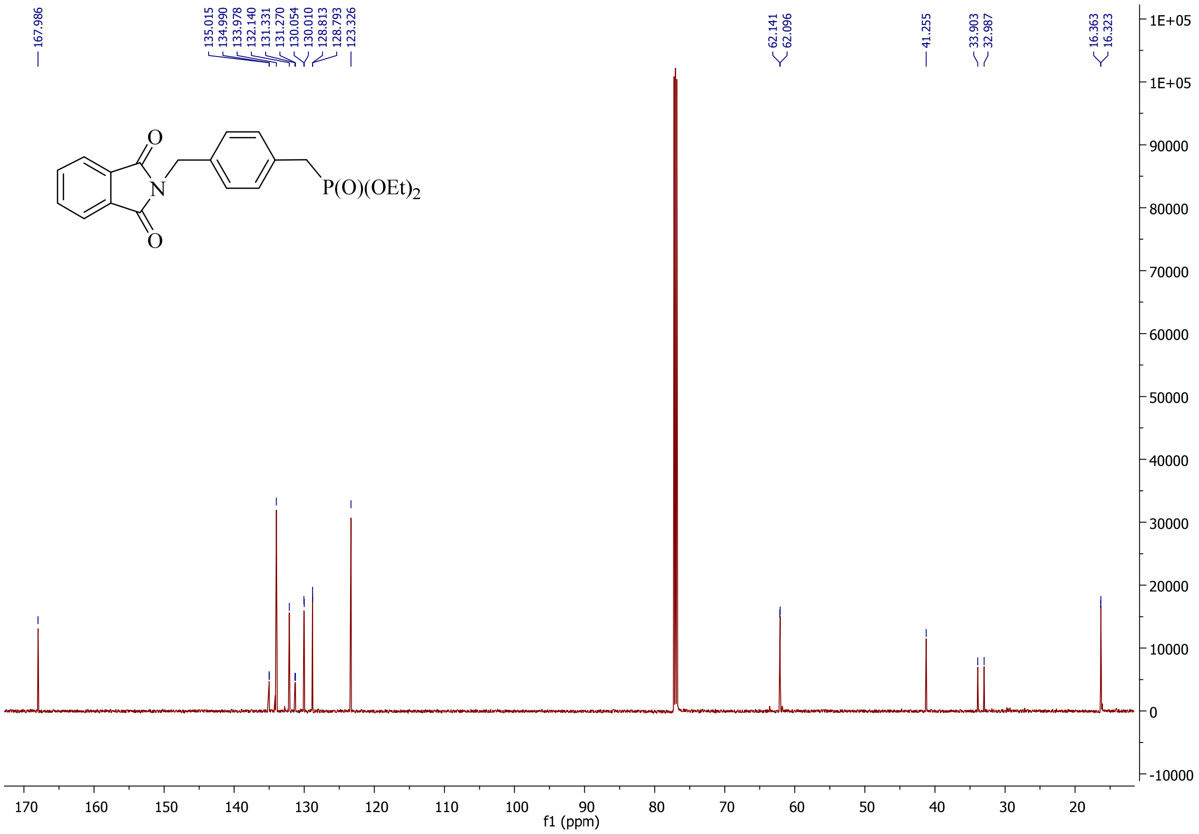

Supplementary Figure S18. 151 MHz ^{13}C NMR spectrum of **7c** in $CDCl_3$.

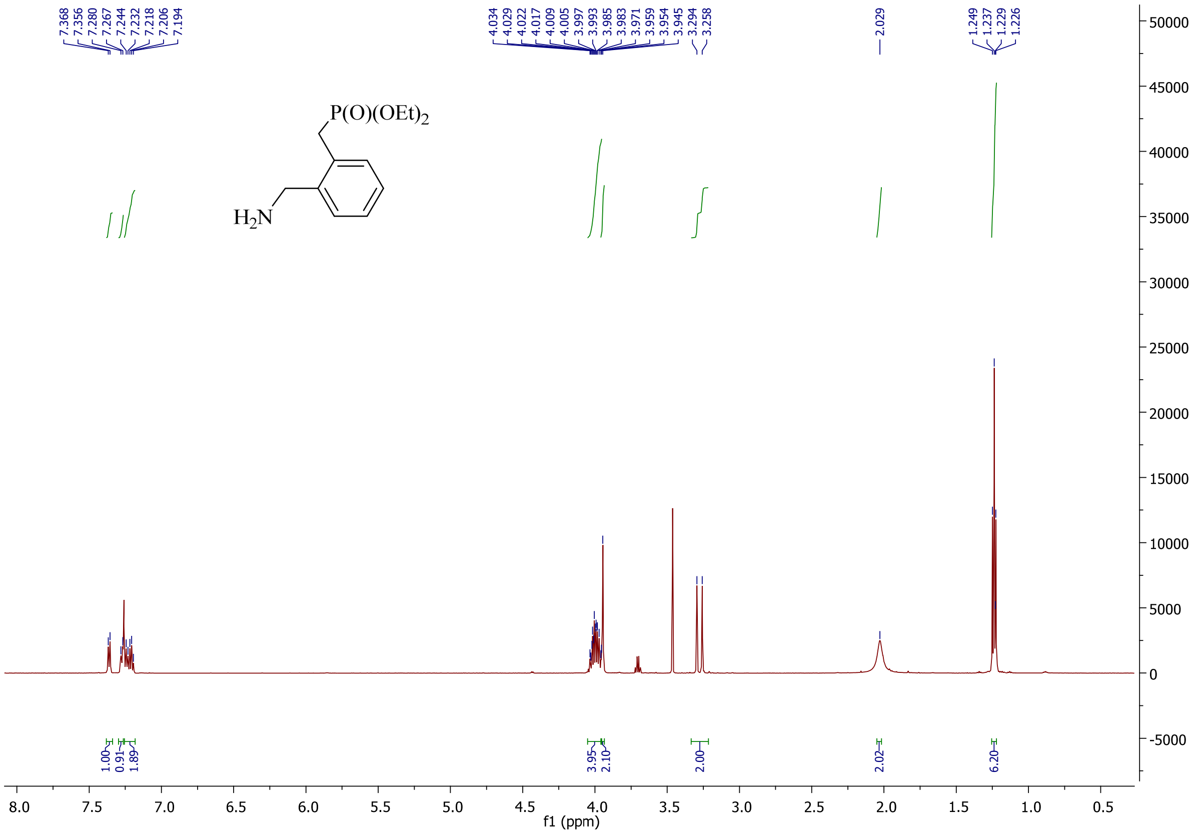

Supplementary Figure S19. 600 MHz ¹H NMR spectrum of **8a** in CDCl₃.

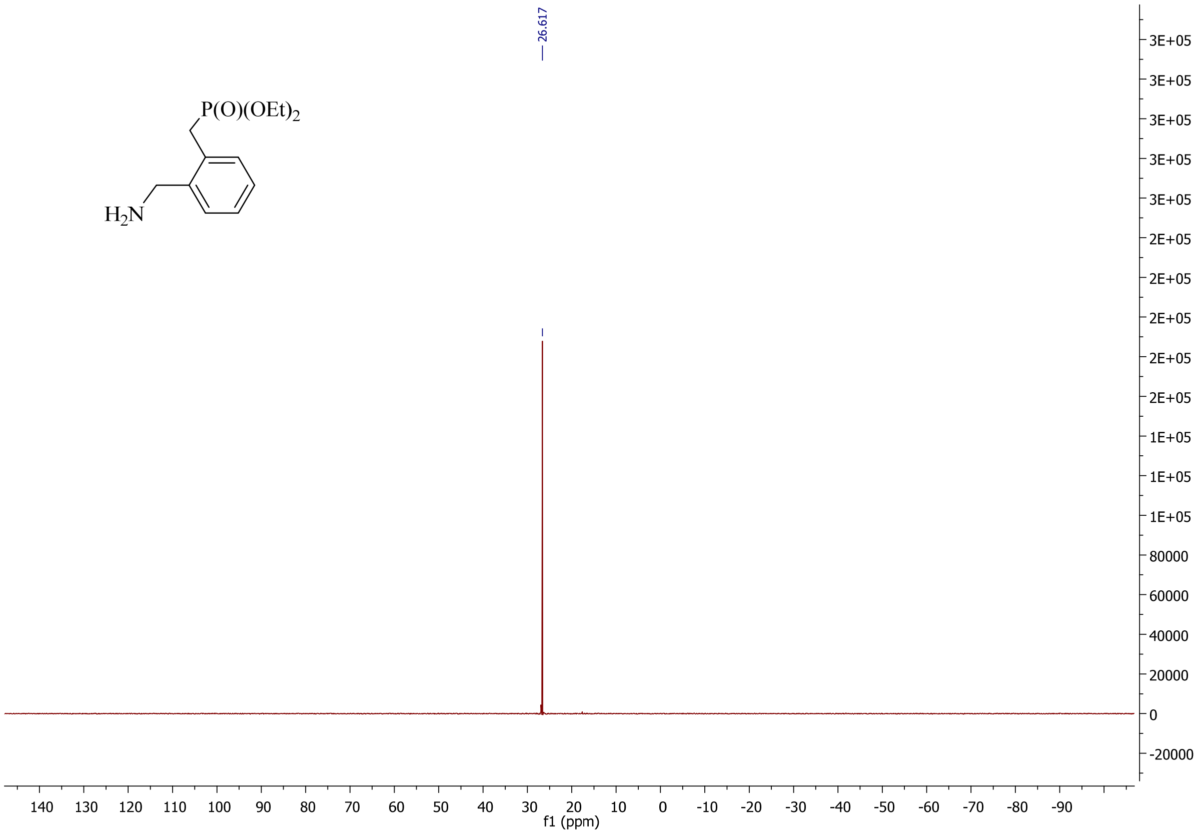

Supplementary Figure S20. 243 MHz ³¹P{H}NMR spectrum of **8a** in CDCl₃.

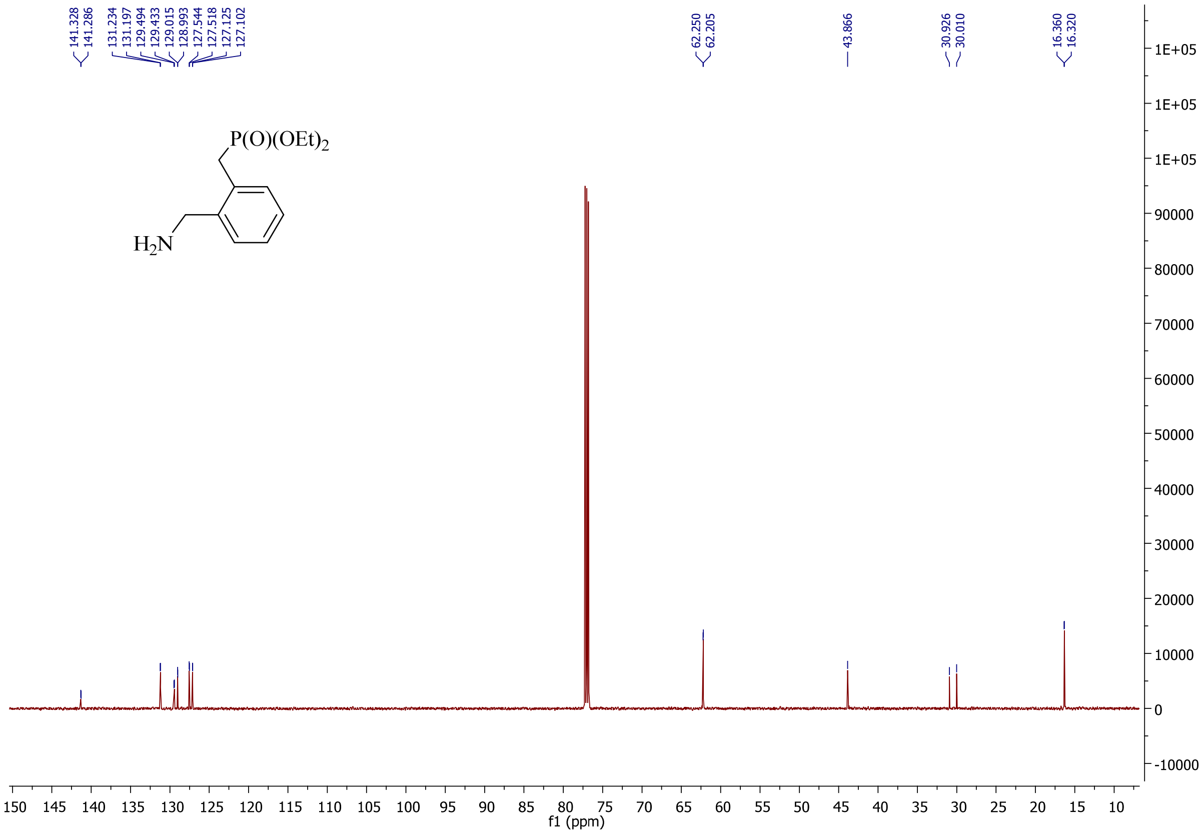

Supplementary Figure S21. 151 MHz ^{13}C NMR spectrum of **8a** in $CDCl_3$.

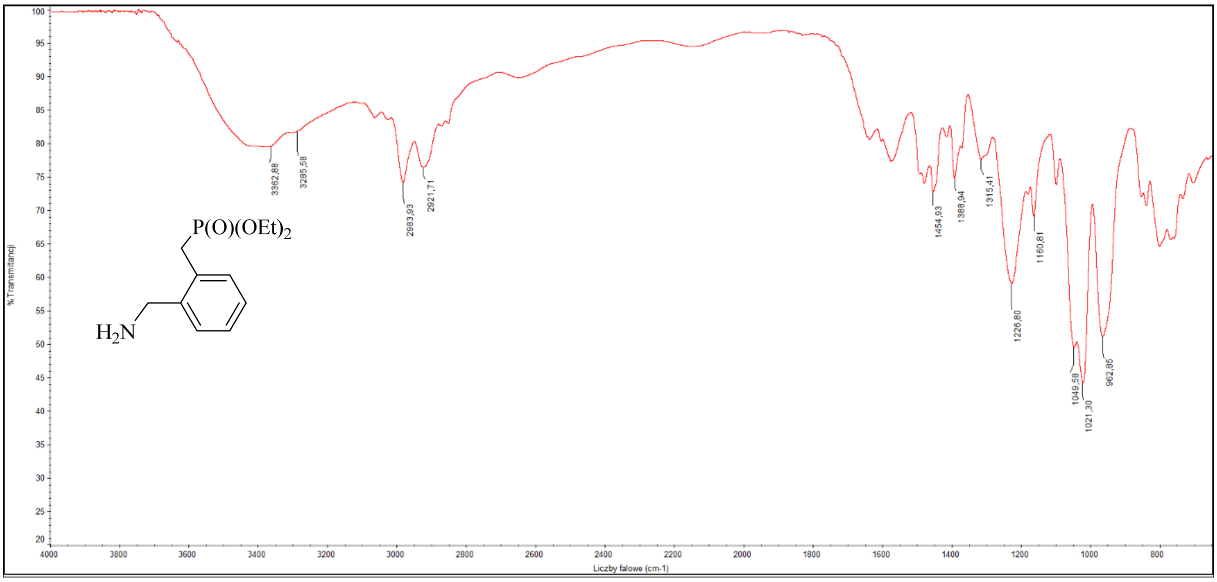

Supplementary Figure S22. ATR, IR spectrum of **8a**.

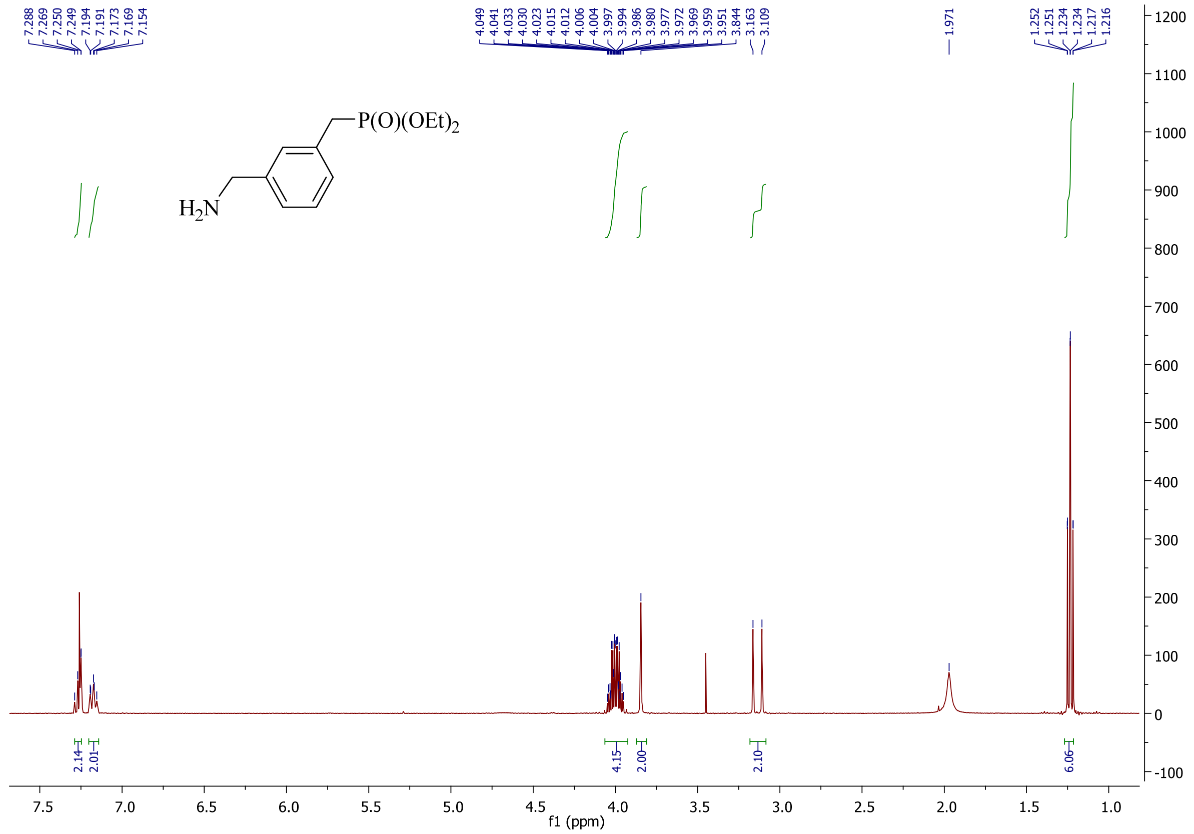

Supplementary Figure S23. 400 MHz ^{1}H NMR spectrum of **8b** in CDCl3.

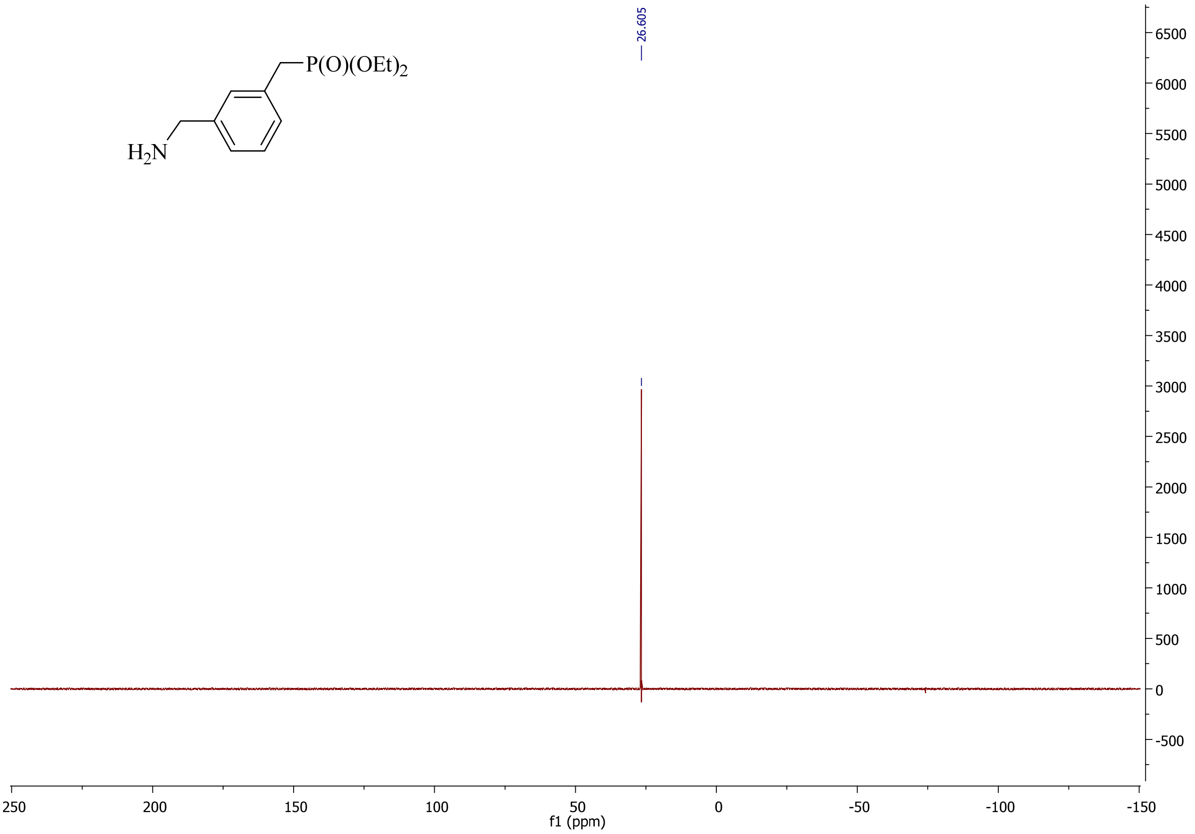

Supplementary Figure S24. 162 MHz ^{31}P{H}NMR spectrum of **8b** in CDCl3.

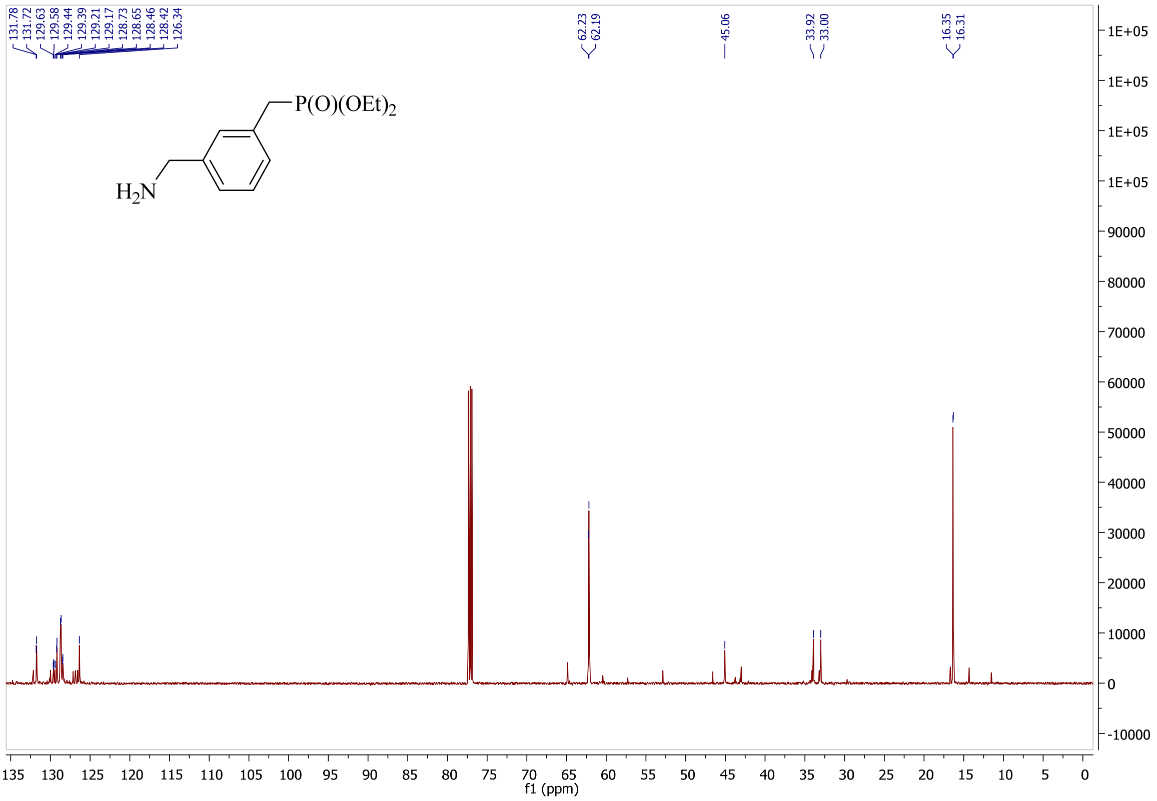

Supplementary Figure S25. 151 MHz ^{13}C NMR spectrum of **8b** in $CDCl_3$.

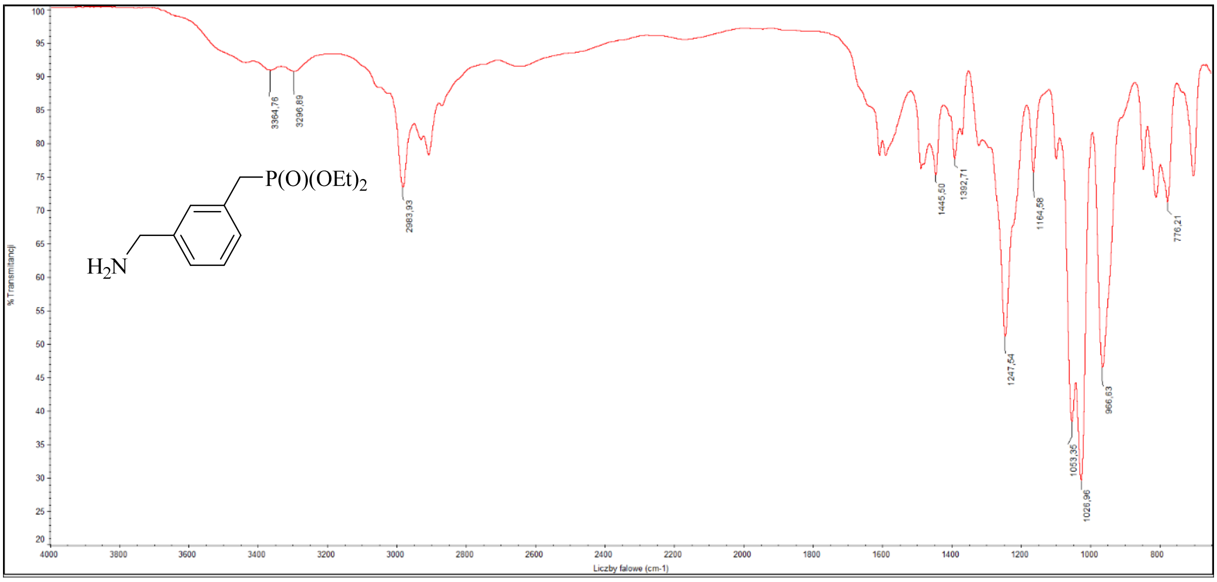

Supplementary Figure S26. ATR, IR spectra of **8b**.

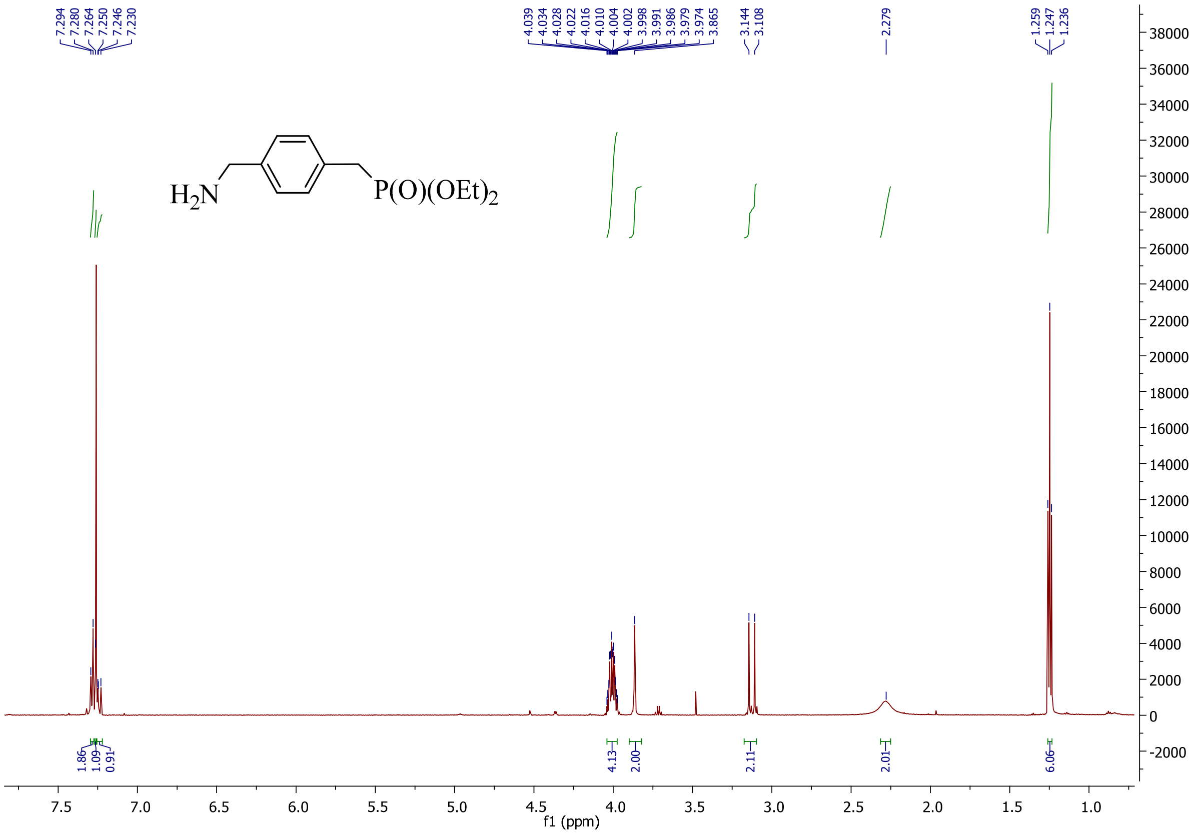

Supplementary Figure S27. 400 MHz ^{1}H NMR spectrum of **8c** in $CDCl_3$.

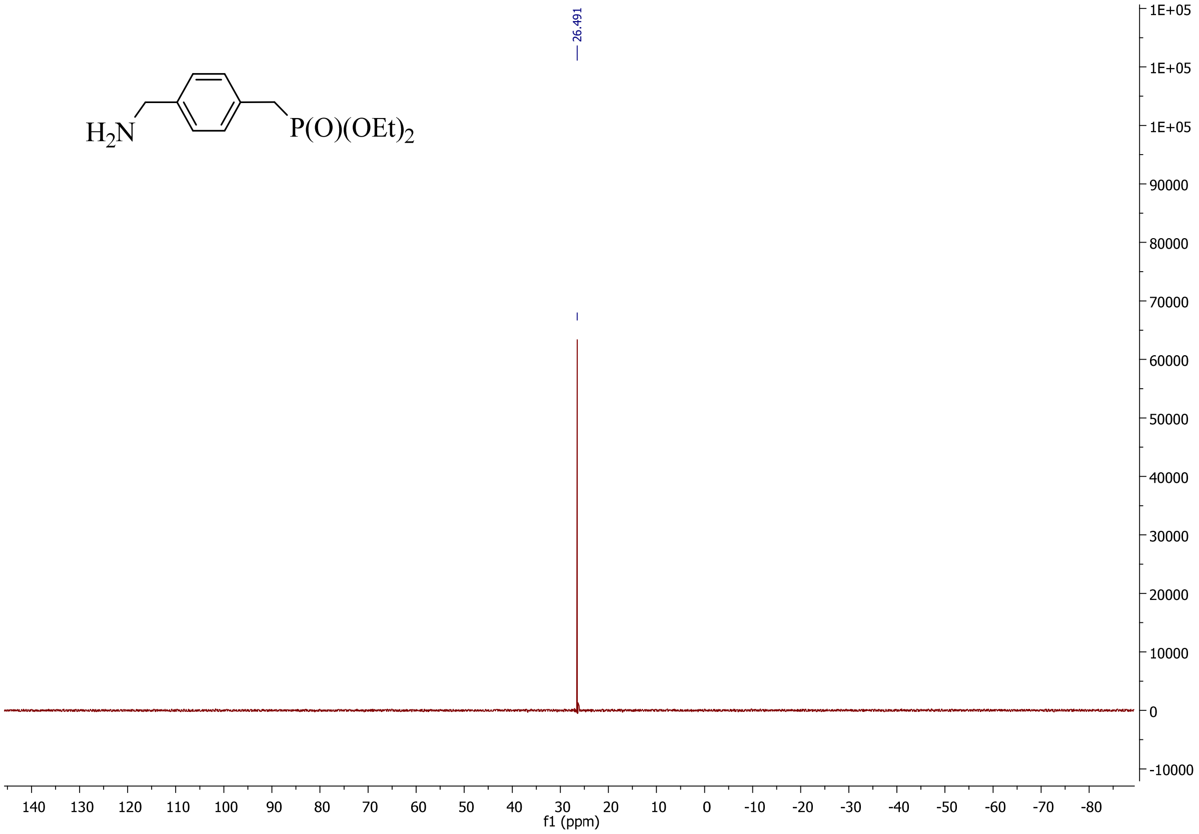

Supplementary Figure S28. 243 MHz $^{31}P\{H\}$NMR spectrum of **8c** in $CDCl_3$.

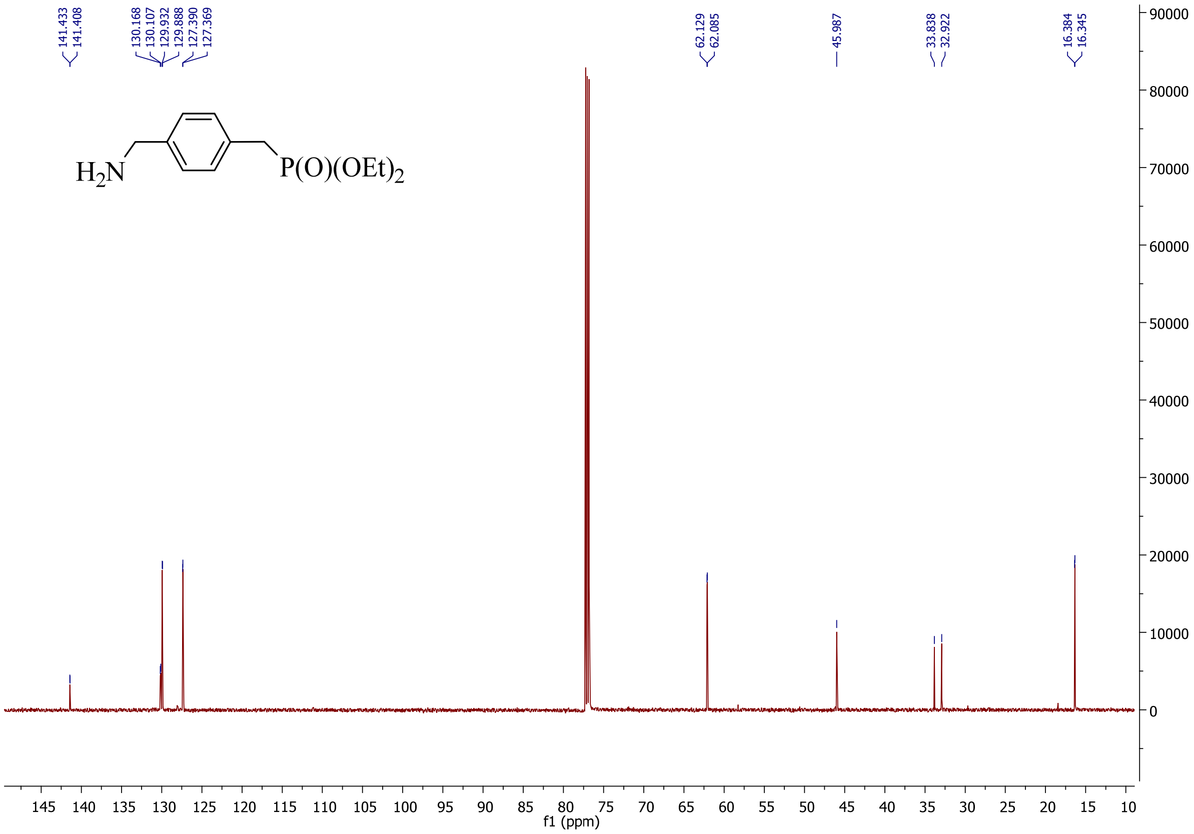

Supplementary Figure S29. 151 MHz ^{13}C NMR spectrum of **8c** in $CDCl_3$.

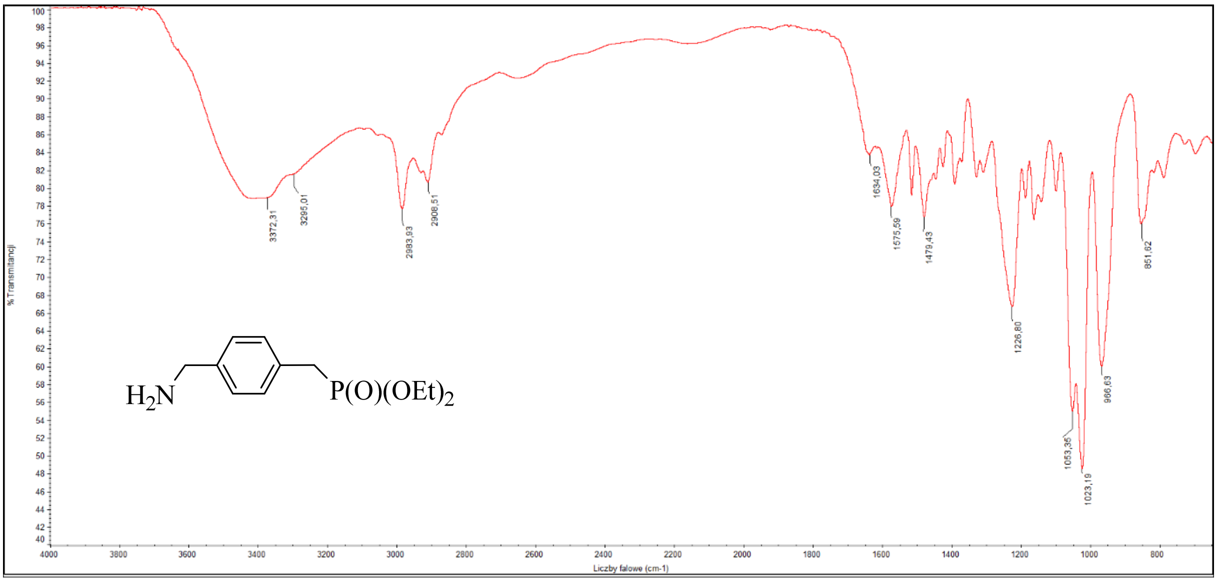

Supplementary Figure S30. ATR, IR spectrum of **8c**.

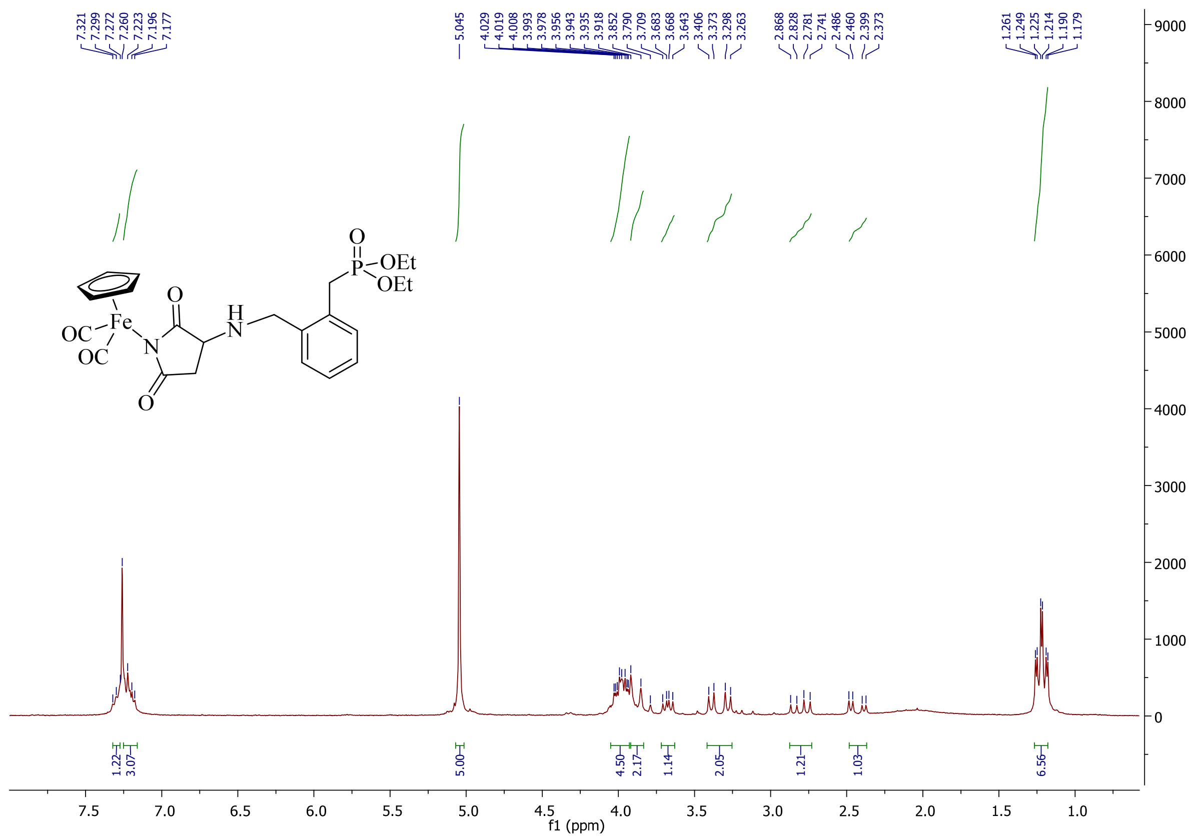

Supplementary Figure S31. 600 MHz ¹H NMR spectrum of **9a** in $CDCl_3$.

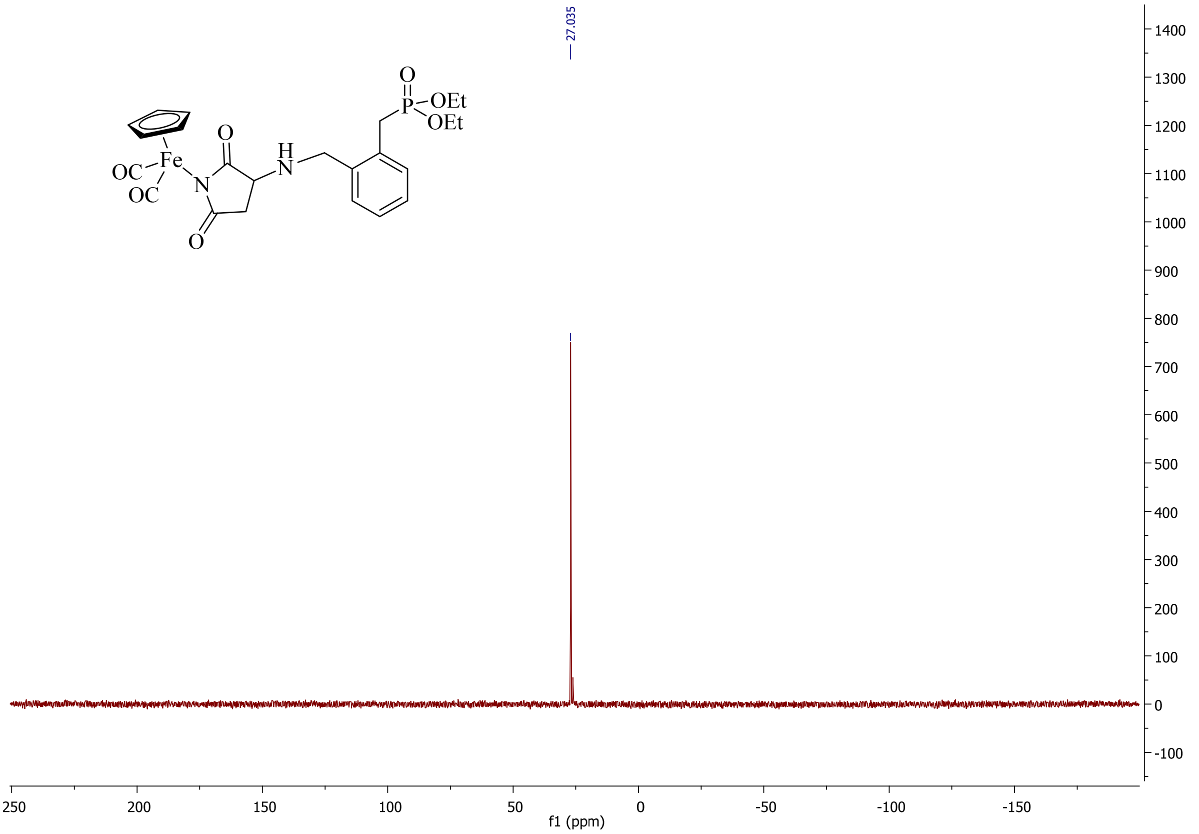

Supplementary Figure S32. 243 MHz ³¹P{H}NMR spectrum of **9a** in $CDCl_3$.

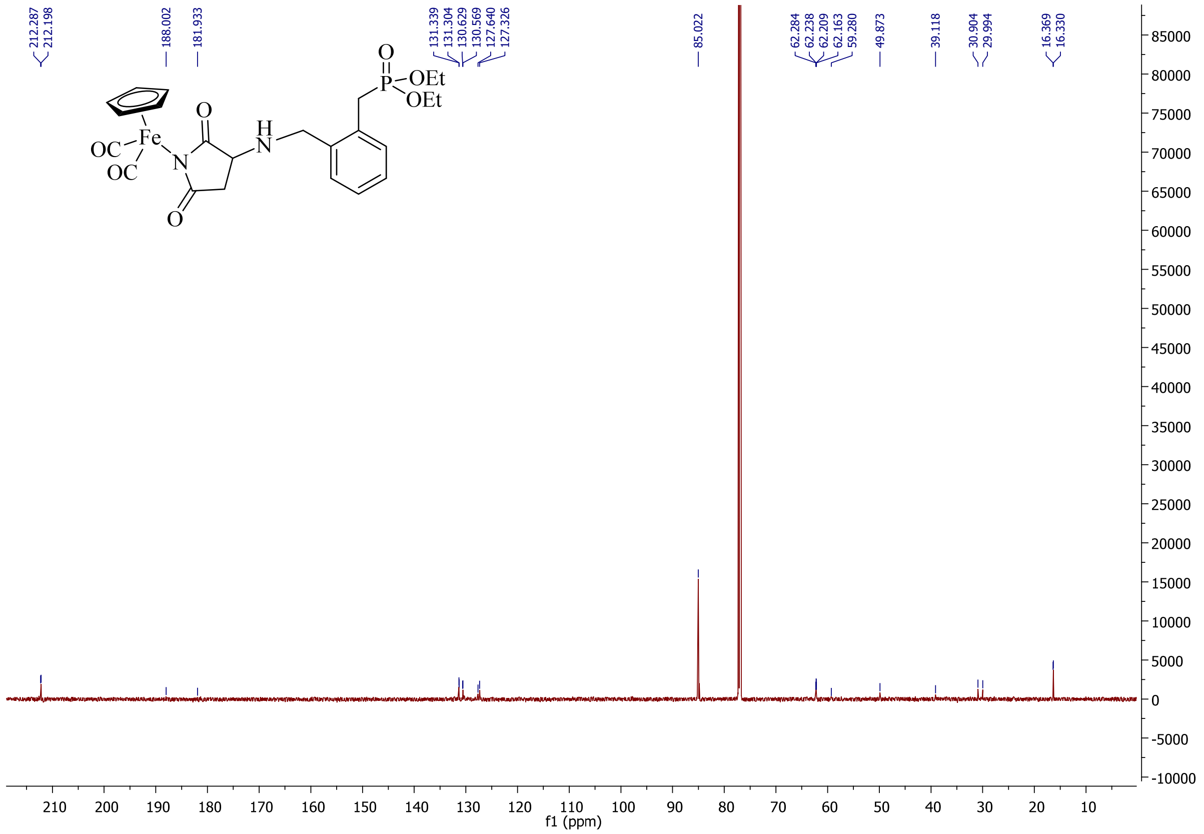

Supplementary Figure S33. 151 MHz ^{13}C NMR spectrum of **9a** in $CDCl_3$.

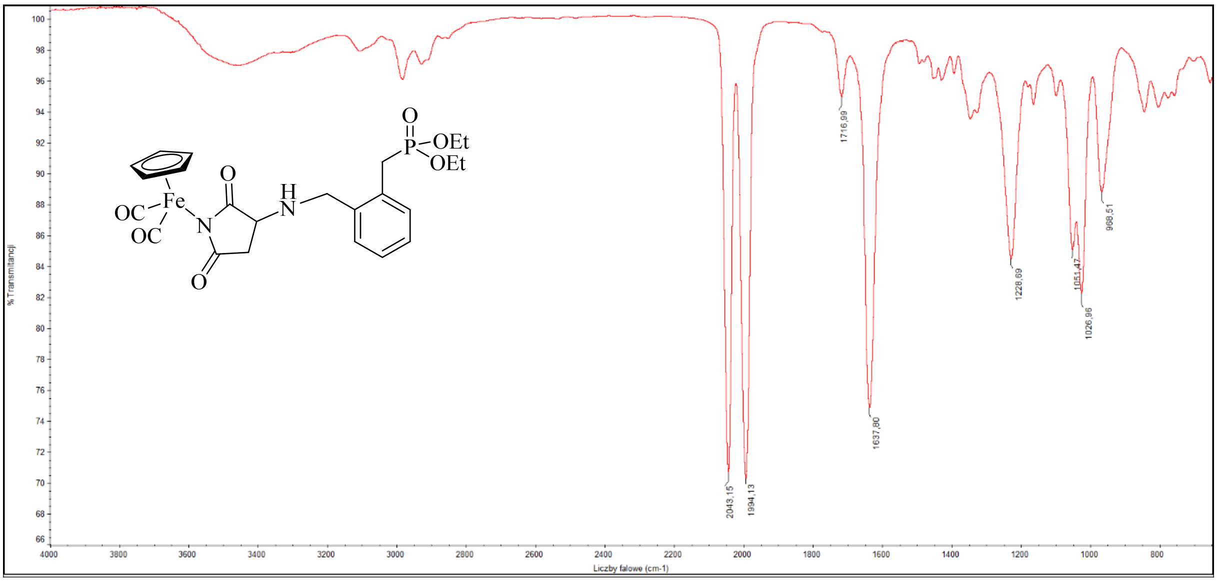

Supplementary Figure S34. ATR, IR spectrum of **9a**.

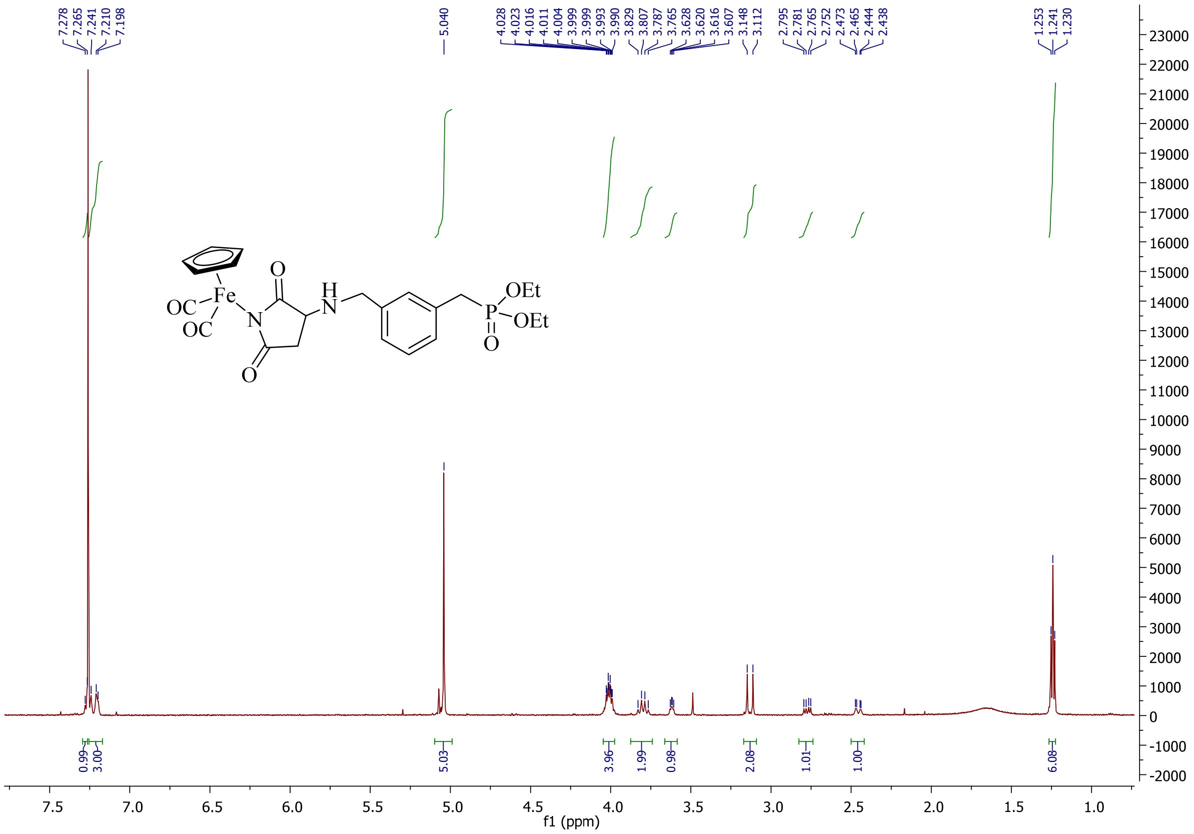

Supplementary Figure S35. 600 MHz ¹H NMR spectrum of 9b in CDCl3.

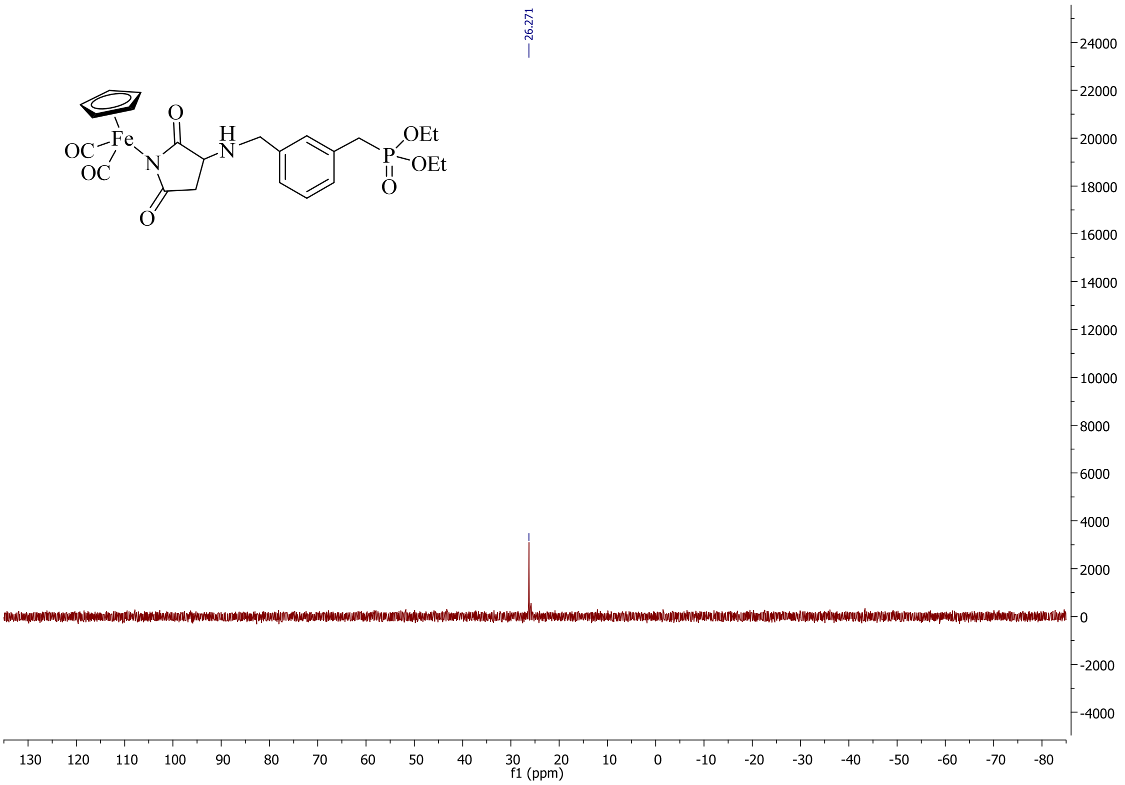

Supplementary Figure S36. 243 MHz ^{31}P{H}NMR spectrum of **9b** in $CDCl_3$.

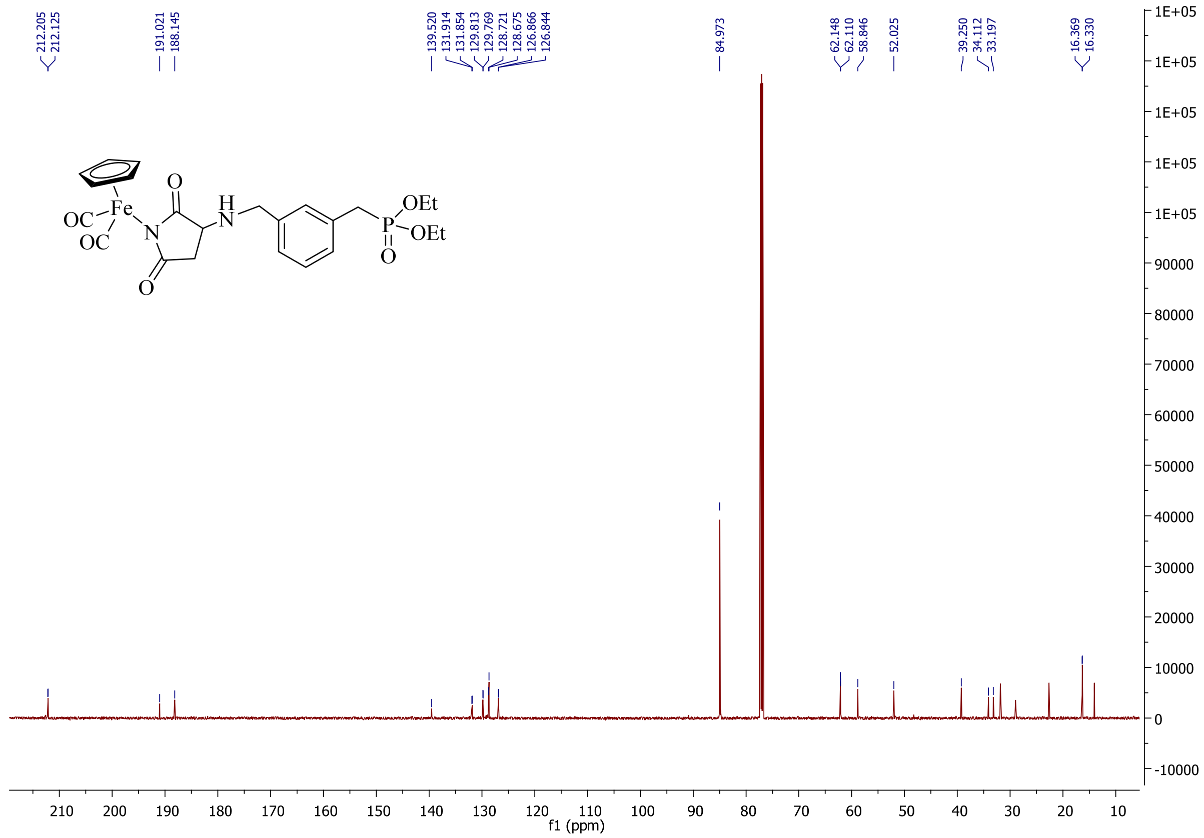

Supplementary Figure S37. 151 MHz ^{13}C NMR spectrum of **9b** in $CDCl_3$.

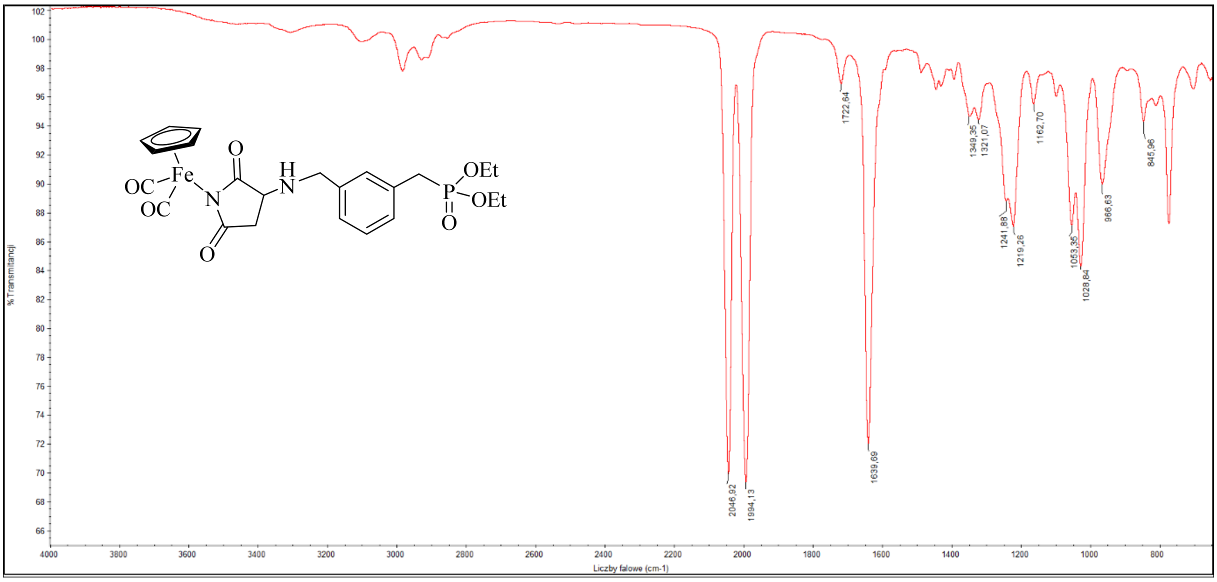

Supplementary Figure S38. ATR, IR spectrum of **9b**.

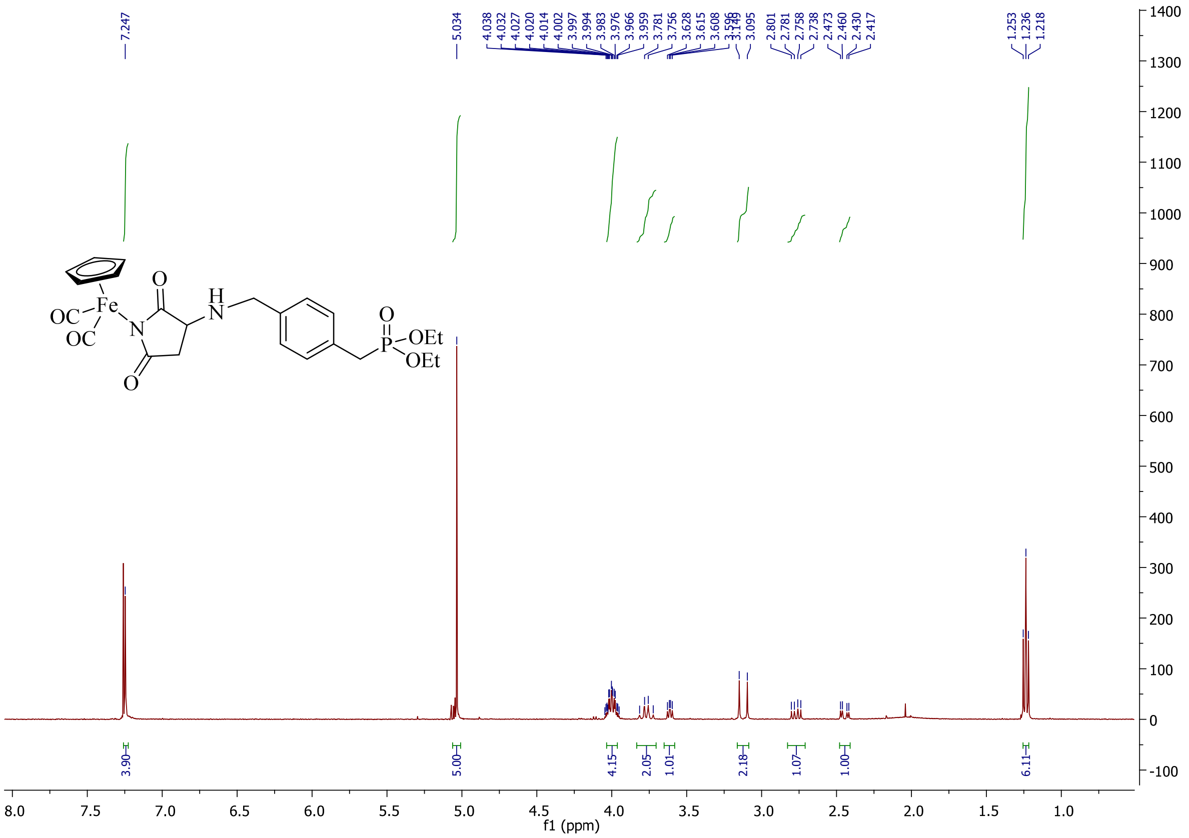

Supplementary Figure S39. 400 MHz ^{1}H NMR spectrum of **9c** in $CDCl_3$.

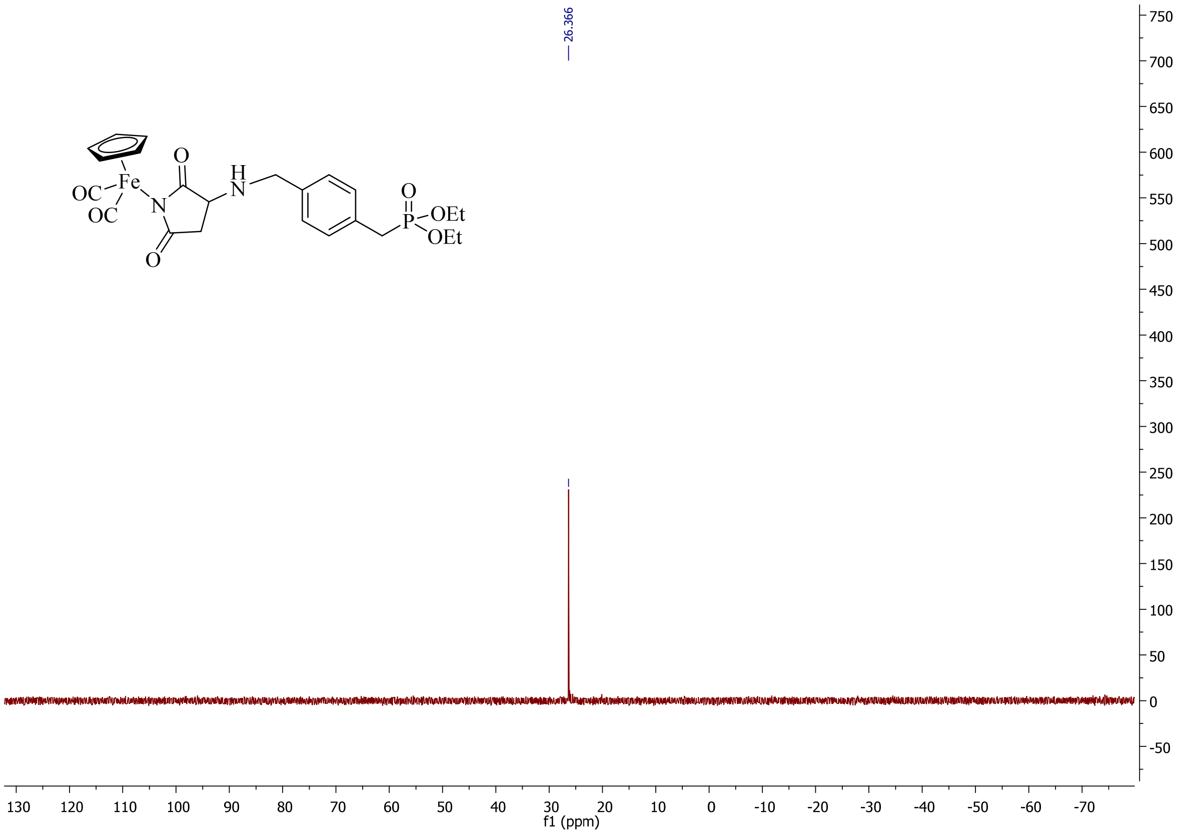

Supplementary Figure S40. 162 MHz $^{31}P\{H\}$NMR spectrum of **9c** in $CDCl_3$.

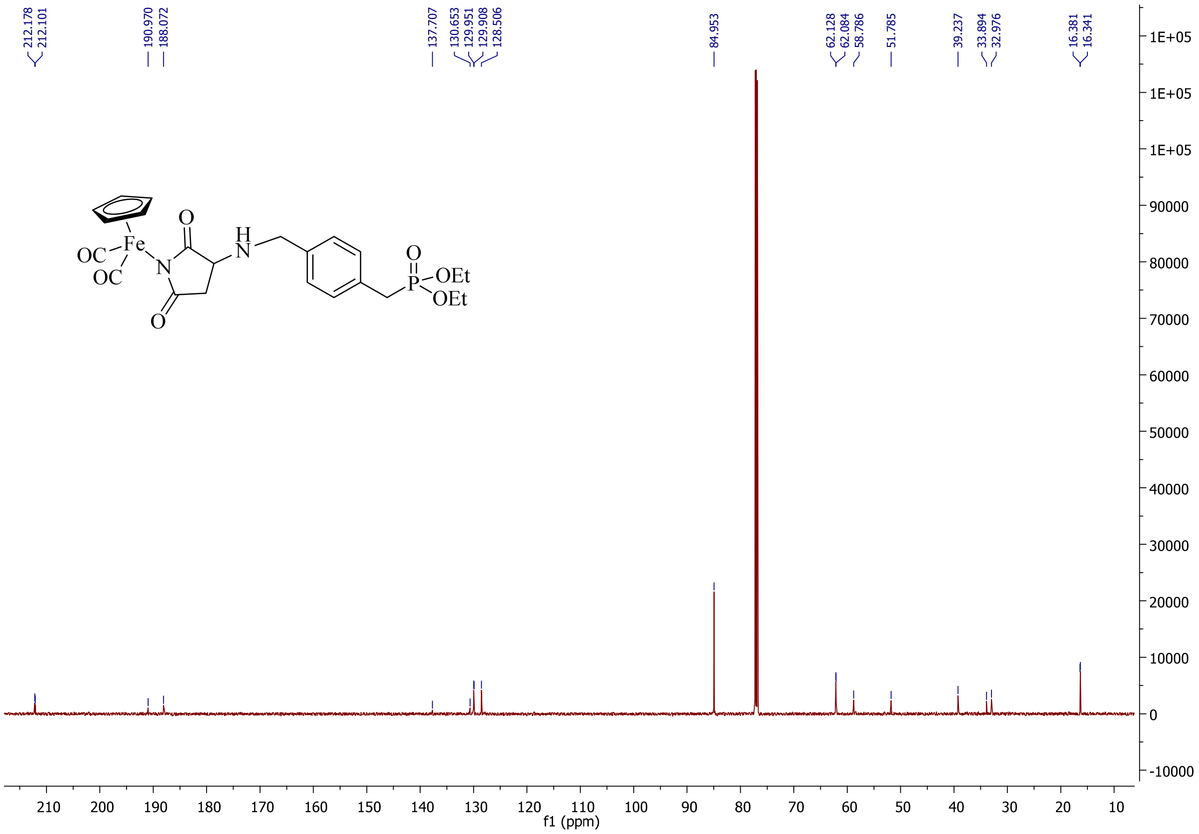

Supplementary Figure S41. 151 MHz ^{13}C NMR spectrum of **9c** in $CDCl_3$.

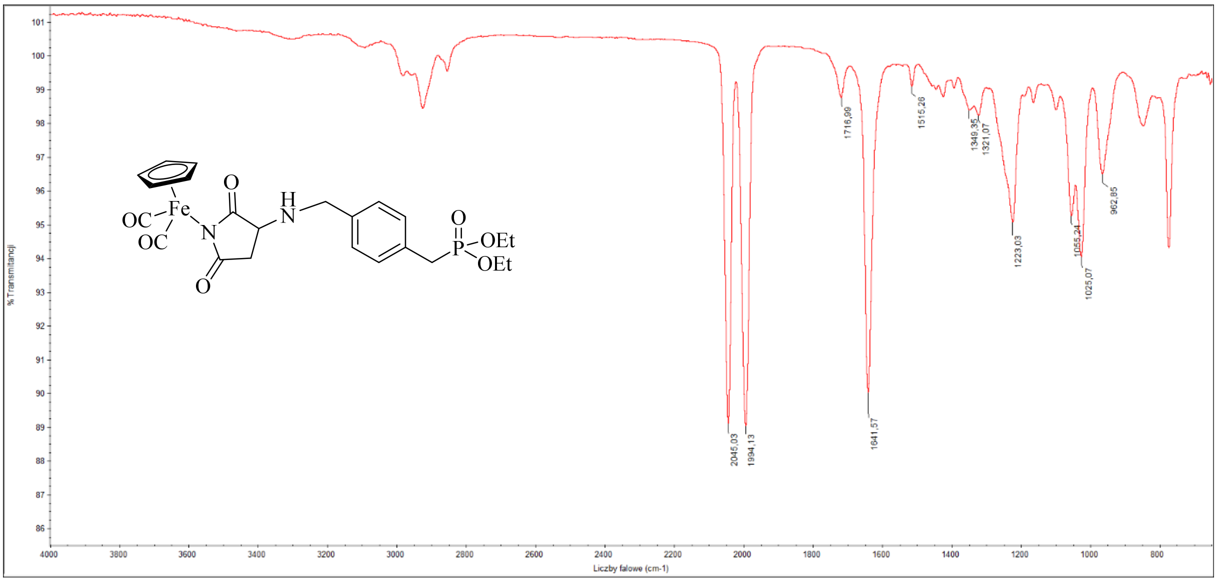

Supplementary Figure S42. ATR, IR spectrum of **9c**.

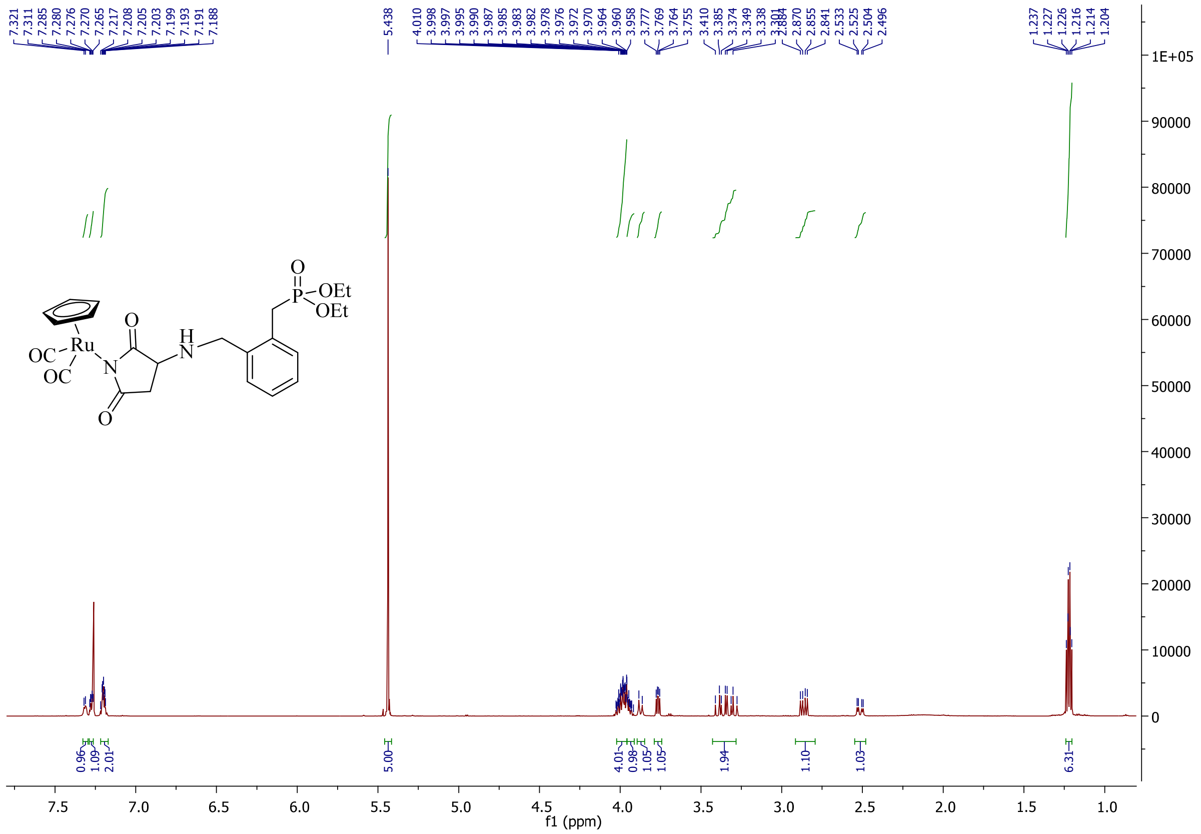

Supplementary Figure S43. 600 MHz ^{1}H NMR spectrum of **10a** in $CDCl_3$.

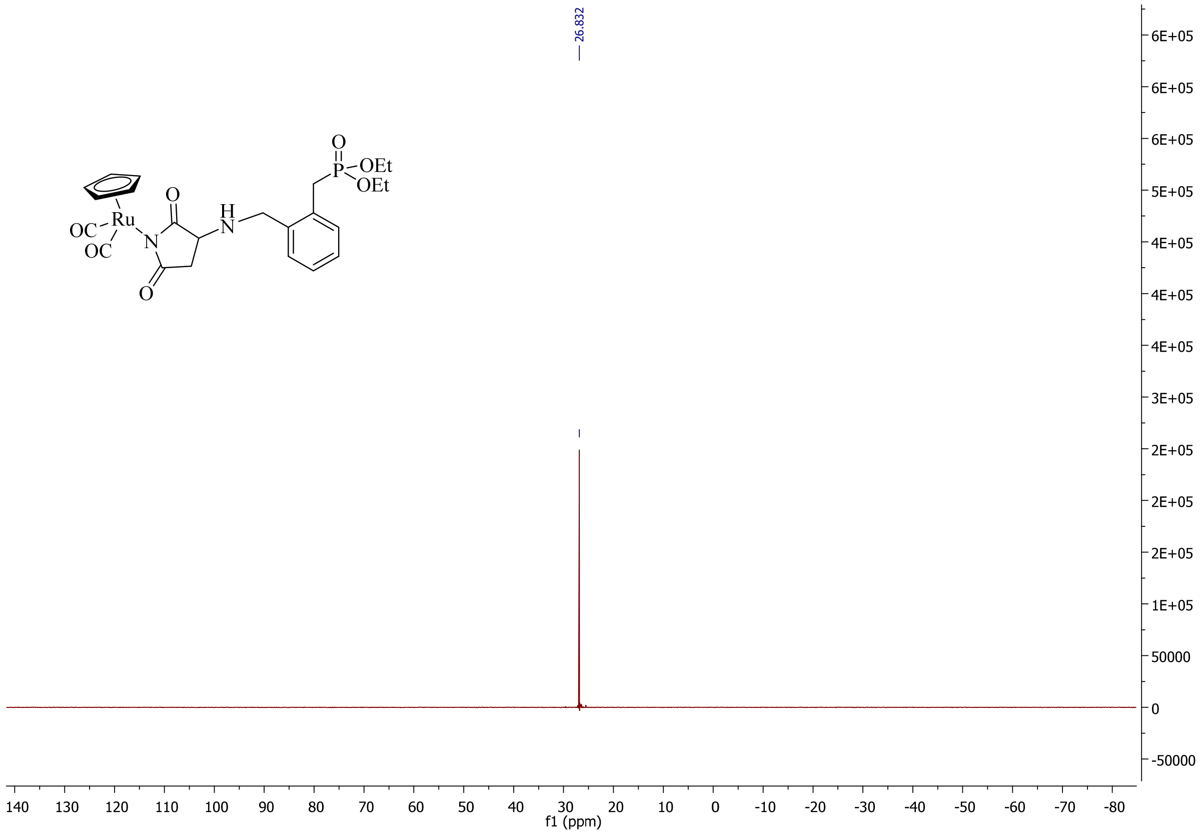

Supplementary Figure S44. 243 MHz ^{31}P{H}NMR spectrum of **10a** in $CDCl_3$.

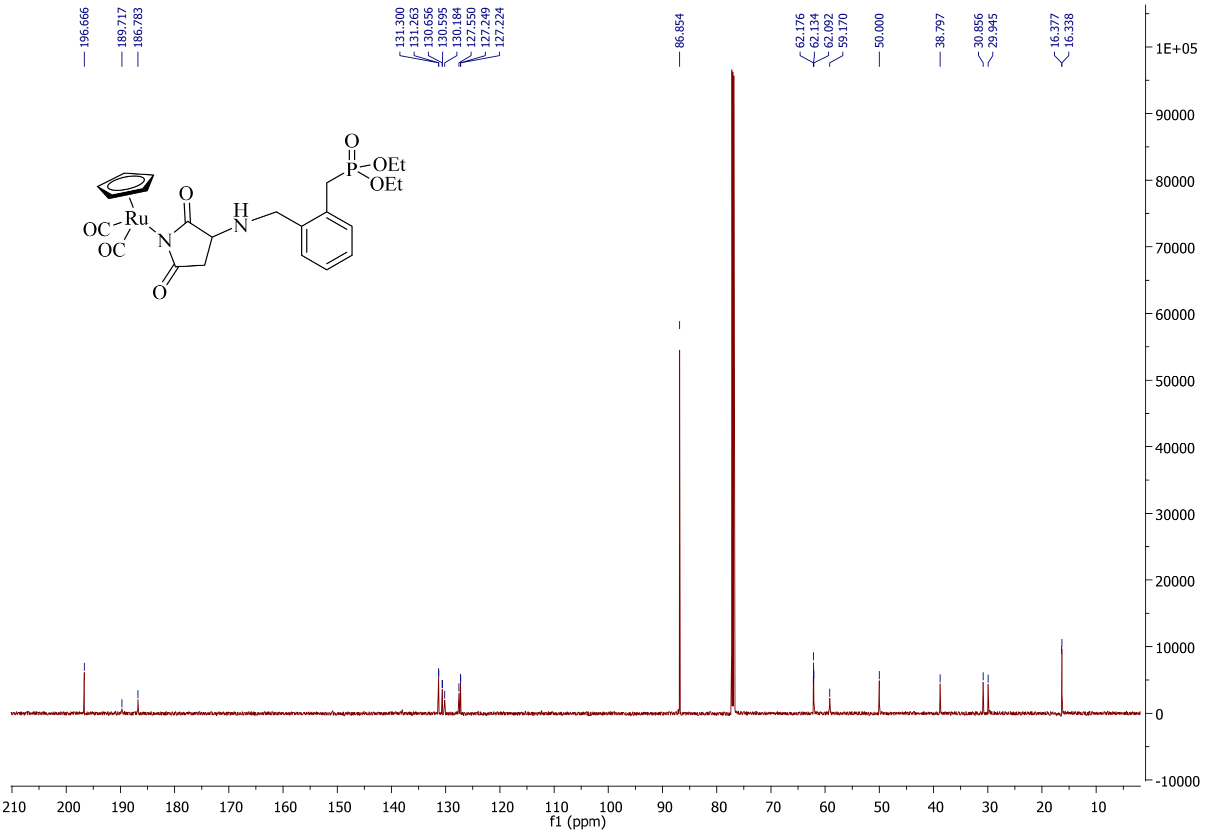

Supplementary Figure S45. 151 MHz ^{13}C NMR spectrum of **10a** in $CDCl_3$.

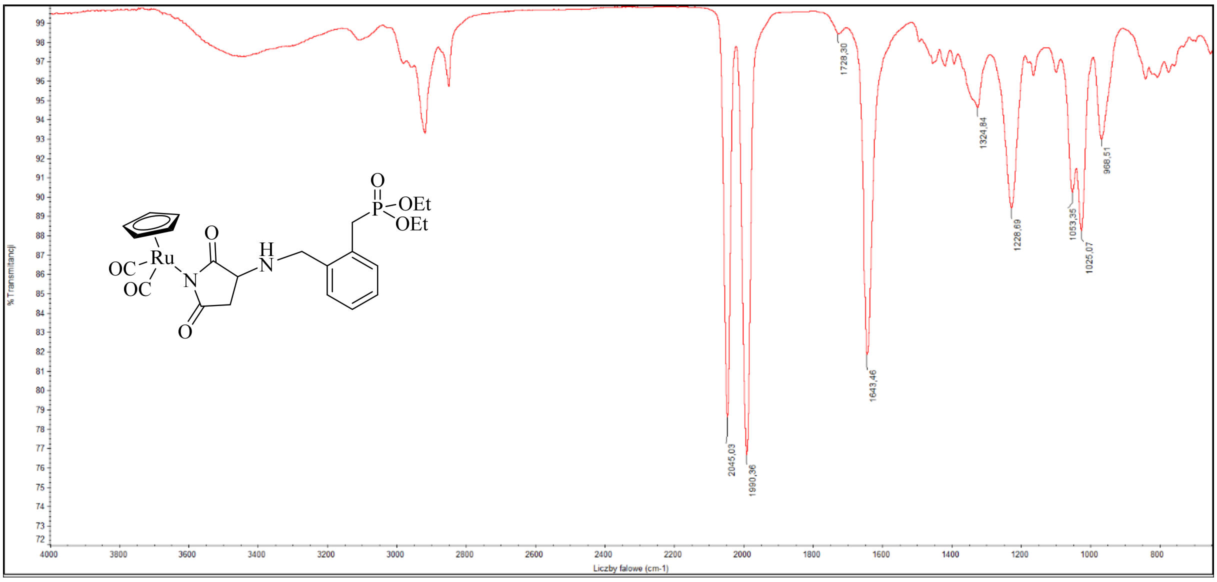

Supplementary Figure S46. ATR, IR spectrum of **10a**.

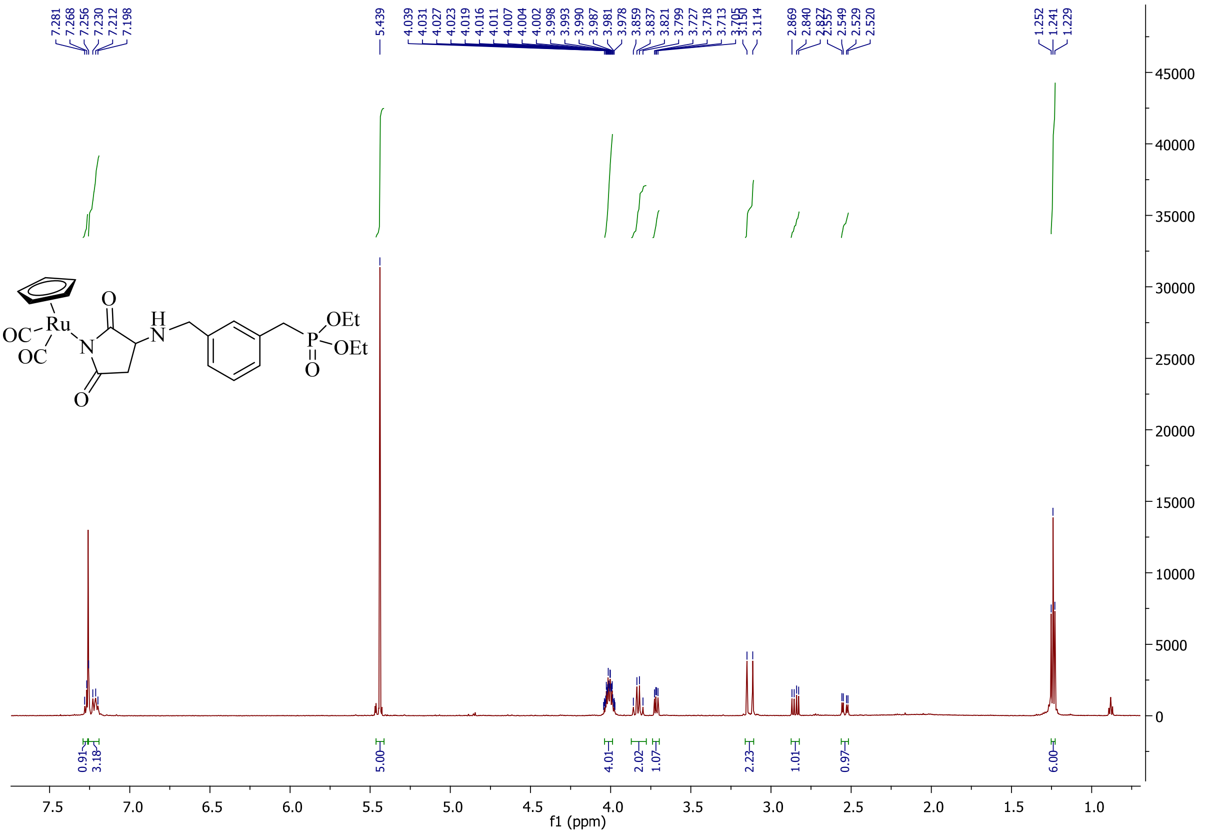

Supplementary Figure S47. 600 MHz ^{1}H NMR spectrum of **10b** in $CDCl_3$.

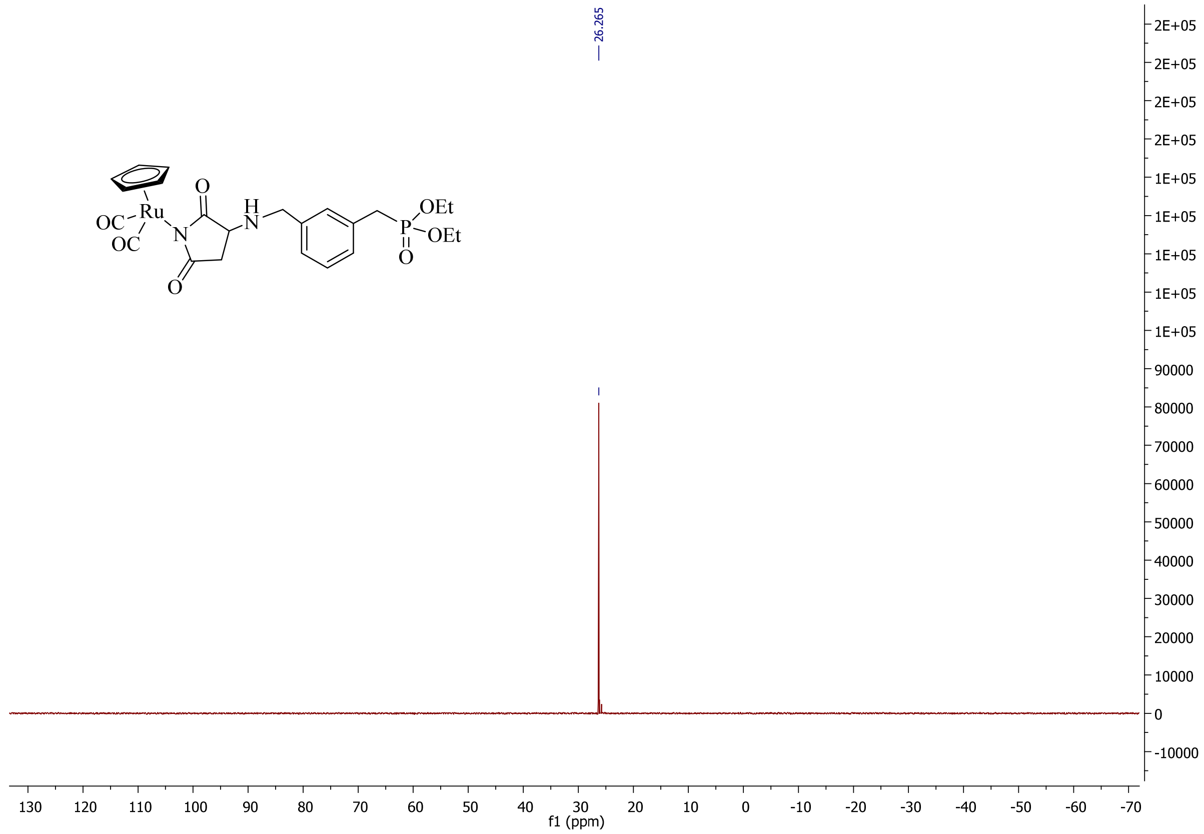

Supplementary Figure S48. 243 MHz $^{31}P\{H\}$NMR spectrum of **10b** in $CDCl_3$.

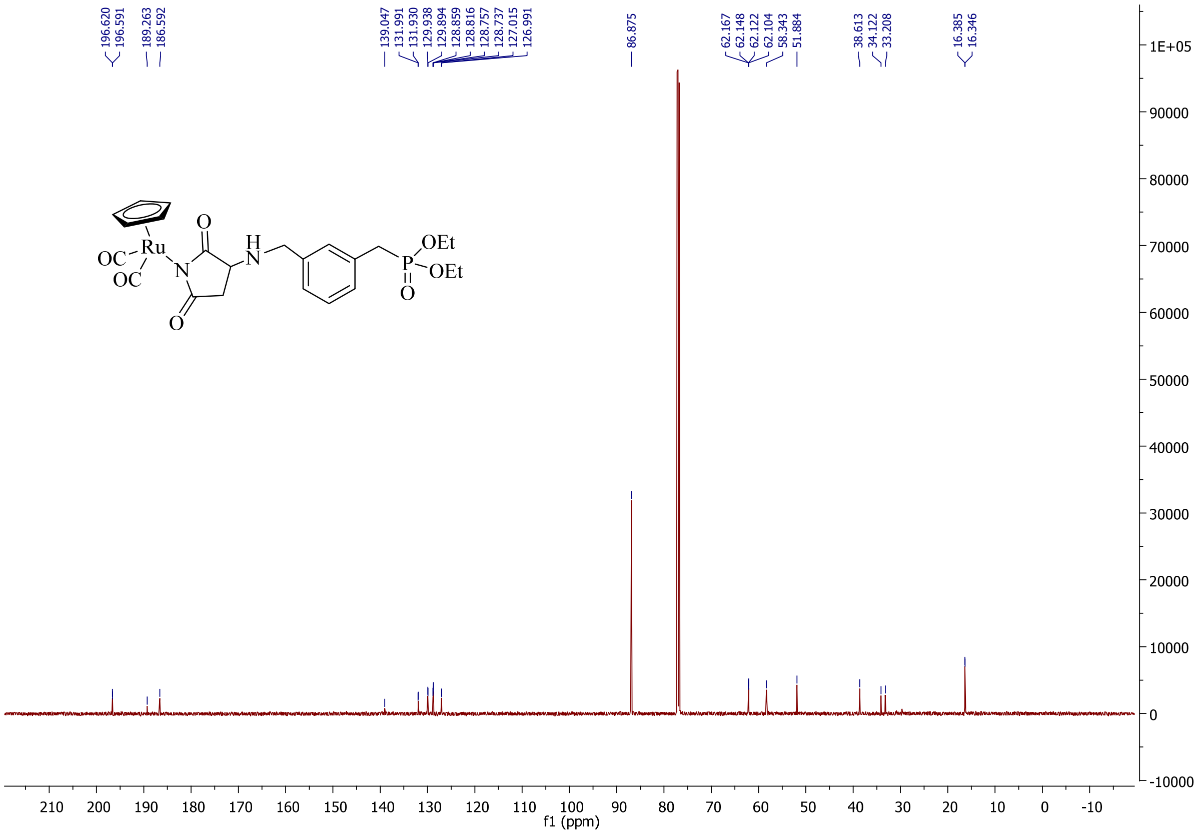

Supplementary Figure S49. 151 MHz ^{13}C NMR spectrum of **10b** in $CDCl_3$.

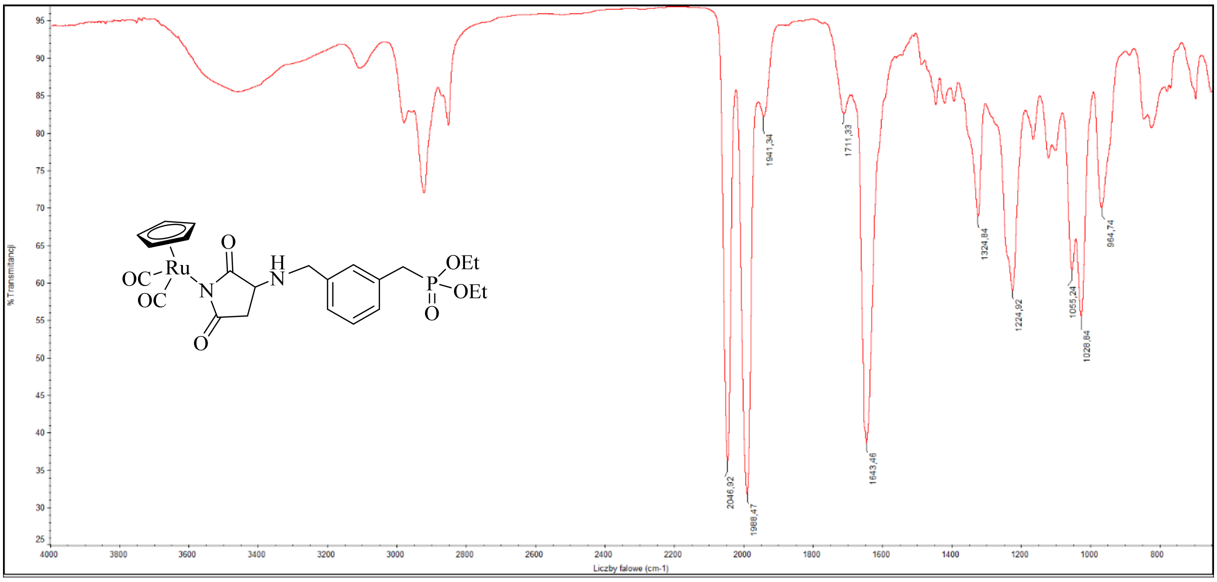

Supplementary Figure S50. ATR, IR spectrum of **10b**.

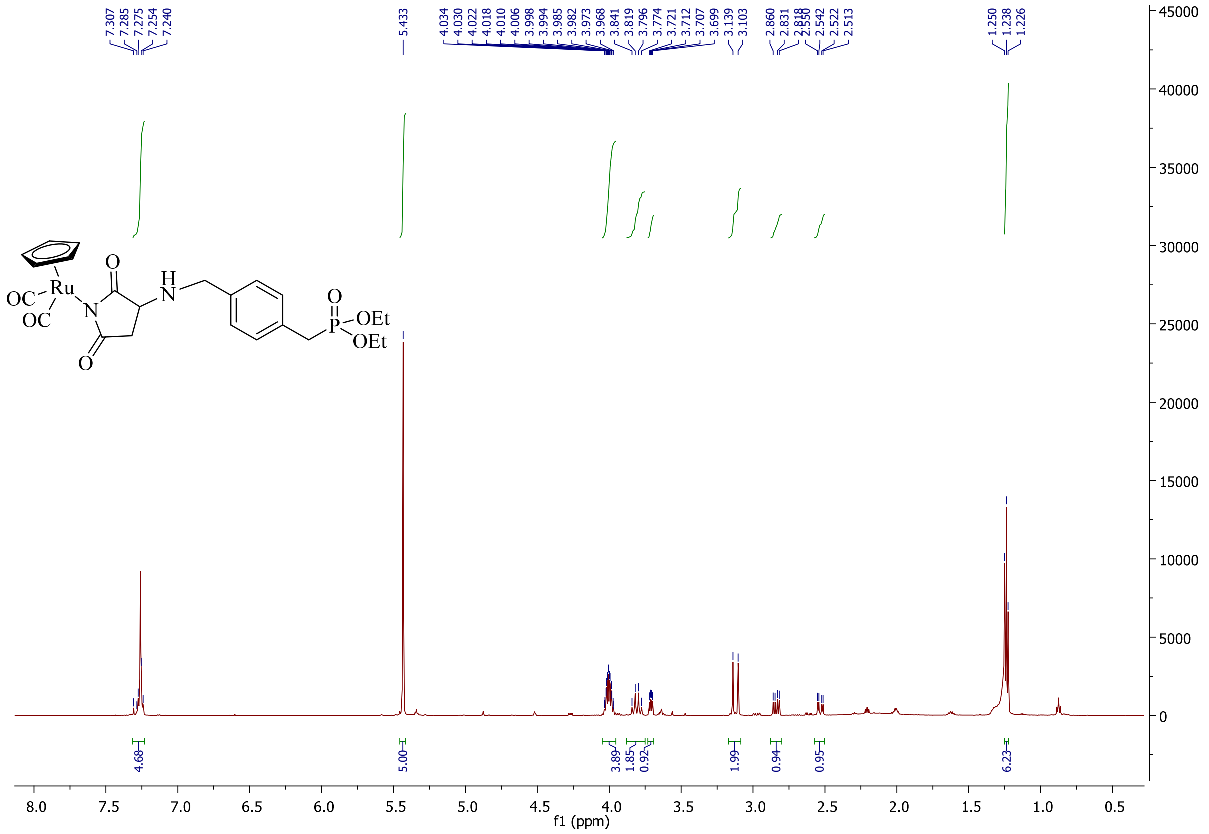

Supplementary Figure S51. 600 MHz ^{1}H NMR spectrum of **10c** in $CDCl_3$.

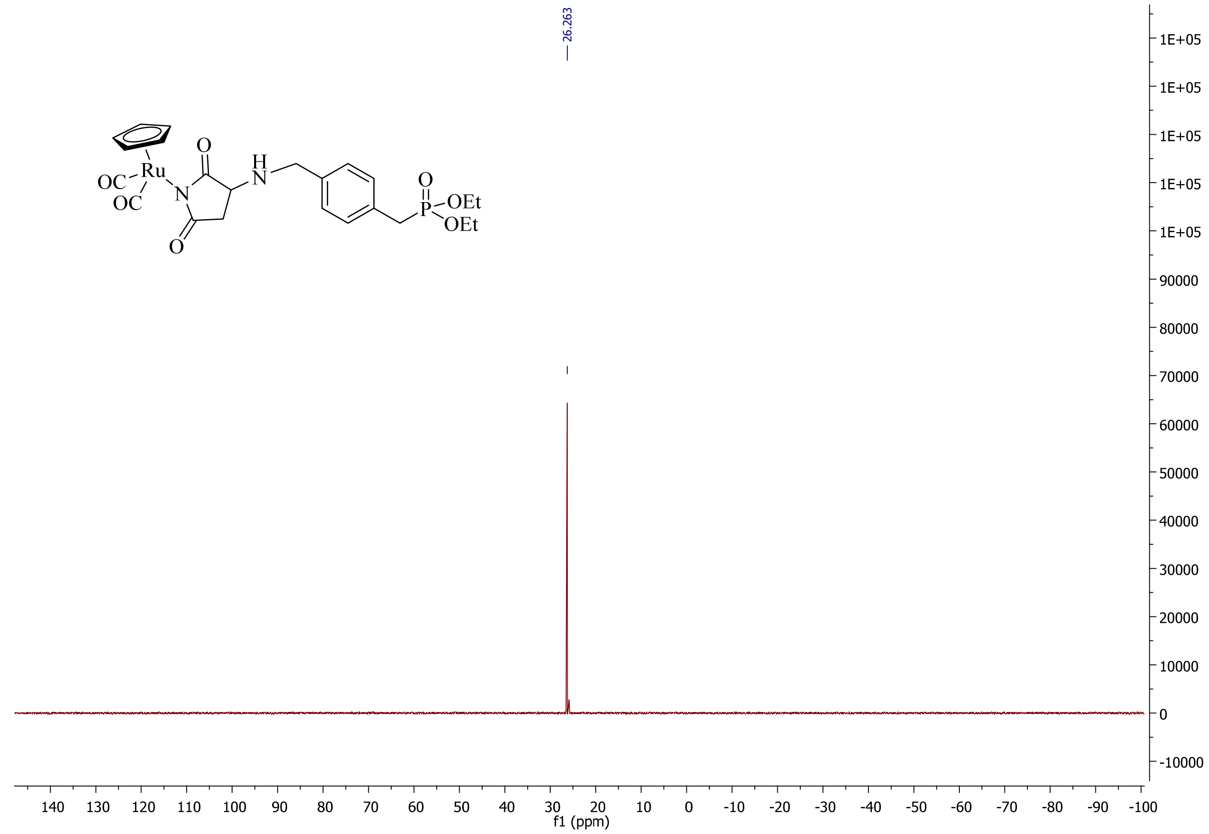

Supplementary Figure S52. 243 MHz ^{31}P{H}NMR spectrum of **10c** in $CDCl_3$.

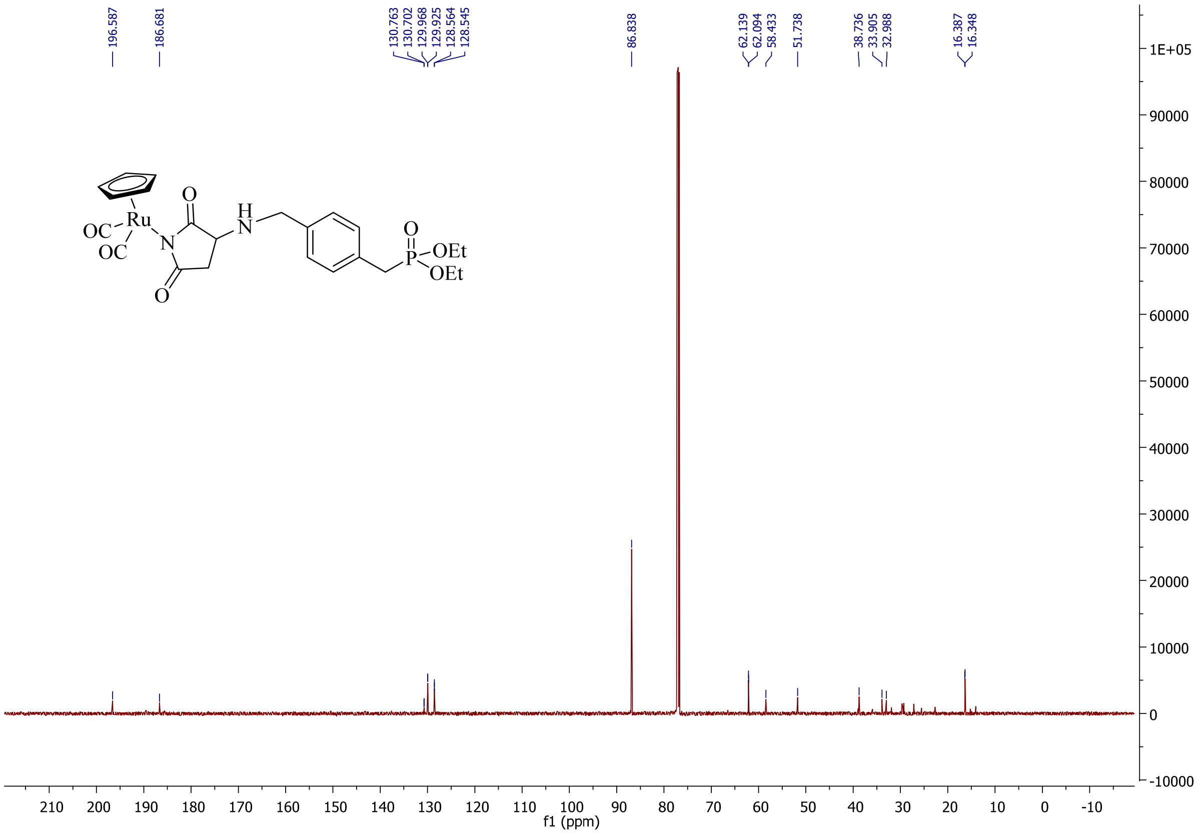

Supplementary Figure S53. 151 MHz ^{13}C NMR spectrum of **10c** in $CDCl_3$.

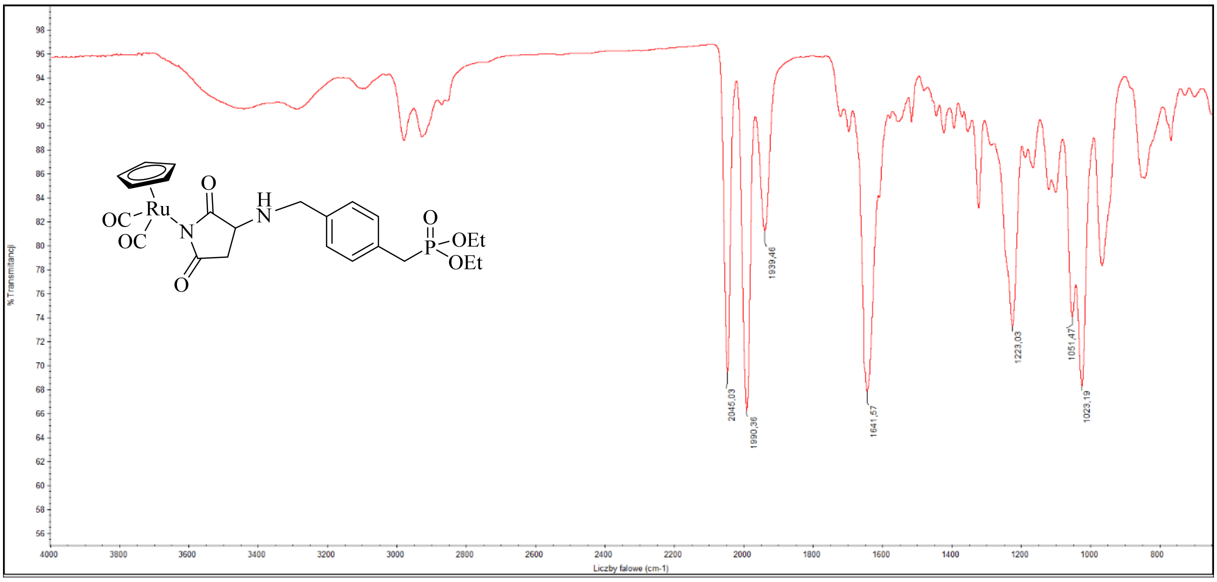

Supplementary Figure S54. ATR, IR spectrum of **10c**.

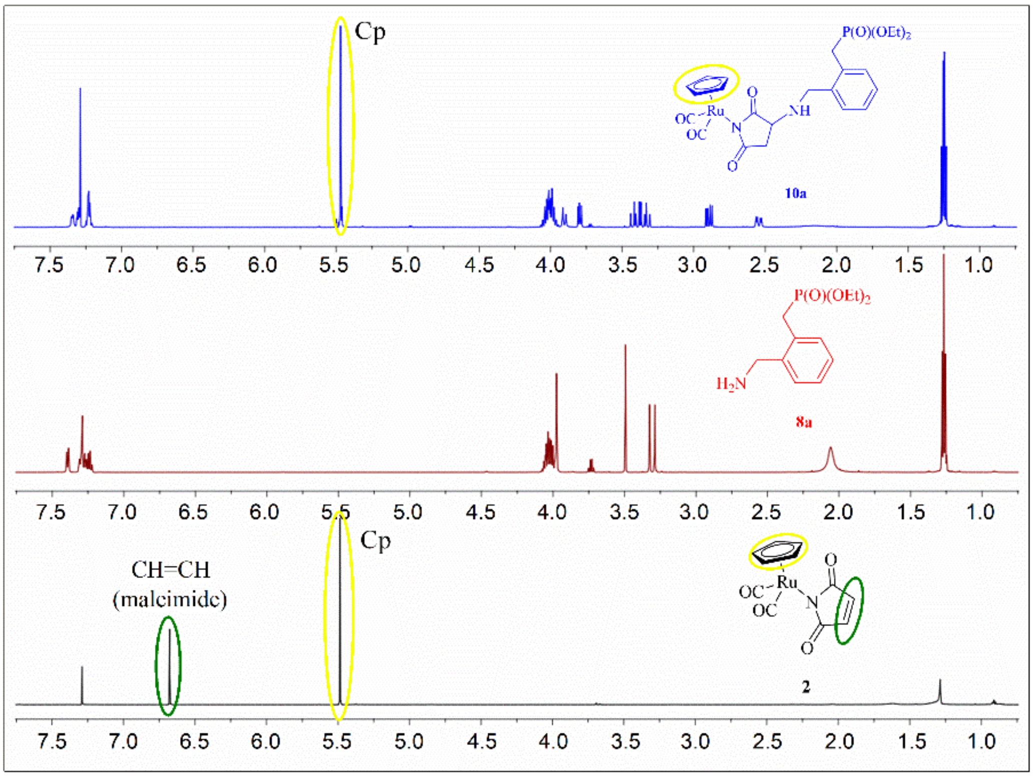

Supplementary Figure S55. ^{1}H-NMR spectra of compounds 10a, 8a and 2.

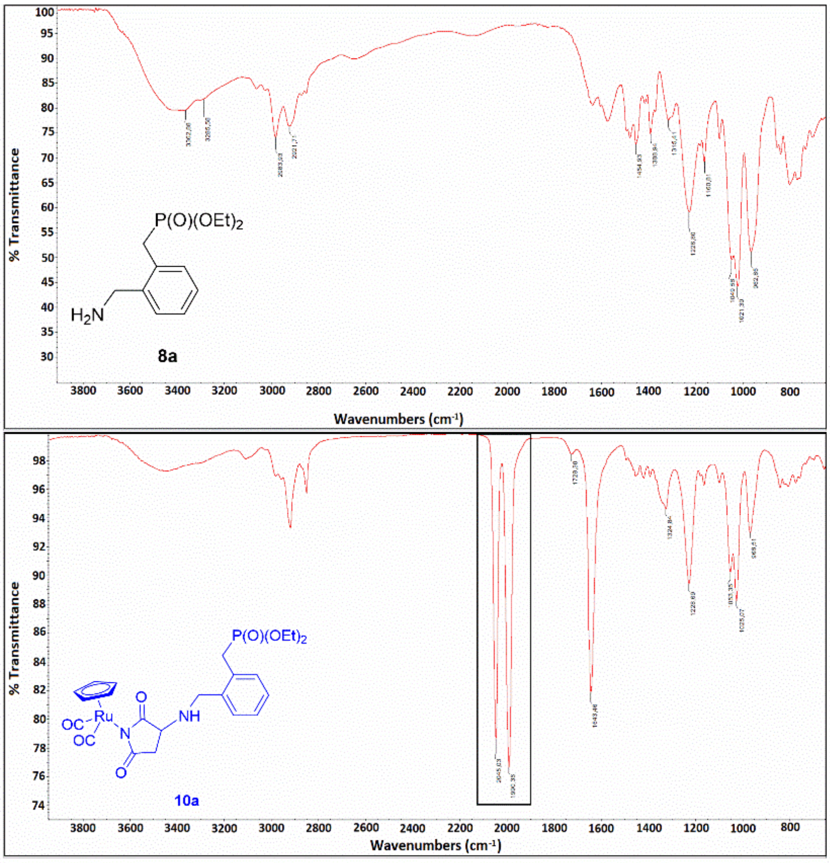

Supplementary Figure S56. IR spectra of the compounds 8a and 10a (Ru).

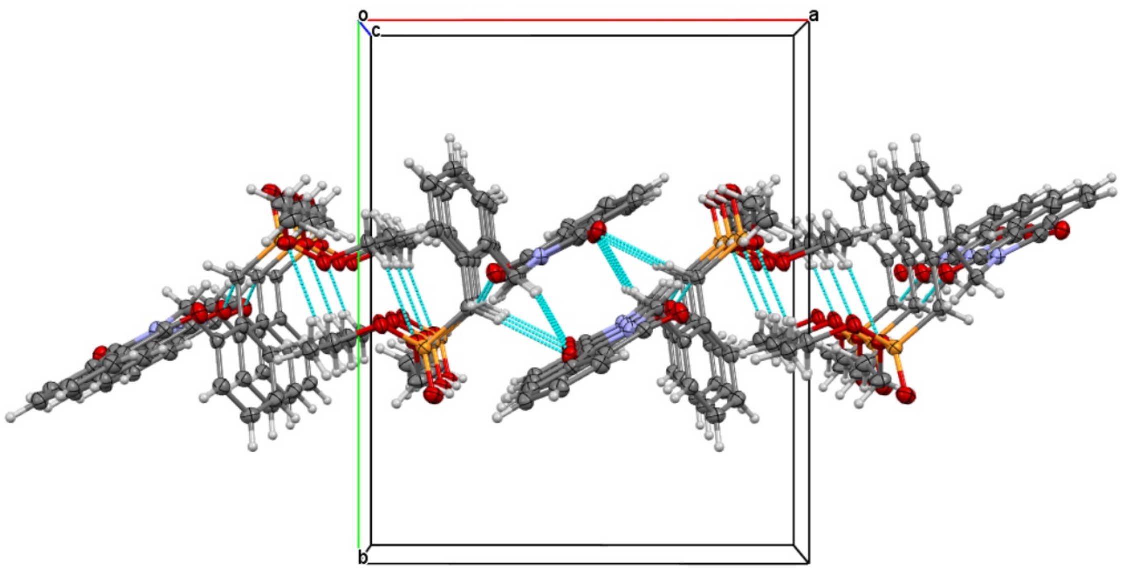

Supplementary Figure S57. Scheme of non-covalent interactions in the crystal structures of **7a** C-H···O hydrogen bonds - blue dotted lines.

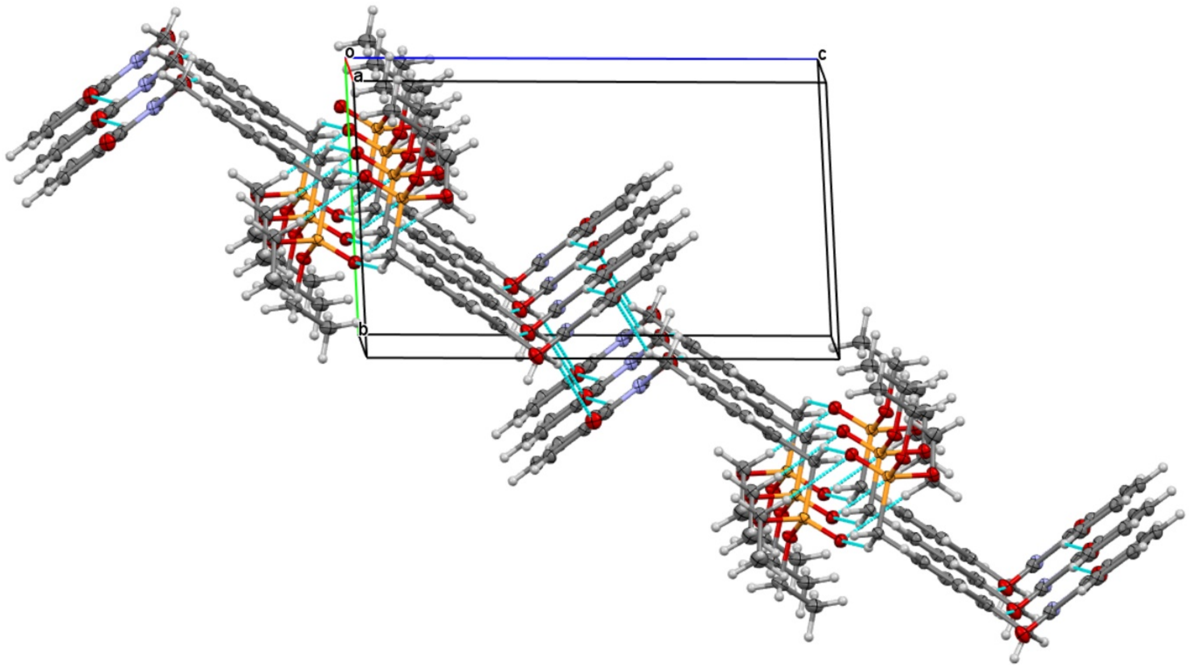

Supplementary Figure S58. Scheme of non-covalent interactions in the crystal structures of **7c** C-H···O hydrogen bonds - blue dotted lines.

Supplementary Table S4. Crystal data and structure refinement.

**Identification code**	**7a**	**7c**
Empirical formula	$C_{20}H_{22}NO_5P$	$C_{20}H_{22}NO_5P$
Formula weight	387.35	387.35
Crystal system	monoclinic	triclinic
Space group	$P2_1/c$	P-1
a	13.70871(16) Å	7.98632(18) Å
b	16.52591(17) Å	8.94794(19) Å
c	8.44116(8) Å	14.7214(3) Å
	90°	83.8092(18)°
	90.4865(10)°	77.3761(19)°
	90°	66.294(2)°
Volume	1912.27(4) $Å^3$	939.72(4) $Å^3$
Z	4	8
ϱ_{calc}	1.345 g/cm^3	1.369 g/cm^3
μ	1.545 mm^{-1}	1.572 mm^{-1}
F(000)	816	408
2Θ range for data collection	6.448° – 153.372°	6.154° – 152.936°
Index ranges	-17 ≤ h ≤ 14,<br>-20 ≤ k ≤ 20,<br>-10 ≤ l ≤ 10	-10 ≤ h ≤ 9,<br>-11 ≤ k ≤ 11,<br>-18 ≤ l ≤ 18
Reflections collected / independent	67563/4016<br>[R_{int} = 0.0768, R_{sigma} = 0.0196]	37694/3920<br>[R_{int} = 0.0283, R_{sigma} = 0.0109]
Data/restraints/parameters	4016/0/246	3920/0/246
Goodness-of-fit on F^2	1.022	1.045
Final R indexes [I>=2σ (I)]	R_1 = 0.0380, wR_2 = 0.1014	R_1 = 0.0315, wR_2 = 0.0831
Final R indexes [all data]	R_1 = 0.0408, wR_2 = 0.1048	R_1 = 0.0320, wR_2 = 0.0834
Largest diff. peak/hole	0.34/-0.36 e $Å^{-3}$	0.38/-0.45 e $Å^{-3}$
CCDC no.	2154277	2154278

Supplementary Table S5. Hydrogen bonding geometry [Å, deg].

D	H	A	d(D-H)	d(H…A)	D-H…A	symmetry
**7a**						
C10	H10A	O26	0.99	2.454	159.99	1-x,1-y,1-z
C17	H17B	O26	0.99	2.374	164.77	1-x,1-y,1-z
C17	H17A	O27	0.99	2.354	157.79	x,y,-1+z
C20	H20B	O22	0.99	2.581	156.70	-x,1-y,1-z
**7c**						
C4	H4	O27	0.94	2.595	126.78	-1+x,y,z
C10	H10B	O27	0.97	2.527	153.90	-x,2-y,1-z
C17	H17B	O25	0.97	2.559	149.36	-x,1-y,-z
C20	H20A	O25	0.97	2.451	171.78	-x,1-y,-z
C12	H12	O26	0.93	2.577	134.80	-1+x,y,z

Supplementary Figure S59. Structural alignment of selected AChEs: human hAChE (1B41) - blue; optimized human hAChE - green; electric eel eeAChE (1C2B) - magenta; and reconstructed whole eeAChE - yellow.

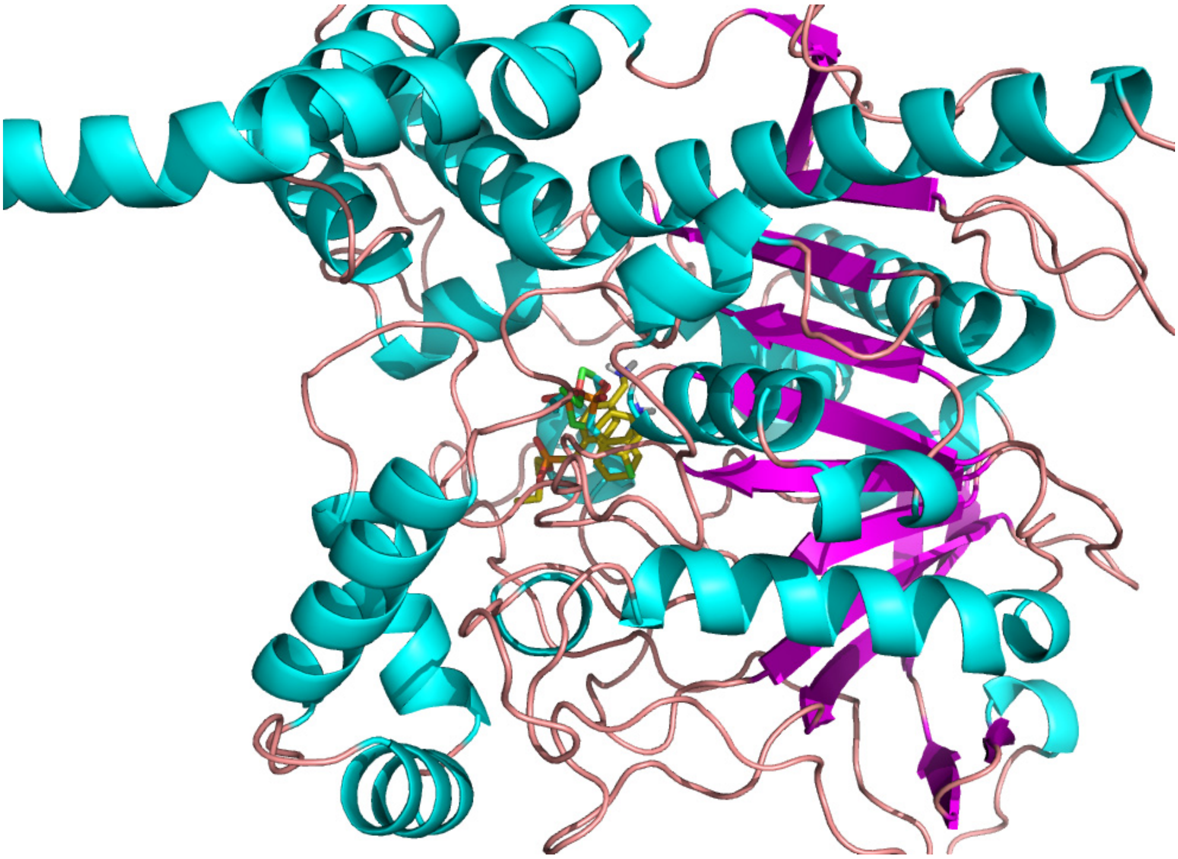

Supplementary Figure S60. Structure representation of the eeAChE enzyme from electric eel (based on the structure from pdb code 1C2B) with all 3 ligands (**8a-c**) in the active side.

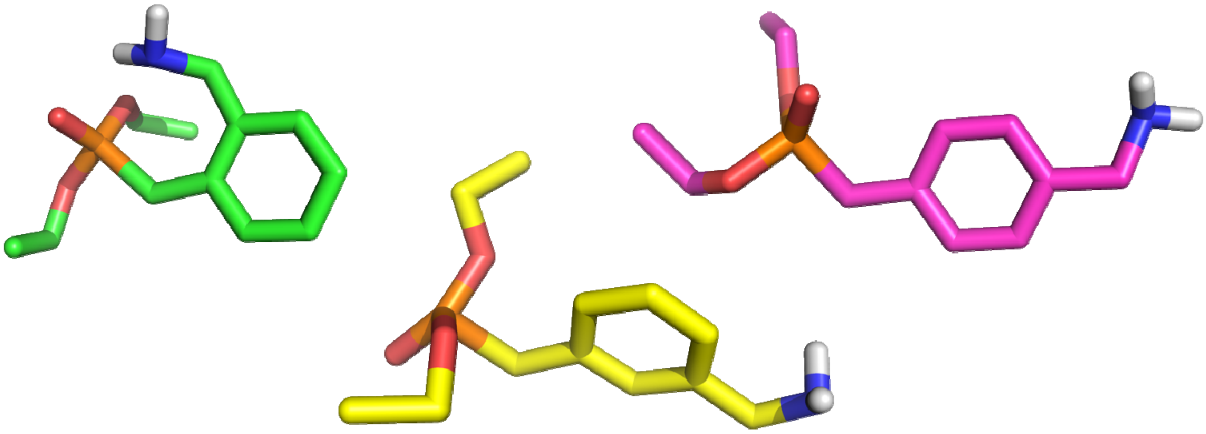

Supplementary Figure S61. Computer-generated (UCSF Chimera) 3D structure of selected ligands **8a** - left, **8b** - center, **8c** - right.

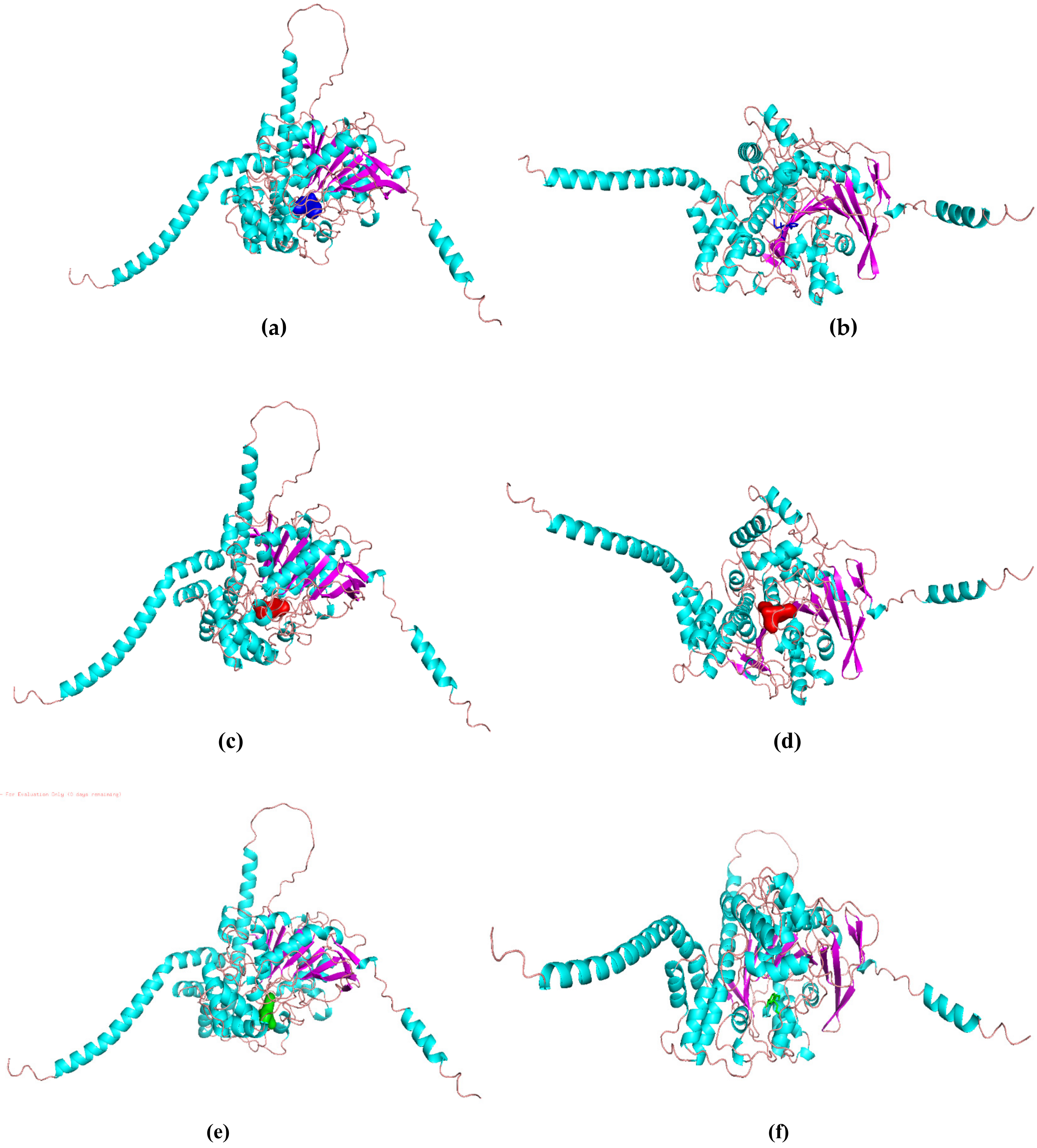

Supplementary Figure S62. Molecular docking structure of eeAChE and a ligand for molecule **8a** (a and b), **8b** (c and d) and **8c** (e and f) respectively.

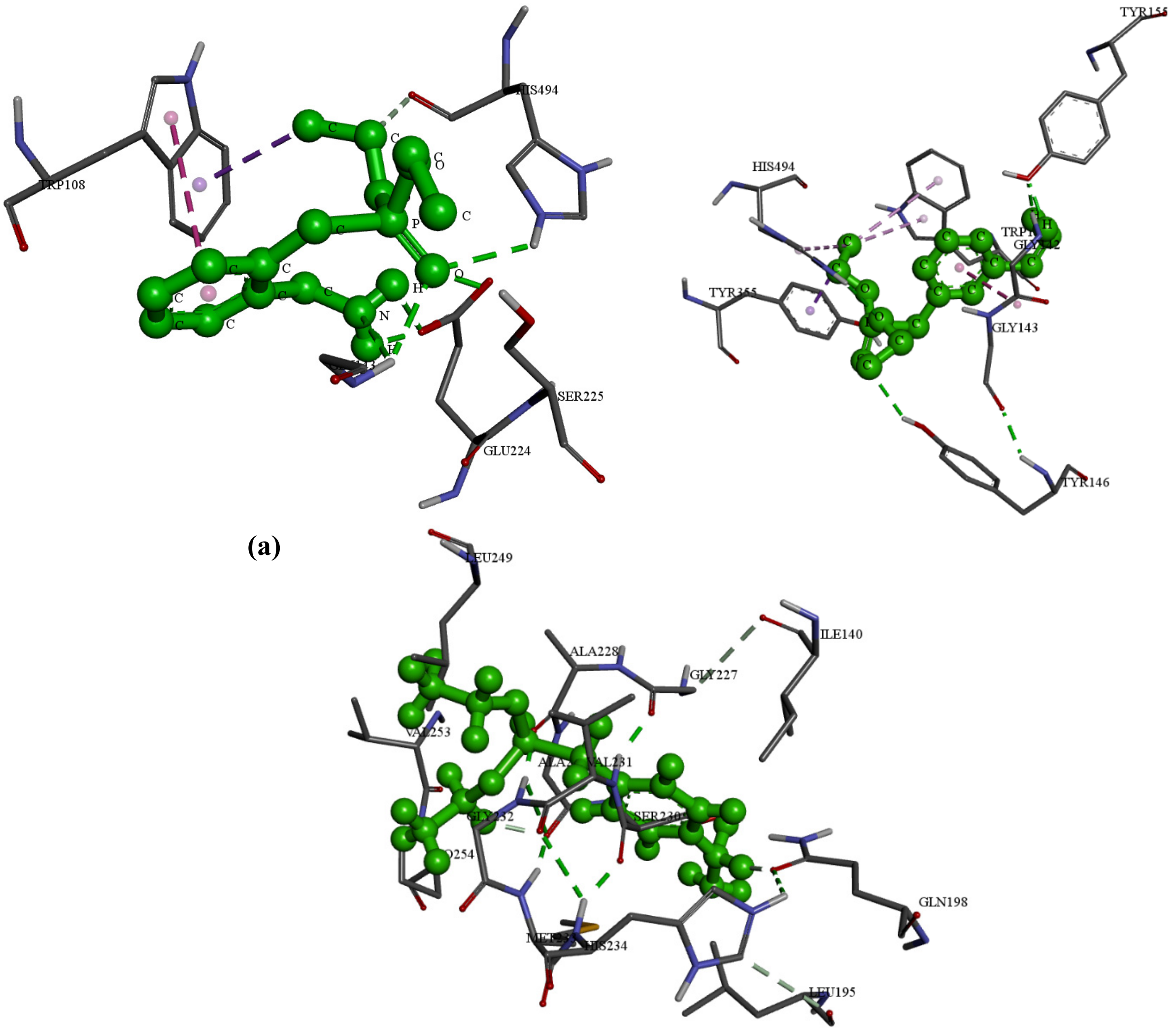

(a)

(c)

Supplementary Figure S63. The hydrogen bond formation between the active sites residues of the eeAChE and ligands **8a**, **8b** and **8c** respectively.

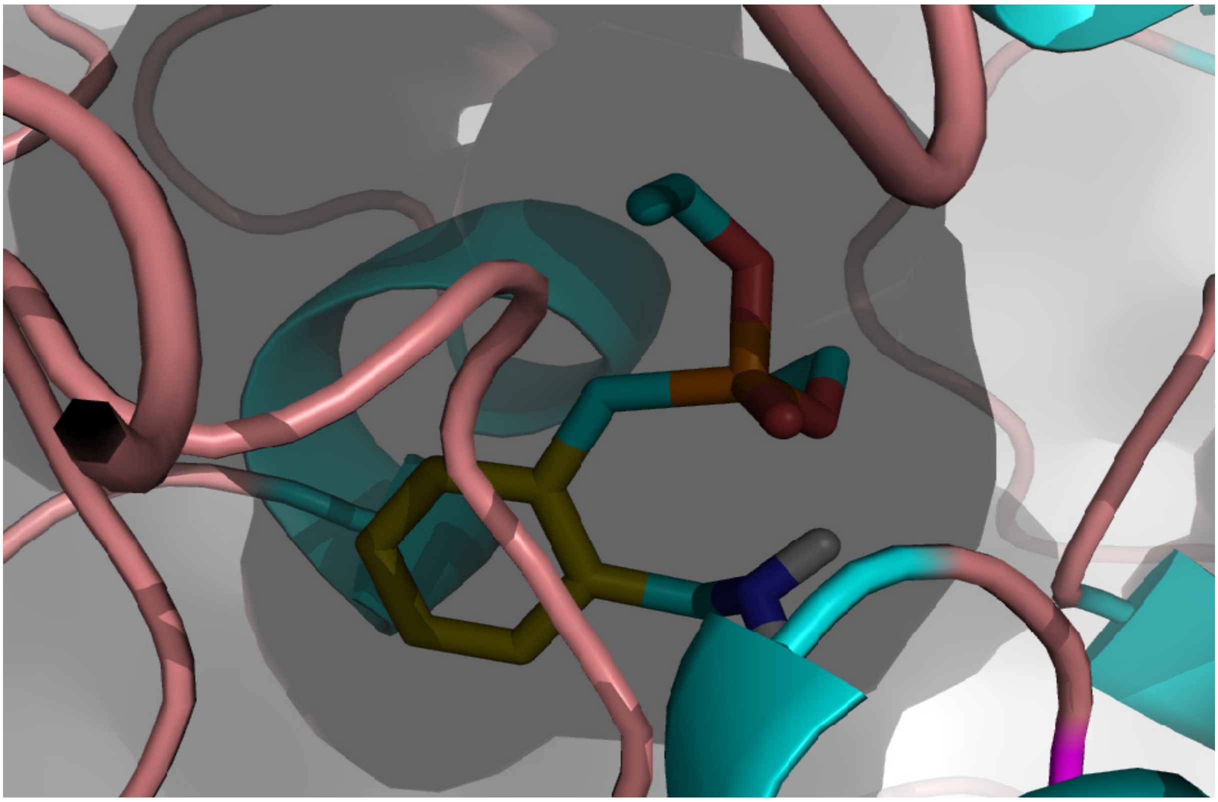

Supplementary Figure S64. Structure of the ligand **8a** in the active side of the eeAChE enzyme from electric eel (modified structure of pdb code 1C2B).

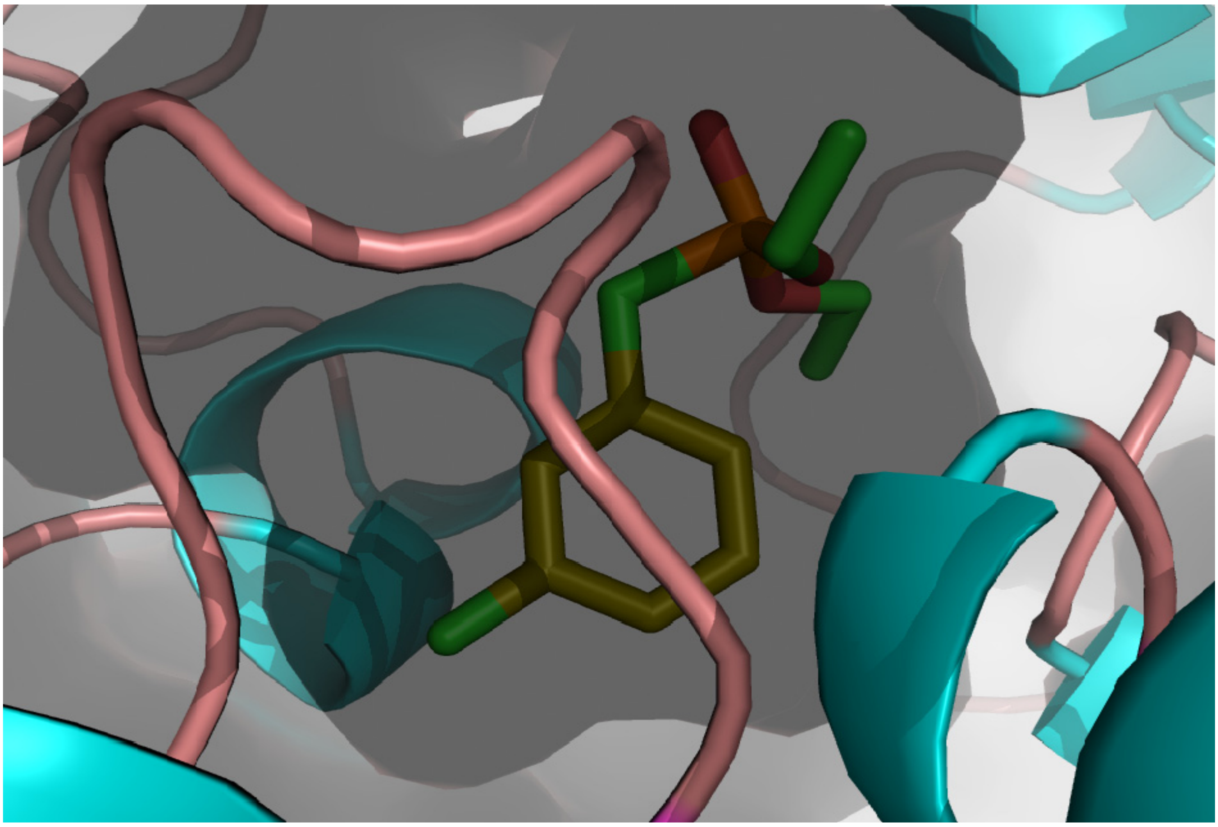

Supplementary Figure S65. Structure of the ligand **8b** in the active side of the AChE enzyme from electric eel (modified structure of pdb code 1C2B).

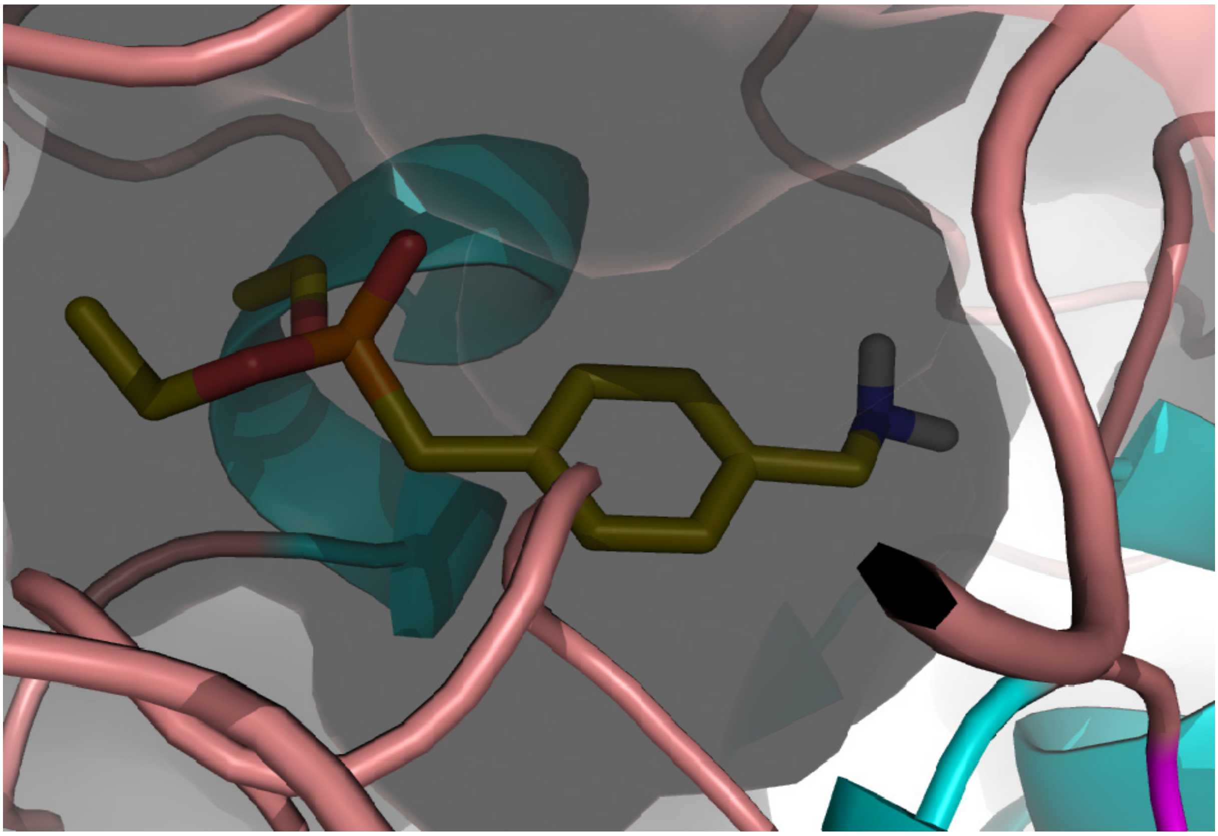

Supplementary Figure S66. Structure of the ligand **8c** in the active side of the AChE enzyme from electric eel (modified structure of pdb code 1C2B).

Supplementary Table S6. The docking score of the highest models from each ligand.

	8a	8b	8c
**Docking score**	-7.80	-6.36	-6.74
	-7.75	-6.31	-6.29
	-7.43	-6.03	-5.37
	-7.18	-5.94	-4.83
	-7.11	-5.76	-4.01
